# Influence of global climate modes on wildfire occurrence in the contiguous United States under recent and future climates

**DOI:** 10.1007/s00382-025-07998-w

**Published:** 2025-12-19

**Authors:** Theodore R. Keeping, Theodore G. Shepherd, I. Colin Prentice, Karin van der Wiel, Sandy P. Harrison

**Affiliations:** 1 Centre for Environmental Policy, Imperial College London, London, UK; 2 Geography & Environmental Science, University of Reading, Reading, UK; 3 Leverhulme Centre for Wildfires, Environment and Society, Imperial College London, London, UK; 4 Department of Meteorology, University of Reading, Reading, UK; 5 Georgina Mace Centre for the Living Planet, Department of Life Sciences, Imperial College London, Ascot, UK; 6 Royal Netherlands Meteorological Institute (KNMI), De Bilt, The Netherlands

**Keywords:** Wildfire, Climate modes, Teleconnections, Wildfire modelling, Climate variability

## Abstract

Predictable modes of climate variability, such as the El Niño Southern Oscillation (ENSO), have a major influence on regional weather patterns, an important control on wildfire occurrence. Although these global climate modes have been associated with historical variability in wildfire occurrence in the United States and are used to forecast seasonal wildfire risk, precise information about the spatial pattern and magnitude of their influence is lacking and the satellite record of wildfires is too short to address these issues. Here we use wildfire occurrence model with a large ensemble of 1600 simulated years from EC-Earth3 in a recent climate (2000–2009) and a future climate corresponding to + 2 °C global warming, to characterise the impact of specific climate modes on wildfire occurrence in the contiguous US. We show that ENSO, the Indian Ocean Dipole (IOD), and the 1-year lagged Tropical North Atlantic (TNA+1) have the greatest effect on annual fire occurrence—strongly contributed by the effect of these modes on hot, dry conditions in the Great Plains and precipitation in the southwestern US. El Niño is not significantly associated with wildfire occurrence in the northwestern US, contrary to expectation, but is associated with a later (earlier) wildfire season peak in the southwestern (southeastern) US. Under future warming, the AMO and PNA become a significant influence over most of the US, and the magnitude of impact of ENSO and TNA+1 increase strongly.

**Supplementary Information:**

The online version contains supplementary material available at 10.1007/s00382-025-07998-w.

## Introduction

With climate change, extreme wildfires are occurring at a greater frequency and intensity (Cunningham et al. 2024). Severe fire years often occur when synoptic-scale hot and dry weather events cause extremely wildfire-prone conditions (Gedalof et al. 2005; Barnes et al. 2025), resulting in multiple large wildfire events; the Australian 2019–2020 (NSW EPA, 2021) and the Canadian 2023 (Pelletier et al. 2024) fire seasons are examples of this. Whilst the occurrence of such conditions is generally increasing with climate change, there is a high variability in wildfire activity and its climatic drivers between years (Abatzoglou et al. 2018). This interannual variability is a key property of the wildfire regime, and in the United States (US)—the focus of this study—there are strong geospatial patterns in annual wildfire variability distinct from the mean rate of wildfire (Keeping et al. 2025).

Global modes of climate variability, such as the El Niño Southern Oscillation (ENSO), have been linked to fire year variability. In additional to natural stochasticity in wildfire outcomes, climate variability explains much of the interannual variability in burnt area globally (Abatzoglou et al. 2018; Gincheva et al. 2024). Approximately half of global burnt area is modulated by climate modes (Chen et al. 2016; Cardil et al. 2023) through their influence on rainfall, temperature and spring onset (Dai and Wigley 2000; Abram et al. 2014; Schwartz et al. 2013). In the US, ENSO and other climate modes have been shown to have a significant influence on wildfire danger (Mason et al. 2017). Climate modes can be used to forecast seasonal wildfire danger (Shen et al. 2019), and ENSO and the Pacific Decadal Oscillation (PDO) are adopted in the published seasonal outlook by the US government (NIFC 2024a).

There is an extensive tree-ring literature linking historic wildfire events in the western US (west of the 100˚W meridian) and climate modes (see Supplementary Sect. 1 for more details), primarily focusing on ENSO, the PDO, and the Atlantic Multidecadal Oscillation (AMO). These studies often cover multiple centuries, correlating reconstructed climate modes with tree-ring fire scars. The primary influence on wildfire in the southwestern US is ENSO: tree-ring reconstructions (Kitzberger et al. 2007; Margolis and Swetnam 2013; Swetnam and Betancourt 1990; Westerling and Swetnam 2003) link La Niña years to drought and a higher probability of wildfire. Reanalysis-based studies find the same effect (Mason et al. 2017). Tree-ring reconstructions also link El Niño years to a later southwestern fire-season peak (Kitzberger et al. 2001). In the northwestern US, tree-ring studies link El Niño years to higher wildfire activity (Hessl et al. 2004; Johnston et al. 2017) through a reduction in precipitation (Westerling and Swetnam 2003). This effect is linked more strongly to wildfire size than to the rate of occurrence (Heyerdahl et al. 2002). Recent data support the link between El Niño and very large wildfires in this region (Barbero et al. 2015). However, a study of remotely sensed burnt area covering a shorter period but a larger area found that the influence of ENSO on wildfire in the western US is weak compared to other key relationships between wildfire and climate modes globally (Cardil et al. 2023).

The positive phase of the PDO (PDO+) has been linked to greater burnt area in the northwestern US in tree-ring analyses, especially when in conjunction with El Niño (Ascoli et al. 2020; Heyerdahl et al. 2002; Norman and Taylor 2003; Schoennagel et al. 2005). However, reconstructions of the PDO vary significantly and the effects on US wildfire depend on the specific reconstruction (Kipfmueller et al. 2012). Tree-ring reconstructions also associate the warm AMO+ with increased burnt area in the West, with studies primarily centred on the northwestern US (Ascoli et al. 2020; Kitzberger et al. 2007; Trouet et al. 2010). The positive Pacific/North American (PNA+) mode has also been associated with an earlier spring onset in the West (Ault et al. 2011; Dannenberg et al. 2018).

Tree-ring scars have not been used to reconstruct relationships between wildfire and modes in the southeastern and central US, but shorter timescale federal or state wildfire records have been used. In the southeastern US, state fire records indicate an association between La Niña years and a reduction in precipitation and an increase in burnt area in the early months of the year (Dixon et al. 2008; Goodrick and Hanley 2009; Simard et al. 1985). Remote sensing data support this finding (Cardil et al. 2021). The PNA- and PDO+ have also been linked with a limited increase in wildfire in the southeastern US (Dixon et al. 2008; Goodrick and Hanley 2009), whilst the North Atlantic Oscillation (NAO); Arctic Oscillation (AO) and East Atlantic (EA) climate modes are linked to higher evaporative demand in the Southeast—which can increase the likelihood of wildfire (Martens et al. 2018; Cardil et al. 2023). La Niña has also been linked to severe wildfire danger in the southern Great Plains (Lindley et al. 2014; NIFC 2024a) due to vegetation becoming drier in response to droughts (Puxley et al. 2024) associated with La Niña events (Schubert et al. 2004). However, there are no studies based on long records that firmly establish a link between wildfire and ENSO or any other climate mode in the region.

Site-based tree-ring records can be used to identify robust, long-term relationships between climate modes and wildfire, but are limited in their geographical coverage. Remotely sensed burned area or state fire records provide more continuous geographical coverage but the small sample size, given the highly stochastic nature of wildfire events, both reduces the probability of obtaining statistically significant relationships between wildfire and climate modes, and introduces a higher risk of spurious correlations due to random variability, especially for longer period modes.

The lack of statistically significant relationships between wildfire and climate modes during the past three decades either in observed (Short et al., 2022; Supplementary Figs. 2.2, 2.4) or reanalysis-driven modelled wildfires (Supplementary Figs. 2.1, 2.2) reflects the small sample size. Large ensemble (LE) methods, widely used to study other climate impacts (Coburn et al. 2024; Swain et al. 2020; Lopez et al. 2018), overcome the sample-size issue and provide an alternative way of quantifying the relationships between wildfire and climate modes. Thus, using an LE together with a probabilistic model of wildfire occurrence facilitates an assessment of the geographical variation in the relationship between climate modes and wildfire, the strength of these relationships, and how they may be affected by climate change. Here, we investigate the effect of climate modes on US wildfire based on a 1600-year ensemble of modelled annual wildfire occurrence in the contiguous US for two decade-long time slices: the recent (2000–2009) climate and a future climate subject to an additional + 2 °C global warming. We first identify the most influential modes based on their areal impact under recent climate conditions, and show that their effect is physically plausible. Next, we examine how the magnitude of their effect varies geographically and how they influence fire season length and timing. We also test multivariate effects with ENSO. Finally, we examine how future climate change reduces or increases the area affected and the magnitude of that impact.

## Data and methods

### The wildfire occurrence model

We used a wildfire occurrence model (full description—Keeping et al. 2024) trained on wildfire occurrence data (Short et al. 2022) to model the daily probability of a wildfire greater than 0.1 hectares in extent at 0.1° spatial resolution. This model uses a generalised linear modelling framework but employs a flexible variable selection algorithm to find the optimal set of predictors from a suite of candidate variables related to climate, vegetation, and human factors influencing wildfire, and then optimises the domain of influence of each of the selected variables. In the original derivation of the model 47 candidate predictors were used, but here we retrained the model starting from 31 candidate predictors for which temporally-varying data were available (per Keeping et al. 2025).

The final selected predictors were cropland fraction, needleleaf fraction, shrub fraction, gross primary production (GPP) in the previous 50 days and the previous year, rural population density, diurnal temperature range, precipitation on that day and in the previous five days, mean daytime windspeed, snow cover fraction, mean daytime vapour pressure deficit (VPD). Lightning ignitions were not included in the model; including convective atmospheric potential energy as a predictor of lightning was assessed but not selected. Meteorological and vegetation properties influence both fuel availability and fuel drying. The inclusion of two GPP terms takes account of both recent and longer-term fuel accumulation. The inclusion of cropland fraction and population density implicitly account for human impacts on wildfire occurrence through ignitions and fragmentation. Although fuel removal or the legacies of fire suppression on fuel accumulation are not taken into account explicitly, these are implicit in so far as they are reflected in the fire occurrence data on which the model was trained. The domain over which each variable influences wildfire likelihood was optimised separately. The outputs are then power-law rescaled to minimise the tendency for generalised linear models to underestimate wildfire extremes (Forrest et al. 2024). The model is applied in the recent climate (overlapping with the training period) and in a future climate subject to + 2 °C global warming. At coarser spatial and temporal resolutions, this could create bias due to out of sample future conditions. However, because the model is trained on daily data across all environments in the contiguous US, almost all days and locations in the + 2 °C time-slice will have an analogue, or near analogue, in the training data.

The model was tested against wildfire occurrence data (Short et al. 2022) and, when run using reanalysis data (1992–2020), showed good discrimination in its predictions of wildfire events. The reduced variable model performed within the range of the Pareto superior subset of original model training runs (Keeping et al. 2024) across all benchmarks. The area under the receiver operating curve (AUC) score is 0.89, substantially greater than the 0.8 value considered to indicate a good model (McCune et al. 2002). It also reproduced the geographic patterns in wildfire occurrence (Supplementary Fig. 3.1; normalised mean error, NME = 0.46), as well as the seasonal concentration (NME = 0.78) and timing of the wildfire season (mean phase difference = 0.13) and the interannual variability (NME = 0.67) in the number of wildfires.

### KNMI-LENTIS derived inputs and bias correction

KNMI-LENTIS (Muntjewerf et al. 2023) is a time-slice single-model initial-condition large ensemble of the EC-Earth3 climate model (Döscher et al. 2022). The pre-industrial spin-up was sampled at 25-year intervals to obtain starting points for 16 transient simulations that were run from the pre-industrial (1850 CE) to the end of the twenty-first century with historical and SSP2-4.5 forcings. Ensemble members were then derived for 2000–2009 (referred to here as recent) and 2075–2084 (referred to here as future and corresponding to approximately + 2 °C additional global warming compared to the recent climate), by subjecting each of the 16 transient runs to nine micro-perturbations in global temperature (< 5·10–5 K) at the start of each decade. Together with the original transient run, this yielded 10 decade-long simulations, providing 160 ensemble members for each time slice. The 25-year sampling of the macro-perturbations ensures a good sampling of decadal to multidecadal climate oscillations such as the AMO in each ensemble time-slice. Shorter period oscillations such as ENSO are understood to diverge based on initial conditions within a year (Neelin 2010). EC-Earth3 represents historical trends in precipitation, land-surface temperature and blocking-frequency over the contiguous US well (Döscher et al. 2022). The version used in KNMI-LENTIS was further tuned to improve model performance in the northern hemisphere by reducing a cold bias (Muntjewerf et al. 2023).

The climate predictors from KNMI-LENTIS needed for the wildfire occurrence model were bias- corrected using ERA5-Land reanalysis data (Muñoz-Sabater et al. 2021) and downscaled to 0.1° following the methodology used in Keeping et al. (2025) (Supplementary Sect. 4). GPP was predicted using a light-use efficiency model (the P modelWang et al. 2017; Stocker et al. 2020) that simulates photosynthesis, accounting for temporal acclimation of carboxylation and stomatal conductance to environmental conditions. The temperature, VPD, air pressure, incident photosynthetic flux density, and CO_2_ concentration inputs to the P model were taken from the bias-corrected and downscaled KNMI-LENTIS ensemble. The fraction of absorbed photosynthetically active radiation (fAPAR) was derived using Beer’s law from simulations of the seasonal cycle of leaf area index (LAI), based on the reciprocity between LAI and GPP (Zhou et al. 2024). Annual antecedent GPP is used in the wildfire occurrence model; to calculate this for the first year, the first year of each decade in the climate ensemble was repeated (following Van der Wiel et al. 2019).

### Climate mode calculation

We initially considered all climate modes thought to influence wildfire danger or evaporative demand over the contiguous US with an annual or longer oscillation timescale, based on previous literature. Climate modes were derived using monthly sea-level pressure (SLP) and sea surface temperature (SST) fields from KNMI-LENTIS. Geopotential height is often used to calculate pressure-based climate modes but was not available for KNMI-LENTIS, so SLP was used instead. Climate mode indices were calculated separately in the recent and + 2 °C ensembles, in order to represent the effects of variability within the two climates. Modes derived using principal components were checked to ensure they showed the correct sign of effect on their associated SLP or SST trends. The phenomena associated with each climate mode (as defined in Table 1) and their effect on US meteorology are both well-represented compared to observations—refer to Supplementary Sect. 5 for a complete overview.

**Table 1:** An overview of the 11 modes considered in this analysis, with the information given on the variable used; the method by which each monthly mode is calculated; and the method by which the annual value of the mode was found

Mode	Index	Field	Monthly calculation method	Annual Index calculation
El Niño Southern Oscillation (ENSO)	Equatorial Southern Oscillation Index (SOI)	SLP	The difference between the monthly Indo-Pacific [5°S-5°N, 90°E-140°E] and Eastern Pacific [5°S-5°N, 80°W-130°W] standardised anomalies. NOAA (2009)	Monthly values averaged annually
Indian Ocean Dipole (IOD)	Dipole Mode Index (DMI)	SST	The standardised difference between the West [10°S-10°N, 50°E-70°E] and East [10°S-0°N, 90°E-110°E] Indian Ocean anomalies. Saji and Yamagata (2003)	Monthly values averaged annually
Pacific Decadal Oscillation (PDO)	PDO Index	SST	The leading principal component of the SST anomaly over the North Pacific [20°N-70°N, 120°E-100°W]. Newman et al. (2016)	The November to March (NDJFM) mean of the index starting in the previous year
Tropical North Atlantic (TNA)	TNA Index	SST	The monthly anomaly in the North Tropical Atlantic [5°N-25°N, 55°W-15°W]. Enfield et al. (1999)	Monthly values averaged annually
Tropical South Atlantic (TSA)	TSA Index	SST	The monthly anomaly in the South Tropical Atlantic [20°S-0°N, 30°W-10°E]. Enfield et al. (1999)	Monthly values averaged annually
North Atlantic Oscillation (NAO)	NAO Index	SLP	The leading principal component of the monthly anomaly over the North Atlantic [20°N-80°N, 90°W-40°E]. Thornton et al. (2023)	The December to February (DJF) mean of the index starting in the previous year
East Atlantic (EA)	EA Index	SLP	The second principal component of the monthly anomaly over the North Atlantic [20°N-80°N, 90°W-40°E]. Thornton et al. (2023)	The November to January (NDJ) mean of the index starting in the previous year
Arctic Oscillation (AO)	AO Index	SLP	The leading principal component of the monthly anomaly north of 20°N. NOAA (2025)	The DJF mean of the index starting in the previous year
Pacific/North American (PNA)	PNA Index	SLP	The leading principal component of the DJF-mean of monthly anomalies over the North Pacific and North America [20°–90°N, 120°E − 120°W]. Mori et al. (2024)	The calculation of the DJF mean, starting in the previous year, was taken before the leading principal component was calculated
Atlantic Multidecadal Oscillation (AMO)	AMO Index	SST	The difference between the annual SST anomalies of the Atlantic [0°N-60°N, 75°W-7.5°W] and the rest of the global ocean. Enfield et al. (2001)	N/A
Southern Annular Mode (SAM)	SAM Index	SLP	The leading principal component of anomalised SLP south of 20°S. NOAA (2025)	The June to August (JJA) mean

### Comparison of climate mode effect on US weather in LE and reanalysis

**Fig. 1:**
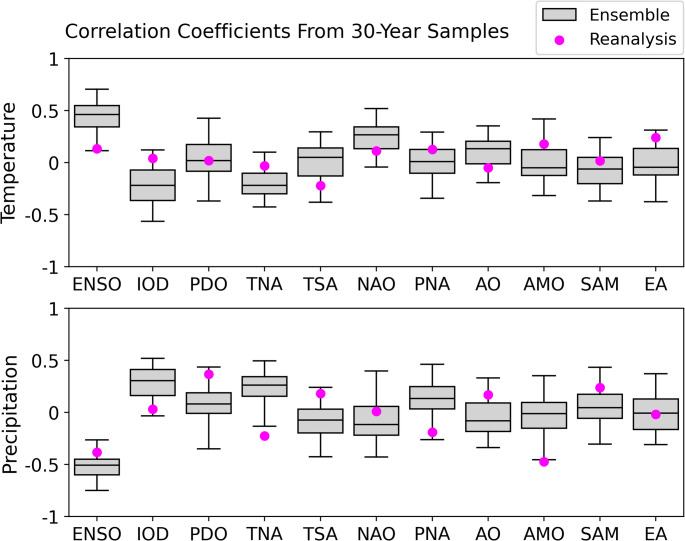
Boxplots of each quartile of the distribution of correlation coefficients between climate mode indices and temperature (upper panel) and precipitation (lower panel) over samples of the recent climate ensemble, and the values from the reanalysis over 1990–2019 (pink dots). To construct the distribution behind the box plots, random samples of 30 years were taken from the ensemble. The outer limits of the boxplots represent the 2.5th and 97.5th percentiles, and the three internal lines correspond to the 25th, 50th and 75th percentiles of the bootstrapped distribution

A high-resolution wildfire occurrence record is available in the contiguous US from satellite data after 1984 (Eidenshink et al. 2007) or from aggregated state and federal records after 1992 (Short 2022). This short reanalysis period is not sufficient to capture the major effects of each climate mode over the contiguous US (Fig. 1). The correlation coefficients of climate mode indices and US temperature and precipitation show that there is a large spread in the apparent correlation when 30-year samples from the ensemble are drawn. In most cases, the sign of the observed correlation could be switched and it would still lie within the 95% confidence interval of the model ensemble. The one clear exception is ENSO (note that our ENSO index is the SO index, so the sign is opposite to an SST-based index), where the sign is clear although the magnitude is highly uncertain. This short time period is therefore insufficient to robustly characterise the effect of each mode—justifying the use of modelled wildfires driven by the LE. EC-Earth3 performs well in its representation of ENSO, the NAO and the PNA (Döscher et al. 2022) which are all key controls on North American weather patterns. Comparison of annual US weather and climate mode values between the reanalysis and ensemble (Supplementary Fig. 6.2) shows no apparent discrepancies, and strong associations between modes (the TNA, IOD and ENSO) are equally present in the reanalysis and ensemble data (Supplementary Fig. 6.1).

### Relating climate modes to annual wildfires

The daily ensemble of modelled wildfire occurrence probabilities was averaged annually for comparison with the yearly phase of each climate mode—though seasonal responses were also checked, see Supplementary Sect. 7. The positive and negative phase of each climate mode was defined as occurring when its annual-mean index value was a half-standard deviation greater or lesser than zero, respectively; and was otherwise considered to be neutral. Simulated annual wildfire occurrence, aggregated to 0.5°, was regressed against the numerical value of each climate mode index. The relationship found by this regression was only considered in the analysis if it passed a false discovery rate corrected significance level of 0.01, per Wilks (2016). The sign of the climate mode’s effect was determined from the regression slope coefficient. The lagged effect of each index on wildfire was also tested by using the index value from the previous year. When mapping geographic patterns in the magnitude of each mode’s association with wildfires, the ratio between the number of modelled wildfires in each phase of the mode relative to the mean number at that grid-cell was plotted.

### Definition of fire season peak and length

To determine the effect of climate modes on the peak timing of the fire season, the seasonality was characterised according to Kelley et al. (2013). The wildfire season’s mean phase was determined for each grid-cell from the sum of monthly vectors oriented in the complex plane with angles corresponding to the time of year and magnitude proportional to the number of wildfires. The seasonal concentration was defined as the length of that resultant vector, varying between 0 (wildfires equally spread between months) and 1 (all wildfires in one month). The effect on the timing of the seasonal peak was calculated according to the difference between the mean phase over all years and over years in the positive or negative phase of each index. Locations with a seasonal concentration of less than 0.15 were not considered to have a distinct peak, so were excluded. The effect of climate modes on the length of the fire season was also determined relative to the average annual peak in the fire season, or the ensemble mean of the maximum wildfire month at each location. The mean number of months for which the number of wildfires exceeded half of that average annual peak was defined as the length of the fire season (per Jolly et al. 2015; Abatzoglou et al. 2019).

## Results

### Survey of the modes

**Fig. 2:**
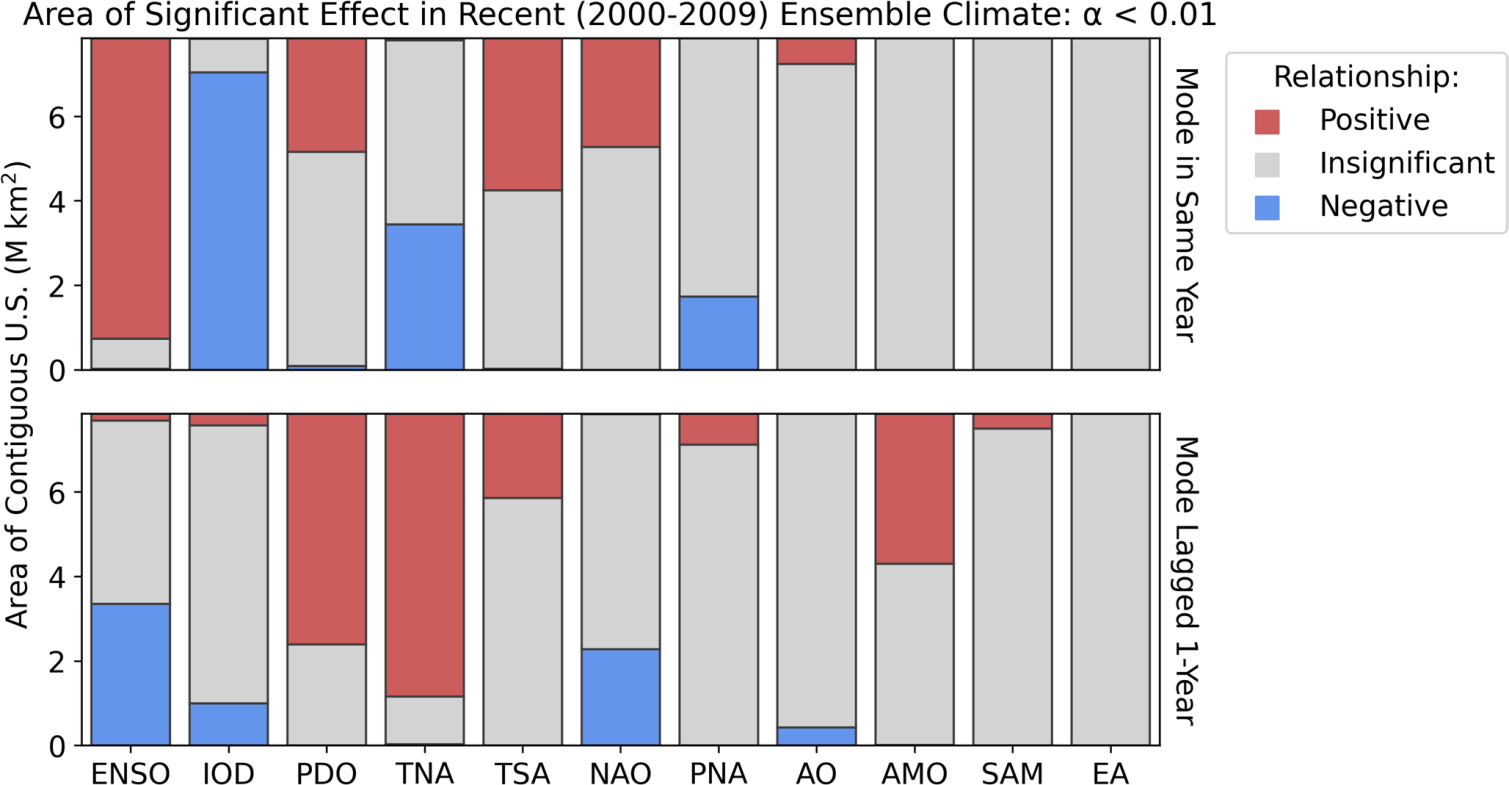
The area of the contiguous US significantly affected by each climate mode. Significance was determined using an FDR corrected significance threshold after Wilks (2016) to a control level of 0.01. The sign of the relationship between the index and the annual number of wildfires is given by the slope of the regression

The climate modes that show the greatest area of significant (*p*-value of < 0.01 after controlling for multiple testing) association with wildfire (Fig. 2, Table 2) are ENSO, the IOD and annually lagged TNA (TNA+1). The La Niña phase of ENSO has a positive influence on wildfire probability over 91% of the contiguous US. The negative IOD and positive TNA+1 have similarly large areas of significant influence, affecting 90% and 85% respectively. Both of these modes are correlated with (correlation coefficients of -0.61 and -0.32 in the recent ensemble) and causally related to ENSO (Ham et al. 2017; Jiang and Li 2019). The robustness of the influence of these three modes on US wildfire (Supplementary Sect. 7), taking a stricter significance threshold of 5.7 × 10^–7^ (the 5-sigma p-value for a Gaussian distribution), shows that the areal influence of ENSO, IOD and TNA+1 is persistent over most of the US.

**Table 2:** Percentage of contiguous US area significantly affected by each mode in the recent climate (2000–2009). Two asterisks indicate an area over 50%, one asterisk an area over 20%

Mode	Area of effect (Same Year) [%]			Area of effect (Prior Year) [%]		
	N/A	Positive	Negative	N/A	Positive	Negative
ENSO	9.0	**90.7	0.4	55.3	2.0	*42.7
IOD	10.1	0.2	**89.8	83.7	3.5	12.8
PDO	64.5	*34.3	1.2	30.4	**69.6	0.0
TNA	55.5	0.6	*43.8	14.3	**85.3	0.4
TSA	53.8	*46.0	0.3	74.6	*25.4	0.1
NAO	67.2	*32.8	0.0	70.8	0.1	*29.1
PNA	77.8	0.0	*22.2	90.7	9.3	0.0
AO	92.1	7.9	0.0	94.5	0.0	5.5
AMO	100.0	0.0	0.0	54.7	*45.2	0.1
SAM	100.0	0.0	0.0	95.5	4.5	0.0
EA	100.0	0.0	0.0	100.0	0.0	0.0

Other climate modes exert a significant influence on wildfire but over more limited regions. Eleven modes have a significant influence over an area > 20% of the contiguous US: ENSO, IOD, TNA+1, PDO+1, ENSO+1, TNA, AMO+1, TSA, PDO, NAO, and NAO+1. At the stricter significance threshold (Supplementary Sect. 7), the only modes showing persistent areas of influence on wildfires other than the TNA, IOD and ENSO are the PDO, AMO and NAO. The PDO also has the next most significant control on the distribution of the annual number of wildfires. Of the top eleven modes in areal significance, the TNA, PDO, NAO and ENSO influence wildfire both in the same year and with a one-year lag. For geographical analysis (Figs. 4 and 9) we consider the top eight modes by areal influence in the recent time-slice of the LE, eliminating the less influential of each of the unlagged or lagged modes. The top eight modes were ENSO, IOD, TNA+1, PDO+1, AMO+1, TSA, NAO, and PNA. The area influenced by these modes is greatest in the summer, June–August, except for the TSA which has the greatest influence in the spring, March–May (Supplementary Sect. 7). The AO only influences a small area (8%) and the SAM+1 only influences 5% of the contiguous US. The EA does not have a significant areal impact.

### Effect of key modes

**Fig. 3:**
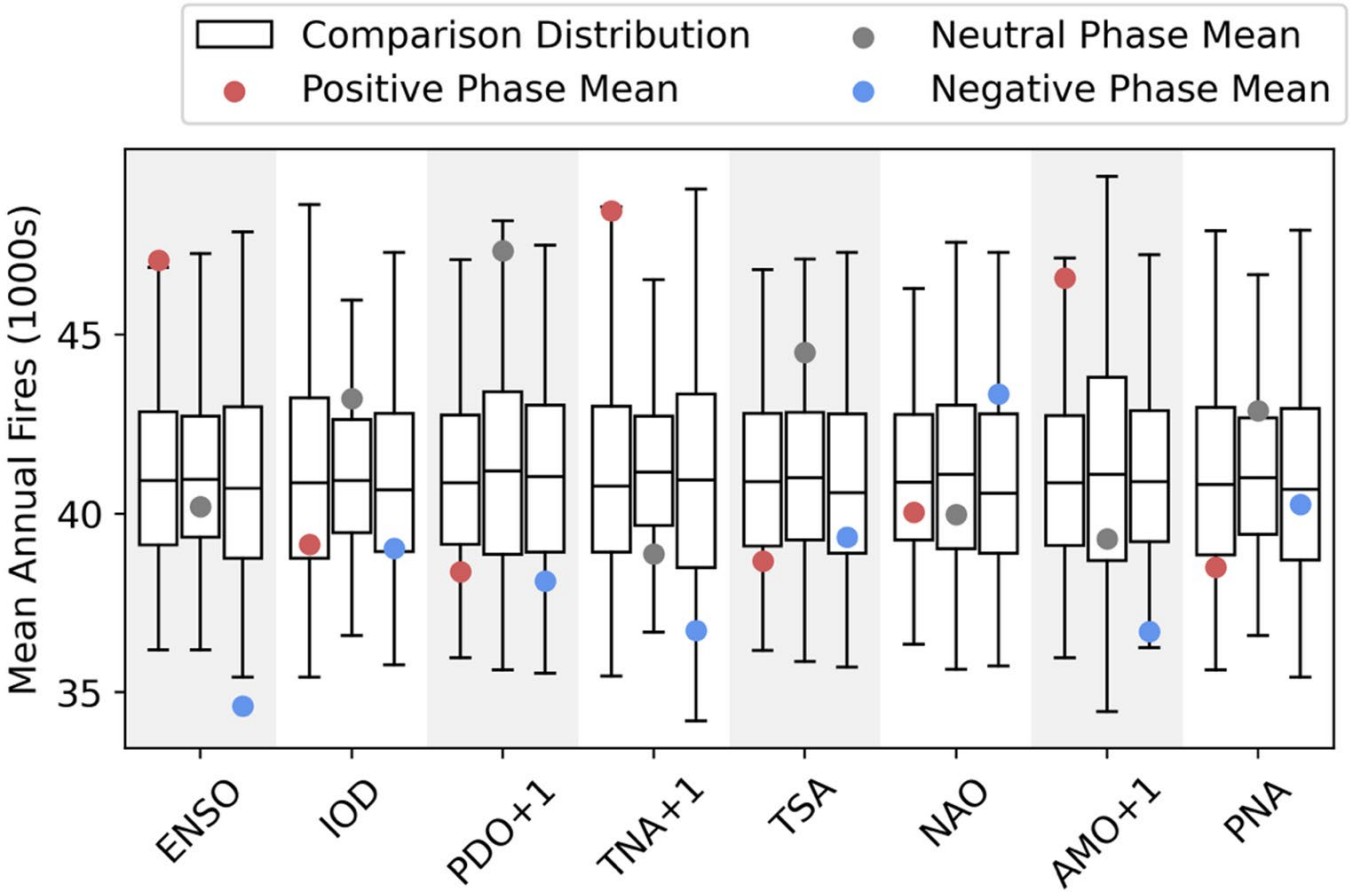
*The distribution of the modelled annual number of wildfires for the reanalysis (1990–2019) under different mode phases compared to the distribution of all years, per *Shen et al. (2025)*. The dots show the actual mean value of modelled annual fires. The boxplots show the distribution of the mean number of annual fires, drawn from the distribution of years but with the same sample size as the number of years in that phase in the reanalysis period*—*repeated 10,000 times. The outer limits of the boxplots represent the 2.5th and 97.5th percentiles, and the three internal lines correspond to the 25th, 50th and 75th percentiles of the bootstrapped distribution*

Given the plausibility of the effect of climate modes on US weather in EC-Earth3 (Fig. 1, Supplementary Sect. 5) and the considerable extent of the effect of those modes on US wildfire (Fig. 2, Table 2), there is a pertinent question as to the additional utility of the LE approach compared to reanalysis or observed data. There is no location in the US where a climate mode has a significant relationship with observed annual fire occurrences (Supplementary Fig. 2.3). Reanalysis-driven modelled fire occurrences also show almost no area of effect (Supplementary Fig. 2.1). Of the modes with the largest areas of statistically significant effect, very few climate mode phases result in an effect in the reanalysis significantly outside the distribution of random noise (Fig. 3). Only three mode phases are associated with annual wildfire numbers outside of the 95% confidence interval of the bootstrapped distribution from all years. In: La Niña years (positive ENSO phase), El Niño years (negative ENSO phase), and when the prior year had a positive TNA phase. The extent to which these phases exceed the confidence interval is very low in comparison to the highly significant margin provided by the LE (Supplementary Fig. 2.5), due to the different sample sizes.

**Fig. 4:**
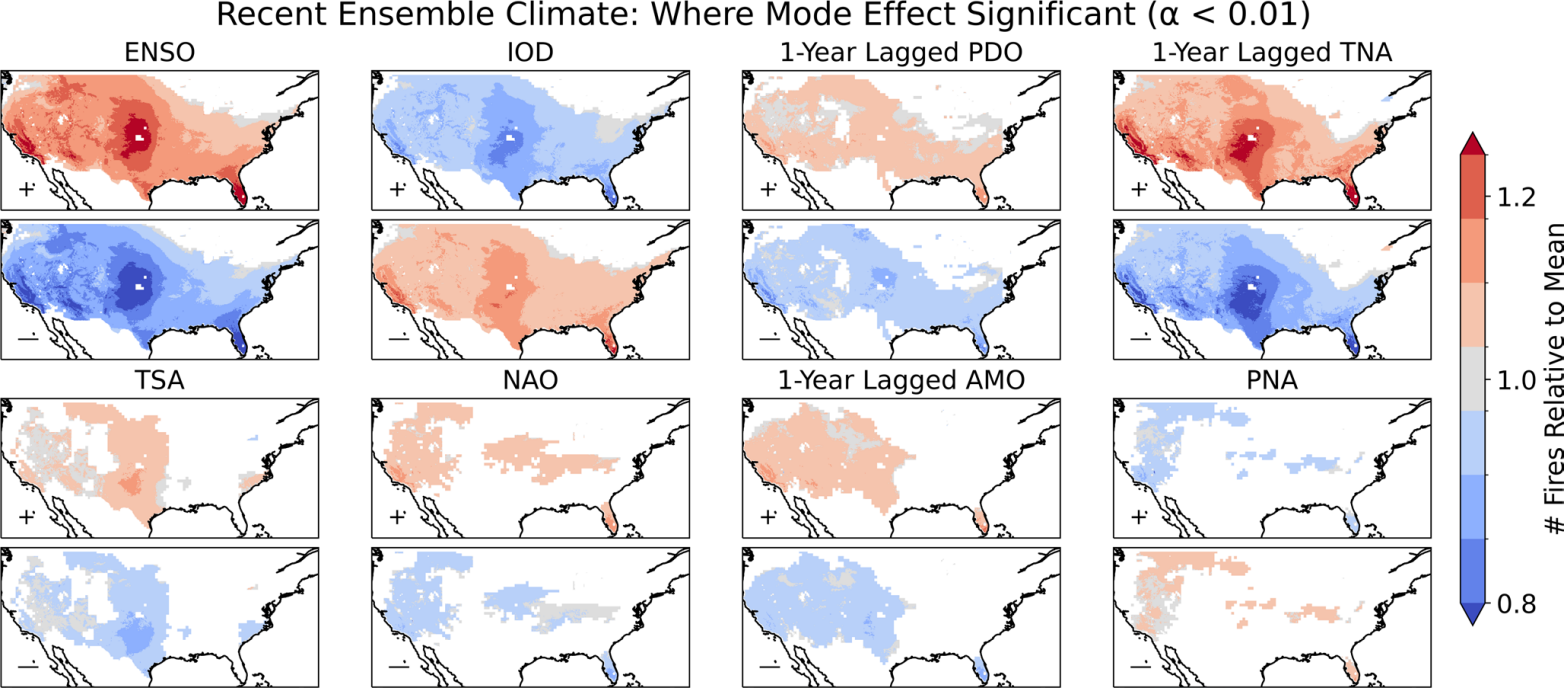
Maps of the effect of each climate mode on annual wildfires relative to the mean. The sign and magnitude of the relationship is given by the ratio between the annual number of wildfires in the positive (upper panels) or negative (lower panels) phase—defined as beyond plus or minus half a standard deviation from the mean respectively. The effect of each mode is shown relative to the mean annual number of wildfires—to account for any non-linearity in the effect between phases that would not be captured by linear regression. The relationship is only shown for locations where there is a significant relationship, as identified by linear regression between the index and annual number of wildfires at that location

ENSO has a positive effect on total annual US wildfires in the La Niña phase (Fig. 4), with the three areas of greatest relative increase in wildfire occurrence rates being Mediterranean California, the central Great Plains, and southern Florida. The only areas where ENSO has no significant influence on annual wildfire occurrence are in the northwestern and northeastern US. The negative IOD and positive TNA+1 show a very similar association with wildfire to La Niña. The NAO and PNA havepositive and negative impacts on annual wildfires in the southwestern and inland northwestern US respectively. The PDO+1 has a positive influence across the US. The AMO+1 affects a large area in the West and has a localised influence in southern Florida. ENSO+1 has a strong influence of wildfire occurrence along the southern US border (Supplementary Sect. 7)—through a control on GPP due to its association with precipitation in this region (see Fig. 6). The TNA also shows a substantial effect associated with an increased number of wildfires in the West (including in the northwestern US) and in Florida.

La Niña, the negative IOD and positive TNA+1 all show a very similar association with wildfire. The positive phase of the TNA+1 and negative phase of the IOD are associated with increased wildfire occurrences when considered alone (Fig. 4). In these phases, the total number of wildfires in the contiguous US is found to increase by 14% and 12% respectively (Table 3). However, both modes are correlated to ENSO (Supplementary Sect. 6), and the effect of both modes decreases substantially after controlling for the phase of ENSO, from 14 to 5–8% for the positive phase of the TNA+1 and from 12 to 2–3% for the negative phase of the IOD. The biggest impact of both the negative phase of the TNA+1 and positive phase of the IOD is on wildfire in La Niña years, where the mean number of wildfires is decreased by 12% and 9% respectively.

**Table 3:** The effect of the TNA+1 and IOD climate modes on the number of wildfires in the contiguous US in each phase of ENSO and for all years, in the recent climate time-slice of the ensemble. The sample size each percentage is based on is given in brackets

		# Fires (3 s.f.)	TNA+1 Phase		IOD Phase	
			Positive	Negative	Positive	Negative
ENSO Phase	La Niña	44,600	+ 7.7% (207)	-11.9% (71)	-8.8% (23)	+ 3.2% (294)
	Neutral	37,700	+ 4.9% (145)	-6.1% (169)	-1.4% (180)	+ 2.2% (141)
	El Niño	32,800	+ 6.1% (69)	-5.2% (210)	-0.9% (273)	+ 3.4% (72)
All Years		37,800	+ 13.6% (421)	-10.1% (450)	-8.4% (476)	+ 11.7% (507)

**Fig. 5:**
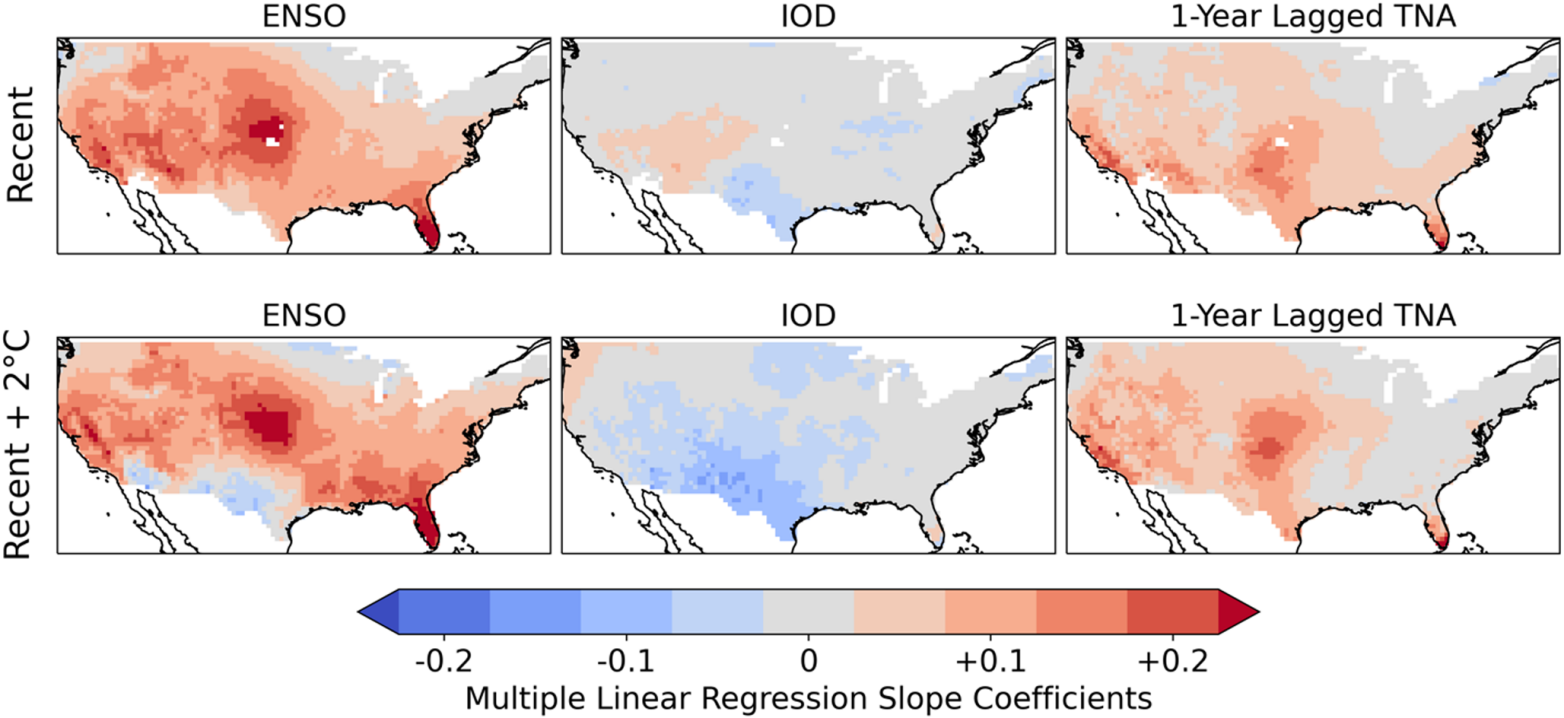
Multilinear regression of the annual number of wildfires relative to the mean at each grid cell against standardised values of the ENSO (SOI), IOD (DMI), and TNA+1 indices. The slope coefficients were separately calculated for each grid-cell, and are displayed for the recent and + 2°C ensemble climates

A multilinear regression (MLR) of annual wildfires (Fig. 5) against index values of ENSO, the IOD and TNA+1 finds that ENSO is the strongest pathway controlling interannual variability for wildfires in the contiguous US. The MLR slope coefficient for ENSO has the same pattern as the association between ENSO and wildfires when not controlling for the IOD or TNA+1. The TNA+1 also has substantial influence, having an additional effect on wildfires in Mediterranean California, the southwestern US, the southern Great Plains, and southern Florida. It follows a similar pattern of effect to the single-mode association of the TNA+1 and wildfires, though the magnitude of effect is smaller compared to ENSO. The IOD has a much weaker influence on wildfires, limited to low magnitude effects in the southwestern US.

**Fig. 6:**
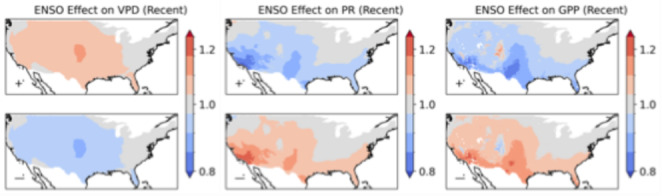
the relative influence of ENSO on VPD, precipitation and GPP in the recent climate for the large ensemble

The association between ENSO and wildfire occurrences reflects the influence of ENSO on VPD and precipitation, where precipitation is also strongly linked to annual patterns in vegetation growth and fuel accumulation (Fig. 6). The region associated with a strong wildfire occurrence response to ENSO in the Great Plains has a stronger VPD anomaly while in the southwestern US, the impact of ENSO on wildfire is associated with a stronger precipitation anomaly. Other modes also influence wildfires through their impacts on VPD, precipitation and GPP (Supplementary Fig. 8.1).

**Fig. 7:**
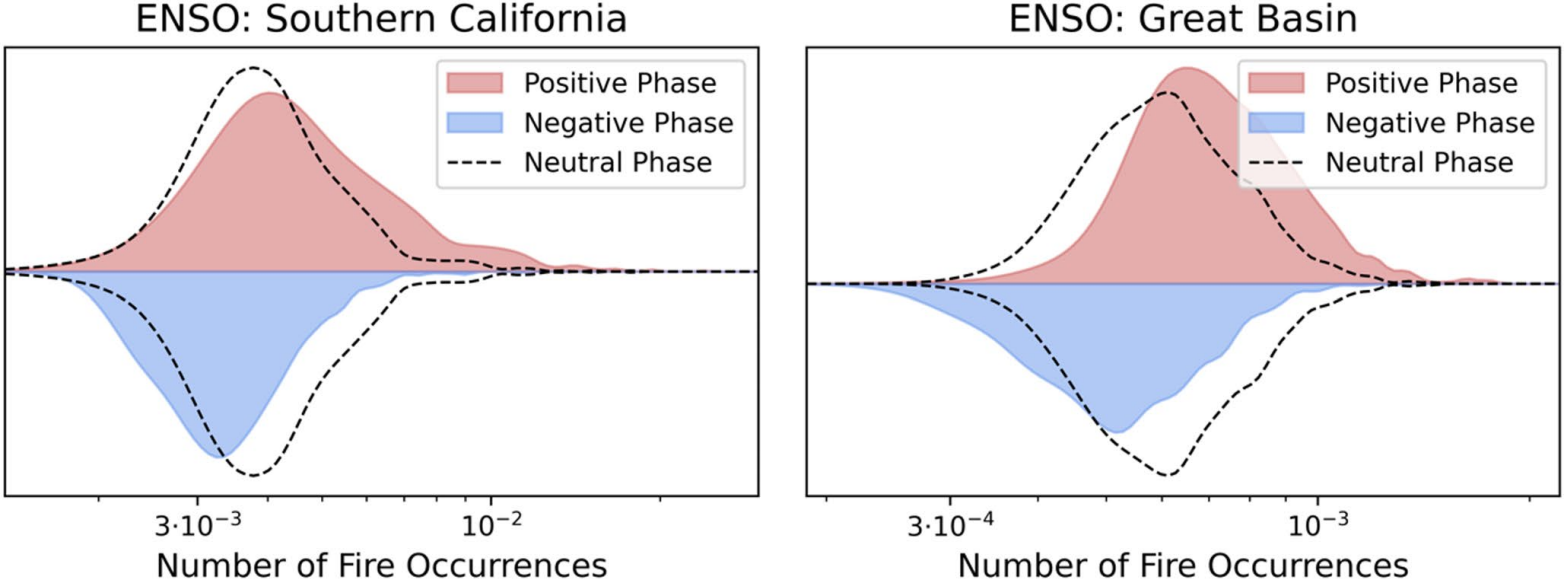
The frequency distribution of the modelled annual number of wildfires per km^2^ across the 1600 recent climate ensemble years in the Great Basin and Southern California NIFC Geographic Area Coordination Centres (GACC) defined regions (Supplementary Fig. 3.2). The distributions are plotted separately for the positive (La Niña) and negative (El Niño) phase of ENSO, with the neutral phase shown as a dashed line for comparison

The response of the statistical distribution of annual wildfire occurrence to a climate mode varies geographically (Fig. 7). The effect of ENSO on wildfires in the Great Basin, for example, produces a shift in the distribution but has limited impact on the spread. In contrast, the effect of ENSO in Southern California is to extend the spread of the distribution giving rise to an increase in the likelihood of extreme fire-years relative to the mean. There are also distinct geographic effects on wildfire distribution under ENSO, TNA+1, PNA, PDO+1 and IOD (Supplementary Sect. 9).

**Fig. 8:**
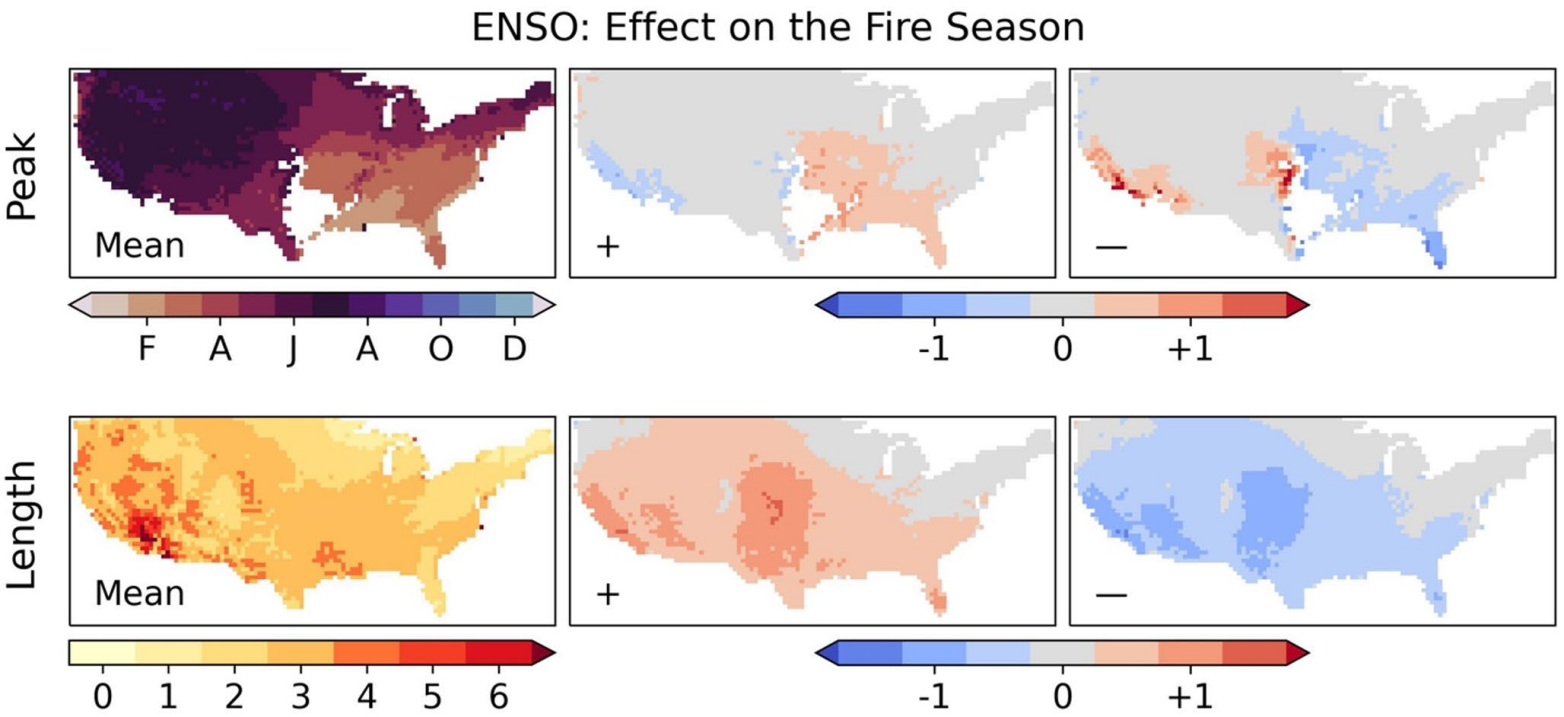
Top row: (left) the average seasonal phase across all locations with a seasonal concentration > 0.15; (central) the average seasonal phase for all years subtracted from the average of years in the positive (La Niña) phase; (right) the average seasonal phase for all years subtracted from the average of years in the negative (El Niño) phase. Bottom row: (left) the length of the fire season in months (calculated as the number of months over the mean annual half-maximum); (central) the average season length for all years subtracted from the average of years in the positive (La Niña) phase; (right) the average season length for all years subtracted from the average of years in the negative (El Niño) phase

Climate modes do not have a uniform effect on meteorology, or consequent wildfire danger, throughout the year (Supplementary Sect. 7). ENSO has a strong impact on the length of the wildfire season (Fig. 8), with increased season length in La Niña years in Mediterranean California, the Arizonan Mountains, the Great Plains and southern Florida. This contributes to the high annual numbers of wildfires in those regions (Fig. 4). The effect of ENSO on the peak of the fire season is less uniform and shows an east/west divide: El Niño (La Niña) results in an earlier (later) peak to the fire season in areas to the east of the southern Great Plains, but El Niño (La Niña) results in a later (earlier) peak in the southwestern US. The effect of El Niño on the timing of the seasonal peak is stronger than that of La Niña, with La Niña increasing the frequency of wildfire occurrence in both regions and bringing the early-spring (east) and late-summer (west) fire season peaks closer together. The IOD and TNA+1 have a very similar effect on fire seasonality to ENSO, whilst the remaining modes have limited effects on the fire seasonality (Supplementary Sect. 10), though the PDO+, PNA- and NAO+phases are associated with a half-month earlier peak to the Californian fire season.

### Changes in the effect of global climate modes in a warmer climate

**Fig. 9:**
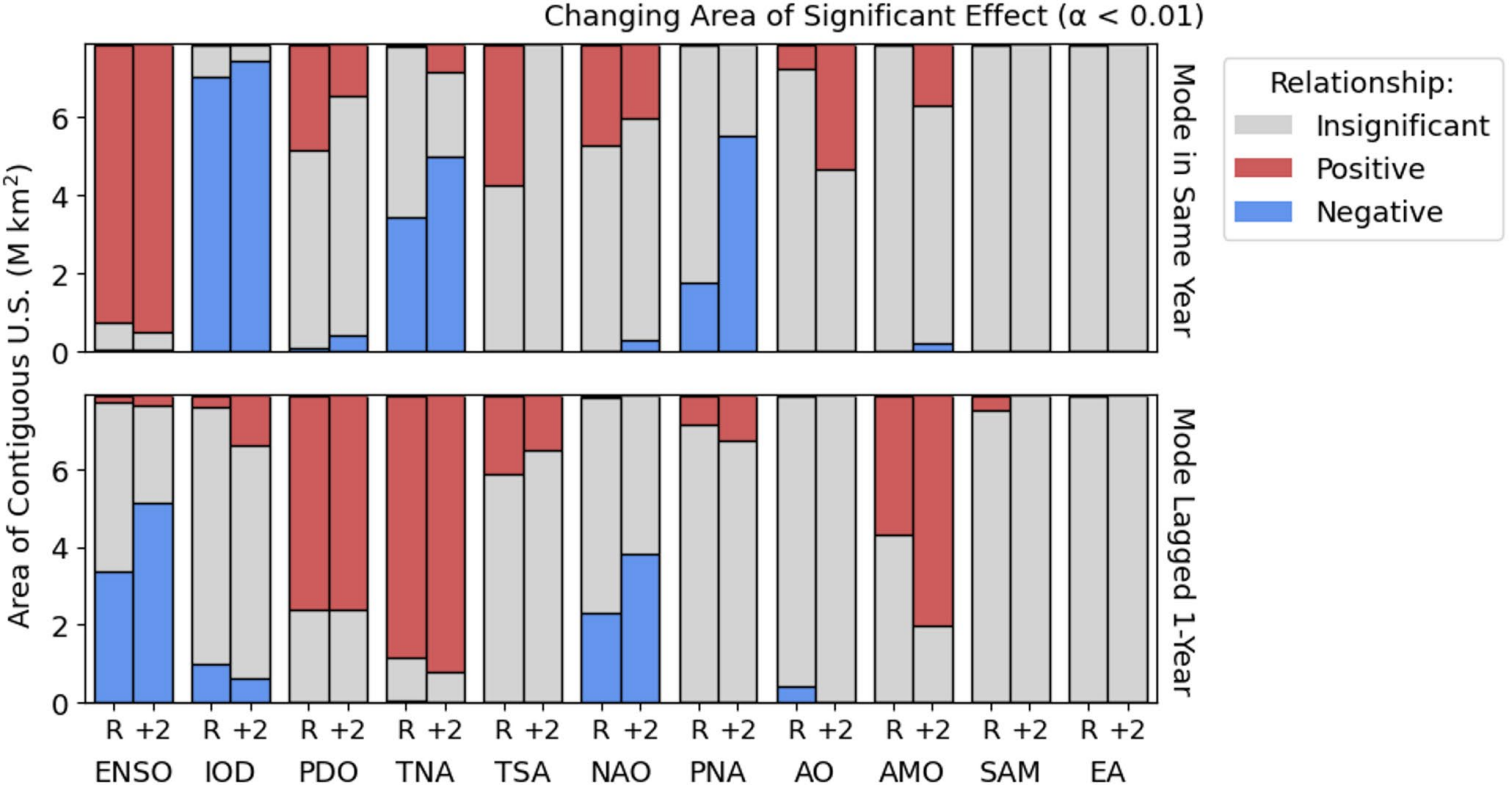
Difference in the significant areal influence of each climate mode in the same year and with a one-year lag between the recent ensemble (R) and the + 2°C ensemble (+ 2). The areas are given in Supplementary Sect. 11. If the colour is the same in both R and + 2, then the effect of the mode in that area stays the same; if the colour changes, this indicates that the effect of the mode in that area changes between the two ensemble climates

An additional 2°C increase in global mean temperature in KNMI-LENTIS results in changes to the areal extent of the significant influence of many of the climate modes on wildfire occurrences (Fig. 9). There were only a few cases when the influence of a mode on wildfire changed from positive to negative, but cases where the impact of a mode changed from insignificant to significant were widespread. The AMO+1 and PNA show the greatest expansion in the area of significant effect, increasing from 38 to 68% and 16 to 56% respectively (Supplementary Sect. 11). Some other modes show a persistent influence in the areas they affect under recent conditions with only a slight expansion: ENSO (89 to 92%), the IOD (87 to 92%), and the TNA+1 (82 to 87%). There is also a significant expansion in the area of significant influence on wildfire for the ENSO+1, TNA, NAO+1, AMO and AO indices. However, the TSA has no area of significant influence in the future climate, and the area of influence of the NAO shifts from the western to the central US (Supplementary Sect. 11).

There is a minor but statistically significant shift in the skewness of the ENSO distribution in response to warming (Supplementary Fig. 5.15). However, comparing the effect on annual fires of the unaltered ENSO in the future climate, and of the future distribution mapped to percentiles in the recent climate revealed no substantial change in the effect of ENSO on wildfire occurrences due to this shift. This suggests that ENSO’s projected effect on fire is mainly due to an increase in the intensity of ENSO’s effect on rainfall (Figs. 6 and 11) over North America, in line with consensus expectations (IPCC 2021).

**Fig. 10:**
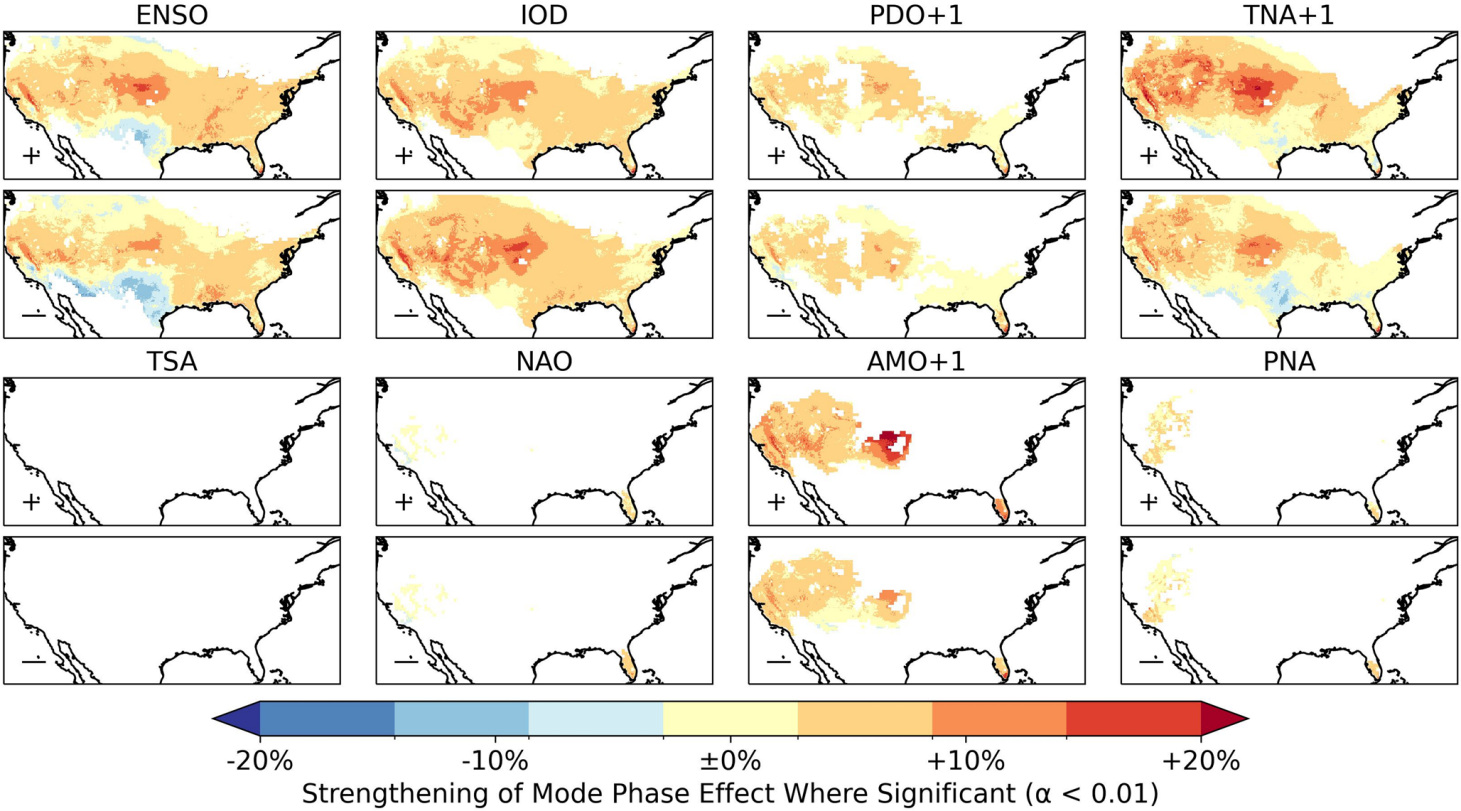
The strengthening or weakening of the effect of each climate mode in both phases between the recent climate and + 2°C climate time-slices. Each panel shows the ratio between recent climate and the + 2°C climate for the number of annual wildfires in that phase of the global climate mode relative to the mean at that location. Only regions where the effect of the mode is significant at α < 0.01 according to linear regression in both time-slices are shown

Despite some climate modes being substantial controls on the internal variability in annual fire occurrences, the effect of—for example—La Niña in the present climate is significantly less than the effect of a further + 2°C global warming (Supplementary Fig. 11.10). However, in the future climate the effect of La Niña relative to the higher rate of fire activity is nonetheless projected to increase. The change in strength of the relationship between modes and the annual number of wildfires varies geographically (Fig. 10). The AMO+1 and TNA+1 show the most substantial increase in the effect of their positive phase on the annual number of wildfires; both strengthen most over the Great Plains and in the West. The positive TNA+1 and La Niña both show a weakening in their influence on annual wildfire occurrences along the southern border with Mexico, most substantially in Texas. In contrast, the negative phase of the IOD has a strengthened effect on wildfire occurrences along the southern border, strongest in Arizona and New Mexico.

The PDO+1 strengthens in its association with wildfire occurrences across its area of significant influence, with the strengthening most over the Great Plains and in California. The influence on the annual number of wildfires relative to the mean, and the area of influence of the AO and PNA (Supplementary Sect. 11) significantly increase, with the greatest relative increase over the Great Plains. The AMO+1, TNA+1, PNA, PDO+1, IOD, ENSO and AO modes all strengthen in their influence over the majority of the region they affected in the recent climate. As the effect of the modes in the + 2°C time-slice is calculated relative to a higher mean rate of wildfire in the + 2°C climate, this means the control of these global climate modes on wildfire increases even relative to the higher future interannual variability of wildfire.

**Fig. 11:**
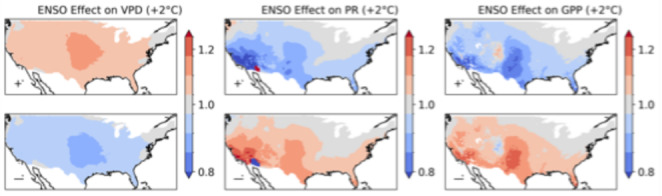
the relative influence of ENSO on VPD, precipitation and GPP in the + 2°C climate for the large ensemble

Climate change drives a strong intensification of the relative effect of ENSO on VPD over the Great Plains (Fig. 11)—corresponding to where the greatest intensification of ENSO is also modelled (Fig. 10). The effect of ENSO on southwestern US precipitation is also projected to intensify with climate change, however the association with wildfire decreases in the same region. This could be explained by the diminishing effect of annual-timescale variability dryness on wildfire danger in an increasingly arid and fuel-limited environment (Abatzoglou and Williams 2016).

## Discussion

Our analyses have shown that ENSO, IOD and TNA+1 are the global climate modes most associated with interannual variability in wildfire occurrence in the contiguous US. ENSO has a significant influence over 91% of the country, consistent with its use in predicting wildfire severity across the US (NIFC, 2024a; b). La Niña strongly increases annual wildfire probability over California, the interior southwestern US, the Great Plains, and southern Florida. La Niña years have been associated with increased wildfire activity in the southwestern US due to reduced precipitation (Margolis and Swetnam 2013; Mason et al. 2017; Westerling and Swetnam 2003), as well as in the southeastern states of Mississippi (Dixon et al. 2008) and Florida (Goodrick and Hanley 2009). La Niña has also been associated with severe wildfires in the southern Great Plains (Lindley et al. 2014). However, we only identify a moderate effect of La Niña on wildfire occurrence for most of the southeastern US, with a highly localised effect over southern Florida. This could be explained by the strength of the effect of ENSO on precipitation (and consequent higher GPP) in the southwestern US and Florida, compared to a lesser effect over the wider southeastern US. We also identify a well-defined hotspot for the effect of La Niña on wildfire in the Great Plains, where ENSO has been linked qualitatively to wildfires (Lindley et al. 2014). VPD shows a strong response to ENSO over the Great Plains, with the increased hot, dry conditions in La Niña years therefore a likely primary contributor to the enhanced wildfire likelihood. The effect of La Niña relative to the mean wildfire rate intensifies with climate change over the Great Plains and California.

The negative IOD and positive TNA+1 show a very similar pattern of effect to La Niña when considered independently, with the TNA+1 having a greater effect. The TNA+1 and IOD are causally linked (Wang et al. 2021; Ham et al. 2013; Hameed et al. 2018; Jiang and Li 2019) and correlated with ENSO; and the similarity of their influence on wildfire raises questions about the extent to which they are simply proxies for the phase of ENSO. Our MLR analyses show that while ENSO has the greatest influence on wildfire variability, TNA+1 also has an independent though smaller effect while the IOD has only a limited impact in the region near the US-Mexico border. The correlation of the TNA+1 and the IOD with ENSO preclude a definite attribution of the causal influence of each mode on wildfire occurrence. However, our analyses suggest that the strongest apparent pathway of influence is via ENSO and TNA+1. Although the TNA and IOD have been closely associated with burnt area at a global scale (Cardil et al. 2023), their impact of wildfires in the US has been largely ignored.

ENSO creates a moisture dipole between the northwestern and southwestern US through influencing the latitude of the jet stream (Dettinger et al. 1998; Westerling and Swetnam 2003). It has therefore been suggested that ENSO increases wildfire activity in the northwestern US in El Niño years (Barbero et al. 2015; Johnston et al. 2017). However, there is almost no statistically significant influence in this region in our simulation of 1600 years of annual wildfire occurrences in the recent and + 2°C climates, with the positive effect of El Niño on northwestern US wildfire only statistically significant over a small region. There is a possibility that this is due to an unknown bias in EC-Earth3’s representation of ENSO over the region, however assessment of the mode’s behaviour and effect over the US does not reveal any obvious issues (Supplementary Sect. 5, Döscher et al. 2022)—with the moisture dipole represented in the model. Alternatively, this could be explained by most analysis in this region having focussed on fire frequency or burnt area. El Niño has a greater effect on fire size (Heyerdahl et al. 2002; Barbero et al. 2015), a distinct property from occurrence likelihood, which could be sufficient to explain the greater rates of fire frequency for El Niño years in the tree ring record. Links between wildfire occurrence and the PDO+ (Ascoli et al. 2020; Heyerdahl et al. 2002; Norman and Taylor 2003; Schoennagel et al. 2005) and AMO+ (Ascoli et al. 2020; and Kitzberger et al. 2007) in the northwestern US are also not significant in our analyses.

The AMO+1 is an important control on wildfire likelihood over 38% of the contiguous US under recent conditions, particularly in the West and southern Florida. The impact of the AMO on wildfires in the + 2 °C time-slice increases considerably in the central US and along the East Coast, with a > 25% increase in annual wildfire occurrences in the Great Plains and southern Florida.; the strengthened effect of AMO+1 on US wildfire occurrence indicates that the positive relationship between this mode and wildfire also applies to higher amplitudes of the oscillation. The AMO+1 and TNA+1 show the greatest strengthening in their effect with future warming, indicating that high Atlantic SSTs in the previous year are strongly associated with interannual wildfire variability under climate warming. The AMO+ has been linked to wildfire activity in the same year (Ascoli et al. 2020; and Kitzberger et al. 2007), but our analysis shows no significant effect in the recent climate and only a minor area of influence with warming. This might seem contradictory to its multidecadal effect, but when no low-pass filtering is applied to the AMO, annual oscillations between neutral and high positive or negative values occur in its multidecadal positive or negative phases respectively (Sutton and Hodson 2007). Atlantic SSTs in the prior year have a greater effect on US wildfire than in the same year.

The Great Plains is the region where wildfires show the most sensitivity to variation in climate modes. There is a > 25% increase in annual wildfire occurrences under recent conditions with La Niña, the positive TNA+1 and the negative IOD. Wildfire is also increased to a similar extent by the positive AMO+1 in the + 2°C time-slice. The PDO+1, PNA and AO, all anticipated to change with warming (Litzow et al. 2020; Ning and Bradley 2016; Zhang et al. 2016; Choi et al. 2010), also have a significant impact on wildfire probability in this region. The sensitivity of the Great Plains to climate variability is in part due to meteorological effects on fire weather but also reflects the importance of vegetation type. Grassland and savanna vegetation have a strong response to climate variability: higher-than-normal antecedent precipitation increases fuel production and short-term droughts cause rapid drying of grassy vegetation (Littell et al. 2009; Archibald et al. 2009). The overall abundance of the vegetation may also contribute to this sensitivity. In the interior western US, for example, temperate sierras and forested mountains have a higher sensitivity to climate variability than the surrounding desert.

The climate mode indices were derived according to standard methods. The modes of variability in climate models might differ from those in the observational record. However, all the modes considered here are represented in the recent and + 2°C ensembles, and show reasonable geographic patterns in terms of the associated SST or SLP anomalies and the expected precipitation anomalies over the contiguous US. The ensemble climate modes can therefore be considered phenomenologically similar to observations, and thus to have the same causal effects on wildfire likelihood. The periodicity of multi-year oscillations cannot be assessed from the decadal time-slices. However, the transient EC-Earth3 runs from which the time-slices were sourced show the expected periodicity for each mode, including the longer periodicities of the PDO and AMO. The PDO has much higher interannual variability than the AMO, resulting in a tendency for sub-decadal oscillations within a time slice. Whilst a low-pass filtering of the PDO (and AMO) time-series might provide a better representation of decadal to multidecadal effects, both the SST and the meteorological patterns associated with these modes was correct.

Climate modes are often correlated and can modulate the effect of other climate modes. This means that the association between a single climate mode and wildfire can also embed information on other climate modes that may be more directly causally linked to wildfire activity, as shown by the analysis of the TNA, IOD and ENSO. The impact of a particular mode on wildfires can be established through consideration of the mean effect in different phases, as shown for ENSO in the southwestern US. However, there may be mechanisms that disrupt the expected patterns of climate variability associated with a particular mode. One example of this is the impact of years with exceptional atmospheric river activity over the southwestern US which led to a reversal of the expected precipitation patterns associated with La Niña and El Niño (Luna-Niño et al. 2025).

We used commonly used metrics (Jolly et al. 2015; Abatzoglou et al. 2019) to characterise the peak timing and length of the fire season. These were defined as the month with the most fires and any months with over half the annual maximum month respectively. However, it is difficult to characterise the seasonal peak in this way when there is a similar likelihood of wildfires across most of the year and this approach also does not resolve the effect of climate modes on bimodal fire seasons, such as in the Appalachian Mountains (Lafon et al. 2005). Defining the length of the fire season relative to the average value of the highest fire month at a given location is appropriate for local comparisons but makes it difficult to compare changes across regions with very different baseline fire regimes. The similar increase in the lengthening of the fire season in the Great Plains and Mediterranean California, for example, will have different consequences given that the occurrence of wildfires is lower in the Great Plains.

We used a wildfire probability model to link climate modes to annual wildfire occurrences. The model represents the probability of wildfire events over a 0.1-hectare threshold, but does not simulate other wildfire attributes such as size or intensity. Thus, the impact of changes in climate modes on wind patterns and storm tracks that can lead to extreme wildfires are not accounted for in this study. In addition to the climate drivers of wildfire probability, we included the impact of climate-driven changes in GPP on wildfires. However, other factors that influence the likelihood of wildfire occurrence such as fuel removal, previous wildfires or lightning ignitions are not taken into account. Some studies have identified different responses to climate depending on environment—for example a difference in response to seasonal VPD in forested and non-forested ecosystems in California (Williams et al. 2019). However, such difference in response can be explained by other environmental variables—for example the key limitation of fuel production on wildfire in dry, southwestern US ecosystems. Lightning was also not accounted for in the final occurrence model, due to the poor predictability of lightning from climate variables. Furthermore, while the wildfire probability model also includes predictors related to human activity, these were held constant in our analyses. Despite these limitations, the wildfire occurrence model used has good predictive capability (Keeping et al. 2024), and is responsive to trends and variability in meteorological drivers of wildfires whilst also accounting for other human and vegetation related effects.

This paper presents strong evidence for the utility of using multiple climate modes to supplement long-range fire season forecasts, with high utility from a risk management perspective. Recent progress in seasonal fire weather forecasting (Di Giuseppe et al., 2024) means that fire weather anomalies can be predicted up to 1 month in advance globally. However, predictable modes provide additional information on the likely wildfire season—giving enough time for risk management interventions such as fuel removal and prescribed burning. Such correlations have already been found using observed data in a number of regional and local studies (Chen et al. 2011, 2016; Fernandes et al. 2011; Shen et al. 2019; Cardil et al. 2023). This is now supplemented in the US by modelled evidence to a considerably higher level of statistical significance.

## Conclusions

Large wildfire ensembles allow the identification of high-resolution geographical patterns of the impact of climate modes on wildfire. We have identified the areal extent of the influence of different modes on wildfire for the contiguous US, their impact on the timing of the fire season, and how the relative effect on annual wildfires varies geographically under recent and future climates. ENSO, the IOD, and TNA+1 are found to be the principal climate modes associated with US wildfire variability under recent conditions, affecting 91%, 90% and 82% of the contiguous US. La Niña increases the likelihood of wildfire in the southwestern US, Great Plains and Florida. The IOD and TNA+1 have a more limited effect on US wildfire when accounting for the simultaneous effect of ENSO. Nonetheless, the TNA+1 remains a strong and widespread influence over the contiguous US in the recent and + 2°C climates, and the IOD emerges as a substantial regional control in the southern US with future warming. Contrary to expectations, no significant relationship was found between El Niño and wildfire occurrences in the northwestern US. The strong association of ENSO and its related modes on annual expected fires can be explained by ENSO’s strong effect on heat (VPD) and precipitation (which contributes to GPP variability). ENSO has a very strong effect on precipitation in the southwest, with a statistically significant effect on precipitation across the entire southern US. ENSO also has a broad effect on VPD in most of the US, strongest in the Great Plains.

An additional + 2°C global warming strengthens the effect of some climate modes on wildfire occurrence, particularly that of the TNA+1 and AMO+1 on wildfire in the Great Plains and the West. The effect comes primarily from changes in the impact of climate modes in a warmer climate, rather than from changes in the modes themselves. The Great Plains is the region where wildfire probability is most sensitive to climate change and variability; wildfire in the Great Plains is strongly controlled by ENSO, the IOD and TNA+1 in the recent and + 2°C time-slices, and additionally by AMO+1 in the + 2°C time-slice. Over the contiguous US, the number of wildfires and the areal extent of influence of the PNA, AO and PDO+1 also increase with warming. The area significantly influenced by the PNA increased from 26 to 66% and by the AO from 10 to 37%.

## Electronic Supplementary Material

Below is the link to the electronic supplementary material.

Supplementary Material 1

## Data Availability

The source datasets used this study can be found in the KNMI-LENTIS repository 10.5281/zenodo.7573137.

## Code availability

The code used to generate and analyse new data for this study can be found in the reposity or 10.5281/zenodo.17900607.
