# Supplementary material for: Influence of global climate modes on wildfire occurrence in the contiguous United States under recent and future climates
Source: Clim Dyn. 2025 Dec 19;64(1):15. doi: 10.1007/s00382-025-07998-w (PMC12717122; doi:10.1007/s00382-025-07998-w)
Supplement: Supplementary file 1 — Supplementary Material 1 [file 382_2025_7998_MOESM1_ESM.docx]

## **Supplementary Material: Influence of climate modes on wildfire occurrence in the contiguous United States under current and future climates**

Theodore R. Keeping*^1,2^, Theodore G. Shepherd^3^, I. Colin Prentice^4^, Karin van der Wiel^5^, Sandy P. Harrison^1,2^

^1^Geography & Environmental Science, University of Reading, Reading, UK

^2^Leverhulme Centre for Wildfires, Environment and Society, Imperial College London, London, UK

^3^Department of Meteorology, University of Reading, Reading, UK

^4^Georgina Mace Centre for the Living Planet, Department of Life Sciences, Imperial College London, Ascot, UK

^5^Royal Netherlands Meteorological Institute (KNMI), De Bilt, The Netherlands

*** Correspondence:**

Theodore R. Keeping

[t.r.keeping@pgr.reading.ac.uk](mailto:t.r.keeping@pgr.reading.ac.uk)

The Supplementary Material is divided into eight sections. **Section 1** gives an overview of previous work linking climate modes and wildfire in the contiguous US. **Section 2** evidences the insufficiency of reanalysis data to derive climate mode/wildfire relationships. **Section 3** provides maps of the ensemble mean and spread as well as regions referred to in this study. **Section 4** provides an evaluation of the performance of the bias correction for the KNMI-LENTIS ensemble. **Section 5** provides an assessment of the representation of climate modes in the KNMI-LENTIS ensemble. **Section 6** provides additional figures showing the areal effect of individual climate models in the recent climate ensemble. **Section 7** provides the ensemble correlations between ENSO, the IOD and TNA+1 indices in the recent and +2°C climate ensembles. **Section 8** gives maps of the effect of climate modes on key predictors of wildfire occurrence. **Section 9** provides information of the effects of climate models on the probability distribution of annual wildfire numbers in different regions in the recent ensemble climate**. Section 10** illustrates the effect of climate modes on the wildfire seasonal length and peak timing in the recent ensemble climate. **Section 11** provides additional material on the areal extent and magnitude of the impact of climate modes in the +2°C ensemble.

The Supplementary contains the following figures and tables:

**Table 1.1** Overview of previous studies of the relationships between wildfire and climate modes

**Figure 2.1** The association between reanalysis (1990-2019) annual wildfires and climate modes to 0.05 FDR-corrected significance.

**Figure 2.2** The association between reanalysis (1990-2019) annual wildfires and climate modes to any level of significance.

**Figure 2.3** The association between observed (FPA FOD) (1992-2019) annual wildfires and climate modes to 0.05 FDR-corrected significance.

**Figure 2.4** The association between observed (FPA FOD) (1992-2019) annual wildfires and climate modes to any level significance.

**Figure 2.5** Box plots of randomly selected mean annual wildfires compared to the effect seen in the large ensemble.

**Figure 3.1** Maps of mean observed wildfire occurrences (FPA FOD), mean reanalysis modelled occurrences, and the mean and spread of annual wildfire occurrences in the ensemble recent and future climate.

**Figure 3.2** The regions referred to in this study, both ecoregions and GACC admin regions.

**Table 4.1** Performance statistics of input variables into wildfire occurrence model. R^2^ statistics comparing reanalysis data to the bias corrected and downscaled KNMI-LENTIS data input into the wildfire occurrence model, for the top four statistical moments.

**Figure 5.1** The SST effect associated with the Atlantic Multidecadal Oscillation (AMO).

**Figure 5.2** The SST effect associated with the Tropical South Atlantic (TSA).

**Figure 5.3** The SST effect associated with the Tropical North Atlantic (TNA).

**Figure 5.4** The PSL effect associated with the Southern Annular Mode (SAM).

**Figure 5.5** The PSL effect associated with the Pacific/North American (PNA) Oscillation.

**Figure 5.6** The SST effect associated with the Pacific Decadal Oscillation (PDO).

**Figure 5.7** The SST effect associated with the North Atlantic Oscillation (NAO).

**Figure 5.8** The SST effect associated with the Indian Ocean Dipole (IOD).

**Figure 5.9** The SST effect associated with El Niño Southern Oscillation (ENSO).

**Figure 5.10** The PSL effect associated with the East Atlantic (EA) Oscillation.

**Figure 5.11** The PSL effect associated with the Arctic Oscillation (AO).

**Figure 5.12** The effect of all investigated modes on annual precipitation over the contiguous US.

**Figure 5.13** The AMO index calculated from the 16 transient runs (1950-2100) from which the 160 time-slice ensemble members are derived.

**Figure 5.14** The PDO index calculated from the 16 transient runs (1950-2100) from which the 160 time-slice ensemble members are derived.

**Figure 5.15** The distribution of the mode values in the recent and future climates, with K-S test p-values comparing the distributions between time-slices given.

**Figure 5.16** The SST effect associated with El Niño Southern Oscillation (ENSO) in the recent and future climates for comparison.

**Figure 6.1** Scatterplots showing the relationship between ENSO and IOD, and ENSO and TNA+1 in the recent and +2°C climates

**Figure 6.2** Scatterplots of annual temperature and precipitation in the contiguous US against the climate mode index values.

**Figure 7.1** The area of effect of each mode to a 5-sigma significance threshold.

**Figure 7.2** The seasonally discrete area of significant influence of each mode.

**Figure 7.3** The geospatial effect on the annual number of fires of all modes not given in Figure 2.

**Figure 8.1** The effect of climate modes on annual precipitation over the contiguous US in the recent climate.

**Figure 8.2** The effect of climate modes on annual vapour pressure deficit over the contiguous US in the recent climate.

**Figure 8.3** The effect of climate modes on annual gross primary productivity over the contiguous US in the recent climate.

**Figure 8.4** The effect of climate modes on annual precipitation over the contiguous US in the +2°C climate.

**Figure 8.5** The effect of climate modes on annual vapour pressure deficit over the contiguous US in the +2°C climate.

**Figure 8.6** The effect of climate modes on annual gross primary productivity over the contiguous US in the +2°C climate.

**Figure 9.1** The effect of the positive and negative phases of the AMO+1 on the annual wildfire distributions in each of the US Geographic Area Coordination Centres.

**Figure 9.2** The effect of the positive and negative phases of the TSA on the annual wildfire distributions in each of the US Geographic Area Coordination Centres.

**Figure 9.3** The effect of the positive and negative phases of the TNA+1 on the annual wildfire distributions in each of the US Geographic Area Coordination Centres.

**Figure 9.4** The effect of the positive and negative phases of the PNA on the annual wildfire distributions in each of the US Geographic Area Coordination Centres.

**Figure 9.5** The effect of the positive and negative phases of the PDO+1 on the annual wildfire distributions in each of the US Geographic Area Coordination Centres.

**Figure 9.6** The effect of the positive and negative phases of the NAO on the annual wildfire distributions in each of the US Geographic Area Coordination Centres.

**Figure 9.7** The effect of the positive and negative phases of the IOD on the annual wildfire distributions in each of the US Geographic Area Coordination Centres.

**Figure 9.8** The effect of the positive and negative phases of the ENSO on the annual wildfire distributions in each of the US Geographic Area Coordination Centres.

**Figure 10.1** The effect of the positive and negative phases of ENSO on the length and peak timing of the fire season over the contiguous US.

**Figure 10.2** The effect of the positive and negative phases of IOD on the length and peak timing of the fire season over the contiguous US.

**Figure 10.3** The effect of the positive and negative phases of TNA+1 on the length and peak timing of the fire season over the contiguous US.

**Figure 10.4** The effect of the positive and negative phases of PDO+1 on the length and peak timing of the fire season over the contiguous US.

**Figure 10.5** The effect of the positive and negative phases of TSA on the length and peak timing of the fire season over the contiguous US.

**Figure 10.6** The effect of the positive and negative phases of NAO on the length and peak timing of the fire season over the contiguous US.

**Figure 10.7** The effect of the positive and negative phases of AMO+1 on the length and peak timing of the fire season over the contiguous US.

**Figure 10.8** The effect of the positive and negative phases of PNA on the length and peak timing of the fire season over the contiguous US.

**Table 11.1** The changing areas of the contiguous US affected by each mode with a future +2°C warming.

**Figure 11.1** The geospatial effect of the AMO+1 on the annual number of wildfire occurrences in the recent and +2°C time-slices, with the strengthening or weakening effect of the mode (relative to the mean rate of wildfire occurrence) with +2°C climate change also shown.

**Figure 11.2** The geospatial effect of the TNA+1 on the annual number of wildfire occurrences in the recent and +2°C time-slices, with the strengthening or weakening effect of the mode (relative to the mean rate of wildfire occurrence) with +2°C climate change also shown.

**Figure 11.3** The geospatial effect of the TSA on the annual number of wildfire occurrences in the recent and +2°C time-slices, with the strengthening or weakening effect of the mode (relative to the mean rate of wildfire occurrence) with +2°C climate change also shown.

**Figure 11.4** The geospatial effect of the PNA on the annual number of wildfire occurrences in the recent and +2°C time-slices, with the strengthening or weakening effect of the mode (relative to the mean rate of wildfire occurrence) with +2°C climate change also shown.

**Figure 11.5** The geospatial effect of the PDO+1 on the annual number of wildfire occurrences in the recent and +2°C time-slices, with the strengthening or weakening effect of the mode (relative to the mean rate of wildfire occurrence) with +2°C climate change also shown.

**Figure 11.6** The geospatial effect of the NAO on the annual number of wildfire occurrences in the recent and +2°C time-slices, with the strengthening or weakening effect of the mode (relative to the mean rate of wildfire occurrence) with +2°C climate change also shown.

**Figure 11.7** The geospatial effect of the IOD on the annual number of wildfire occurrences in the recent and +2°C time-slices, with the strengthening or weakening effect of the mode (relative to the mean rate of wildfire occurrence) with +2°C climate change also shown.

**Figure 11.8** The geospatial effect of the ENSO on the annual number of wildfire occurrences in the recent and +2°C time-slices, with the strengthening or weakening effect of the mode (relative to the mean rate of wildfire occurrence) with +2°C climate change also shown.

**Figure 11.9** The geospatial effect of the AO on the annual number of wildfire occurrences in the recent and +2°C time-slices, with the strengthening or weakening effect of the mode (relative to the mean rate of wildfire occurrence) with +2°C climate change also shown.

**Figure 11.10** Comparative effect of La Niña years versus +2°C global warming on wildfire occurrence likelihood.

### **Supplementary Section 1: Overview of Previous Studies**

Most previous studies on the association between climate modes and wildfire in the contiguous US have focussed on site-based or regional reconstructions of the wildfire record from tree ring fire-scars. These can provide analyses covering multiple decades to centuries, but necessarily only focus on a limited spatial extent. State wildfire records and remote sensing have also been used. These cover a larger and more continuous area, but cover a shorter study period where the effects of a given climate mode may be much more uncertain.

Acronyms: ENSO (El Niño Southern Oscillation), AMO (Atlantic Multidecadal Oscillation), PDO (Pacific Decadal Oscillation), SOI (Southern Oscillation Index), NAO (North Atlantic Oscillation), PDSI (Palmer Drought Severity Index), MEI (Multivariate ENSO Index), NOAA (National Oceanic and Atmospheric Administration), NCAR (National Center for Atmospheric Research), NCEP (National Centers for Environmental Prediction), PNA (Pacific/North American).

*Supplementary Table 1.1: Overview of previous studies of the relationships between wildfire and climate modes for the contiguous US.*

| **Reference** | **Study Extent** | **Study Period** | **Mode Source** | **Data Source** | **Wildfire Property** |
| --- | --- | --- | --- | --- | --- |
| Ascoli et al. (2020) | Alaska, Yukon, Alberta, Quebec. (7, 8, 15, 7 sites) | 1957–2016, 1987–2016, 1951–2016, 1989–2014 | ENSO and AMO from ocean temperatures. | Correlation of *picea glauca* masting chronologies with burnt area | Burnt area |
| Westerling & Swetnam (2003) | Continental United States (number of trees and number of sites not stated) | 1701–1978, 1916–1978 | ENSO and PDO reconstructed (tree-ring) and modern (observed) | Fire scars | Fire occurrence |
| Hessl et al. (2004) | Central and Eastern Washington State (5 sites, 1701 trees) | 1700-1990 | ENSO and PDO from tree rings |  |  |
| Heyerdahl et al. (2002) | Eastern Oregon and Washington States (4 sites, 3659 trees) | 1687–1994 | ENSO from SOI (modern) and tree rings (historical) |  |  |
| Heyerdahl et al. (2008) | Interior Oregon and Washington States and Southern British Columbia Province (15 sites, 3720 trees) | 1651–1900 | ENSO and PDO from tree rings |  |  |
| Johnston et al. (2017) | East Oregon (13 sites, 189–201 trees) | 1650–1900 | ENSO and PDO from published reconstructions (tree rings and reanalysis meteorology) |  |  |
| Kipfmueller et al. (2012) | Pacific Northwest (Oregon, Washington, Idaho, Montana, Wyoming), Central Rockies (Colorado Wyoming), Southwest (Colorado, New Mexico, Arizona) (128 sites) | 1700–1900 | ENSO and PDO from tree rings |  |  |
| Kitzberger et al. (2007) | Sierra Madre Occidental, Arizona, Southern New Mexico, Northern New Mexico, Southern Colorado, Northern Colorado, Black Hills (South Dakota), Sierra Nevada (California), Blue Mountains (Oregon, Washington Southern British Columbia, Canada) (238 sites, 4760 trees) | 1550–1924 | ENSO, PDO and AMO from tree rings |  |  |
| Le Goff et al. (2007) | Waswanipi (Quebec) (46 trees, 31 sites) | 1720–2000 | PDO, NAO, AMO from tree rings, post-reformation European weather data |  |  |
| Margolis & Swetnam (2013) | Southwestern US (Utah, Colorado, Arizona, New Mexico) (n trees not stated, 16 sites) | 1700-1904 (and 1905-1978 for PDSI) | ENSO, PDO and AMO from tree rings |  |  |
| Moody et al. (2006) | Plumas National Forest (California) (144 trees, 4 sites) | 1454–2001 | ENSO and PDO from tree rings |  |  |
| Norm & Taylor (2003) | Lassen National Forest (California) (112 trees, 8 sites) | 1700–1849 | ENSO and PDO, from pre-1900 tree rings |  |  |
| Schoennagel et al. (2005) | Jasper National Park (Northern Rockies), Yellowstone National Park (Central Rockies), Rocky Mountain National Park (Southern Rockies) (1694 trees, 3 study areas) | 1700– 1975 | ENSO and PDO tree ring reconstructions |  |  |
| Sibold & Veblen (2006) | Rocky Mountain National Park (Colorado) (6152 trees, 487 sites) | 1650–1978 | ENSO, PDO and AMO tree ring reconstructions |  |  |
| Trouet et al. (2010) | Pacific Northwest, Northern California, Interior West, Southwest (2980 trees, 350 sites) | 1441–1910, 1400–1861, 1281–1974, 1403–1859 | ENSO tree ring reconstruction |  |  |
| Kitzberger et al. (2001) | Southwestern United States (Arizona and New Mexico) (63 sites, 933 trees) | 1914–1987 | ENSO from tree rings | Fire scars and US federal burnt area records | Fire occurrence and burnt area |
| Swetnam & Betancourt (1998) | Arizona, New Mexico, Sonora (>900 trees, 63 sites) | 1700–1900,  1920–1978 | ENSO reconstruction (provenance unknown) |  |  |
| Swetnam & Betancourt (1990) | Arizona, New Mexico (n trees not stated, 28 sites) | 1700–1905, 1905–1985 | ENSO tree ring reconstruction |  |  |
| Barbero et al. (2015) | Northwestern Ecoregions (Northern Rockies, Canadian Rockies, Middle Rockies, Idaho Batholith, Northern Basin and Range, Central Basin and Range, and Snake River Plain) | 1984–2012 | ENSO from reanalysis (MEI) | National/ state fire records | Very large fires |
| Dixon et al. (2008) | Mississippi State | 1990–2006 | ENSO, NAO, PNA, PDO NOAA modes |  | Fire occurrence and size |
| Simard et al. (1985) | Continental United States | 1926–1978 | Strong ENSO years from 9 common indicators |  | Fire occurrence and burnt area |
| Cardil et al. (2021) | Southern Coastal California Ecoregion | 1953–2018 | NOAA modes |  | Burnt area |
| Goodrick & Hanley (2009) | Florida State | 1981–2003 | PNA and NAO from NOAA |  |  |
| Fauria & Johnson (2008) | Canada | 1918–2005 | ENSO, PDO, AO from NCAR/NOAA |  |  |
| Cardil et al. (2023) | Global | 1982–2018 | NOAA modes | Remote sensing | Burnt area |
| Justino et al. (2022) | North of 40°N | 2001–2020 | AO and PNA from reanalysis |  | Fire occurrence |
| Mason et al. (2017) | Continental US | 1979–2015 | Derived from NCEP NARR | Reanalysis risk index | Fire danger |

### **Supplementary Section 2: Viability of Reanalysis to Find Mode Effects**


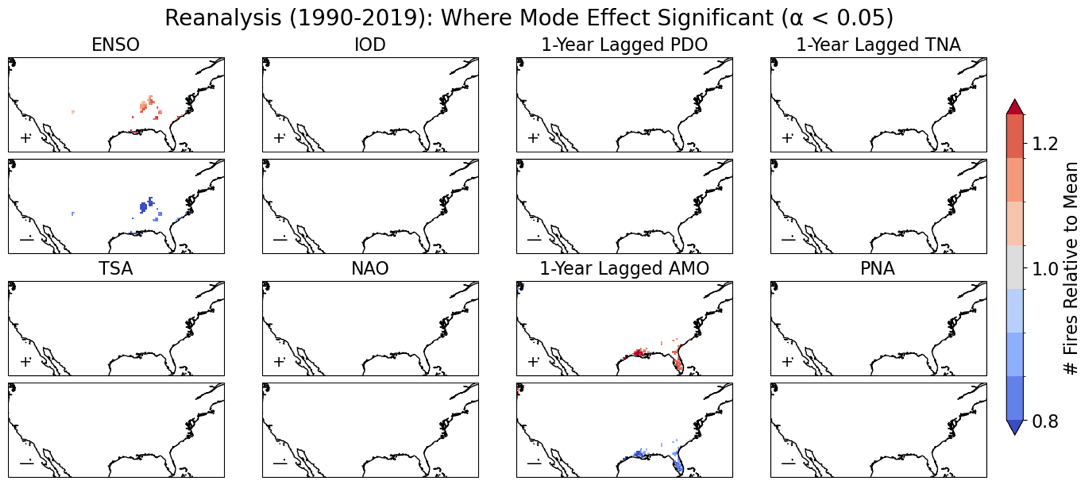


*Supplementary Figure 2.1: the association between the reanalysis fire model and key climate modes where there is an FDR-corrected statistical significance level of 0.05. The sign and magnitude of the relationship is given by the ratio between the annual number of wildfires in the positive (upper panels) or negative (lower panels) phase – defined as beyond plus or minus half a standard deviation from the mean respectively. The effect of each mode is shown relative to the mean annual number of wildfires – to account for any non-linearity in the effect between phases that would not be captured by linear regression.*


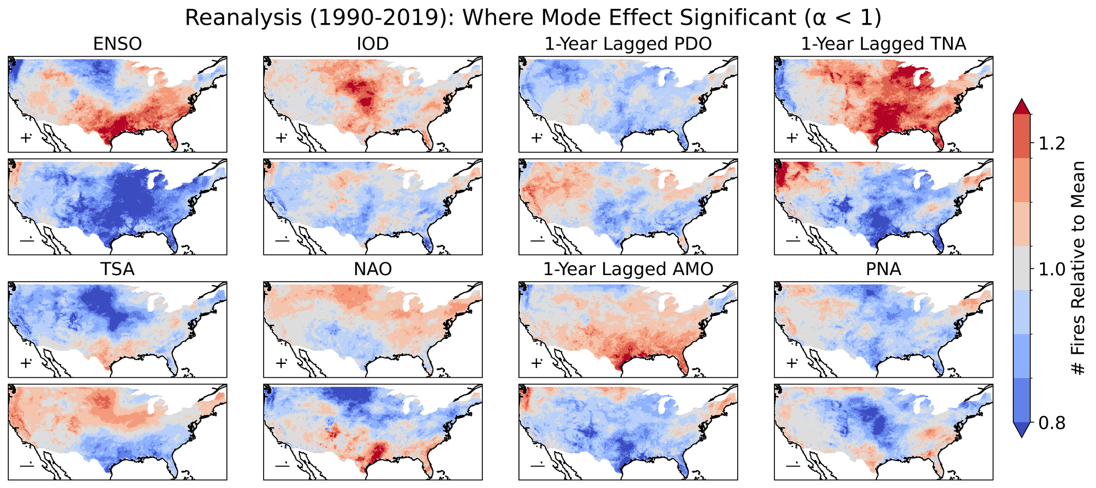


*Supplementary Figure 2.2: the association between the reanalysis fire model and key climate modes with no level of statistical significance prescribed. The sign and magnitude of the relationship is given by the ratio between the annual number of wildfires in the positive (upper panels) or negative (lower panels) phase – defined as beyond plus or minus half a standard deviation from the mean respectively. The effect of each mode is shown relative to the mean annual number of wildfires – to account for any non-linearity in the effect between phases that would not be captured by linear regression.*


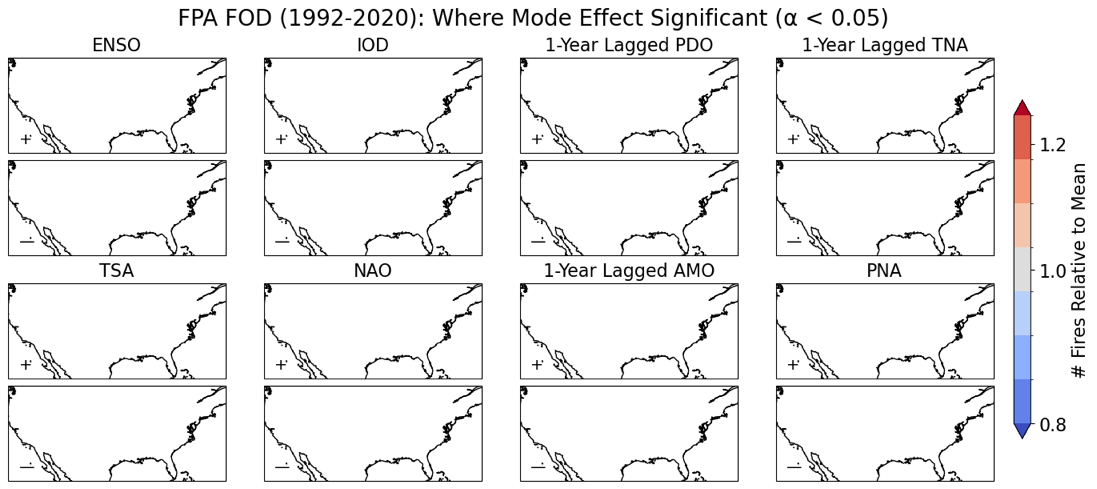


*Supplementary Figure 2.3: the association between the FPA FOD fire occurrence data and key climate modes to an FDR-corrected significance of 0.05. The sign and magnitude of the relationship is given by the ratio between the annual number of wildfires in the positive (upper panels) or negative (lower panels) phase – defined as beyond plus or minus half a standard deviation from the mean respectively. The effect of each mode is shown relative to the mean annual number of wildfires – to account for any non-linearity in the effect between phases that would not be captured by linear regression. Note that the FPA FOD starts at 1992, hence the curtailed time period.*


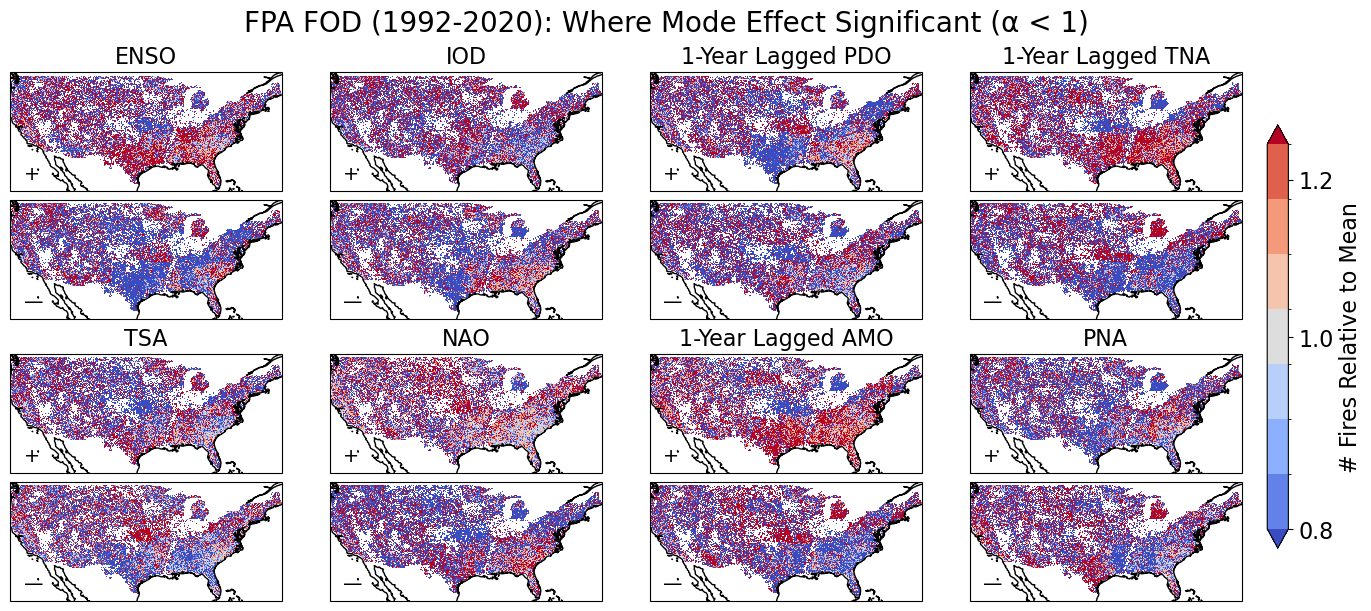


*Supplementary Figure 2.4: the association between the FPA FOD fire occurrence data and key climate modes when no significance level is considered. High levels of noise and areas with insufficient data mean that a trend cannot be identified. The sign and magnitude of the relationship is given by the ratio between the annual number of wildfires in the positive (upper panels) or negative (lower panels) phase – defined as beyond plus or minus half a standard deviation from the mean respectively. The effect of each mode is shown relative to the mean annual number of wildfires – to account for any non-linearity in the effect between phases that would not be captured by linear regression. Note that the FPA FOD starts at 1992, hence the curtailed time period.*

*
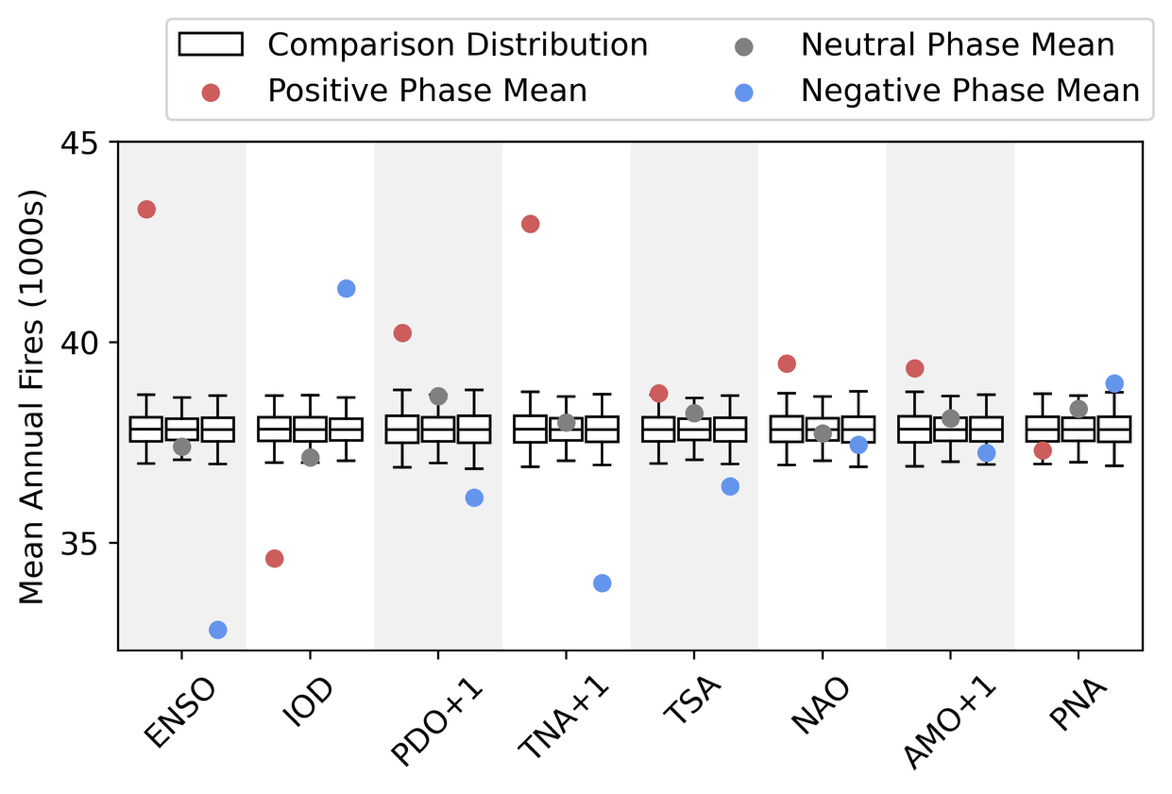
*

*Supplementary Figure 2.5: The distribution of the modelled annual number of wildfires for the recent climate ensemble under different mode phases compared to the distribution of all years, per Shen et al. (2025). The dots show the actual mean value of modelled annual fires. The boxplots show the distribution of the mean number of annual fires, drawn from the distribution of years but with the same sample size as the number of years in that phase in the reanalysis period – repeated 10,000 times. The outer limits of the boxplots represent the 2.5th and 97.5th percentiles, and the three internal lines correspond to the 25th, 50th and 75th percentiles of the bootstrapped distribution.*

### **Supplementary Section 3: Context on Regions and Climate Change Signal**

*
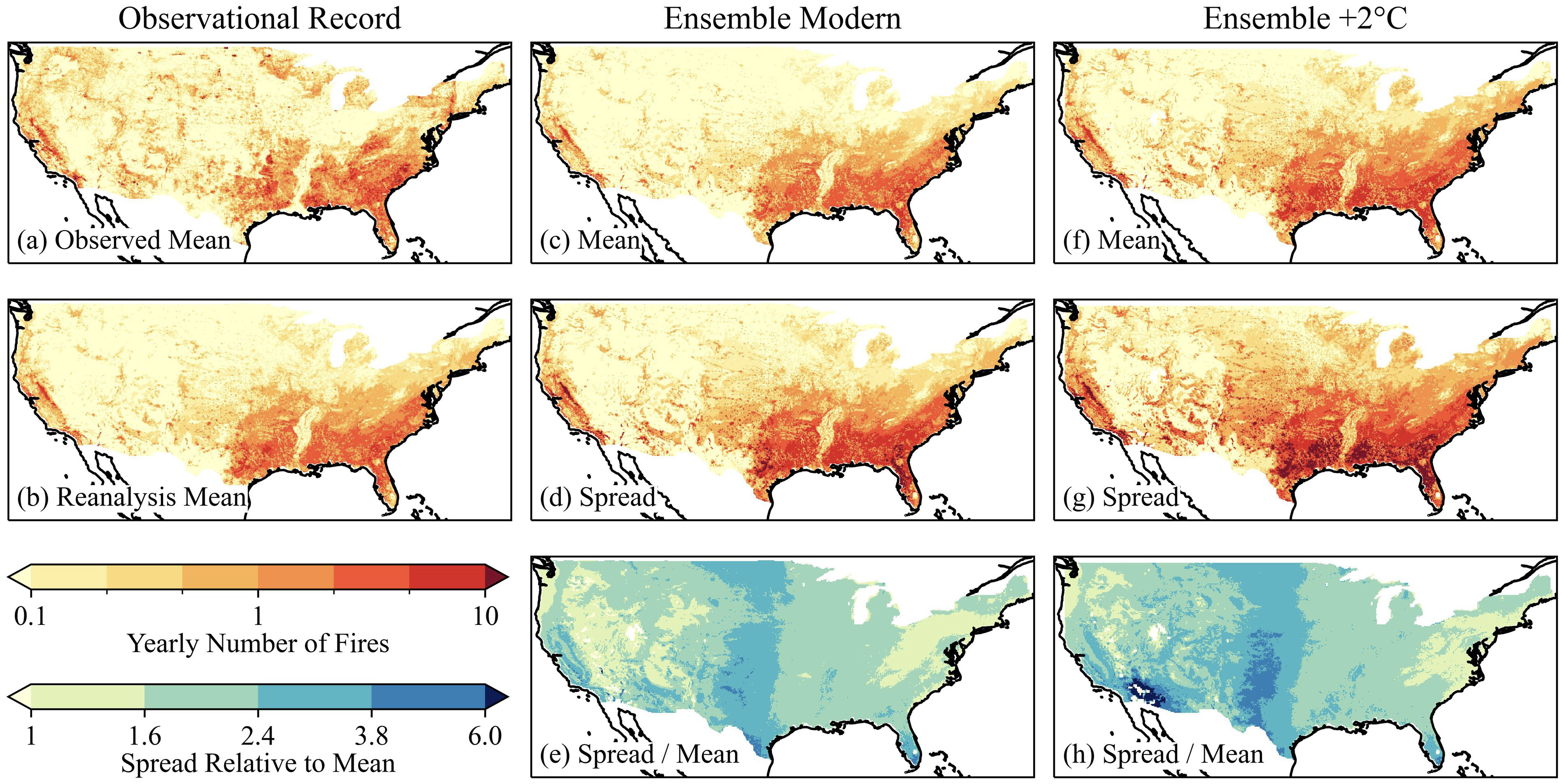
*

*Supplementary Figure 3.1: Reproduced from Keeping et al. (2025). Modelled and observed patterns in the annual number of wildfires greater than 0.1 hectares, with both the mean and 1st-99th percentile spread shown. The plots show (a) the observed annual mean of the wildfire occurrence record for 1992–2020; (b) the modelled reanalysis mean for 1990–2019; (c) the modelled ensemble mean for the modern (2000–2009 climate); (d) the modelled ensemble spread for the modern; (e) the ratio of model spread and mean for the ensemble modern; (f) the +2°C ensemble mean (2000–2009 climate plus 2°C of warming); (g) the +2°C ensemble spread; and (h) the ratio of model spread and mean for the +2°C ensemble. Note that the FPA FOD starts at 1992, hence the slightly curtailed time period.*


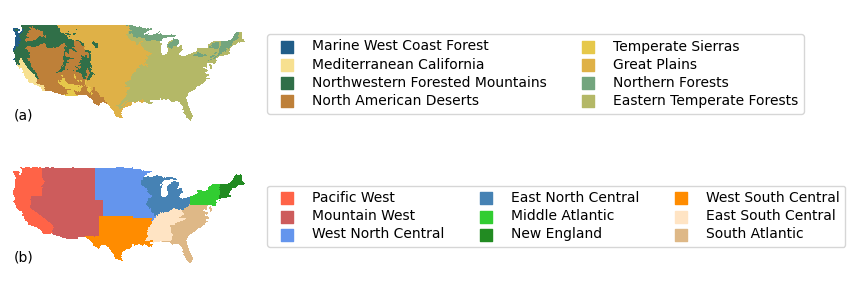


*Supplementary Figure 3.2: an overview of (a) the ecoregions and (b) the National Interagency Fire Center Geographic Area Coordination Center (NIFC GACC) regions referred to in this study.*

### **Supplementary Section 4: Bias Correction Performance**

*Supplementary Table 4.1: KNMI-LENTIS bias correction performance statistics in comparison to ERA5 data for input into fire occurrence model. Based on correlation of top four moments for data aggregated monthly at 2π * ensemble resolution. The variables are mean daily surface windspeed (sfcwind); daytime mean vapour pressure deficit (vpd); diurnal temperature range (dtr); precipitation (pr); prior 5-day precipitation (pr_5d); prior 50-day GPP (GPP_50d); prior year GPP (GPP_1yr).*

| R^2^ | sfcwind | vpd | dtr | snc | pr | pr_5d | GPP_50d | GPP_1yr |
| --- | --- | --- | --- | --- | --- | --- | --- | --- |
| Mean | 0.922 | 0.987 | 0.983 | 0.994 | 0.985 | 0.985 | 0.881 | 0.773 |
| Variance | 0.567 | 0.812 | 0.684 | 0.409 | 0.726 | 0.771 | <0 | <0 |
| Skewness | 0.073 | <0 | 0.741 | 0.098 | 0.110 | 0.307 | <0 | 0.531 |
| Kurtosis | 0.020 | <0 | 0.052 | <0 | <0 | <0 | <0 | 0.266 |

### **Supplementary Section 5: Global Climate Mode Representation in KNMI-LENTIS**

Supplementary Figures 5.1-5.11 show that the Sea Level Pressure (SLP) and Sea Surface Temperature (SST) phenomena (specified in each figure caption) associated with each global climate mode are all adequately represented in the KNMI-LENTIS ensemble. The Atlantic Multidecadal Oscillation (AMO) shows the characteristic AMO+ warm pool in the northern Atlantic, with the correct region of highest anomaly off the Greenland coast. The Tropical South Atlantic (TSA) shows the characteristic TSA+ warm sea surface temperatures below West Africa, spreading in effect to the Caribbean Sea. The Tropical North Atlantic (TSA) shows the expected TNA+ warm band in the northern tropics, with cool effects towards the equator and in the extratropical west Atlantic. The Southern Annular Mode (SAM) shows the expected low pressure system over Antarctica associated with the SAM+. The Pacific/North American (PNA) oscillations strongly shows the expected PNA+ low in the northeastern Pacific; but does not show clearly the expected western US high and eastern US low – though the associated arctic high in the PNA+ is present. The Pacific Decadal Oscillation (PDO) shows the expected cool sea surface temperature jet off the west coast of Japan, and the pooling of warmer water against West North America. The North Atlantic Oscillation (NAO) shows the expected sea surface temperature quadrupole with warmer sea surface temperatures of the eastern US and in Northern Europe, and cooler sea surface temperatures off Greenland and West Africa. The Indian Ocean Dipole (IOD) shows the expected gradient of cool to warm sea surface temperatures from the west to east Indian Ocean in the negative mode. El Niño Southern Oscillation (ENSO) shows the expected warm jet along the equator in El Niño (SOI-) with cooler sea surface temperature pools north and south of it in the west and central Pacific. The East Atlantic (EA) oscillation shows the northern pressure low and southern pressure high in the Atlantic associated with the EA+. The Arctic Oscillation (AO) shows the expected low pressure system over the Arctic associated with the AO+.

The effect of each global climate mode (Supplementary Figure 5.12) on precipitation patterns over the contiguous US in the recent climate ensemble is consistent with expectations. For ENSO, wetter conditions are expected during La Niña in the northwestern and inland East US, and during El Niño in the southern latitudes of the US (Ropelewski and Halpert, 1987). These features are all well represented in the ensemble. For the IOD, precipitation conditions are expected to be highly correlated to ENSO, with limited independent influence (Hu et al., 2023). This is matched in the ensemble, with the IOD showing highly similar patterns to ENSO but with a lesser overall magnitude of effect. For the PDO, southwestern precipitation is associated with a strong increase in the positive phase (Dai, 2013), in other regions the effect is limited for the annually averaged index (Kumar et al., 2013). This is well represented in the ensemble. For the TNA, the association with precipitation, like the IOD, is confounded by its close association with ENSO (Kushnir et al., 2010). The association with ENSO is matched in the ensemble, with the precipitation pattern a weakened version of that observed under ENSO. The TSA is not association with US precipitation. In the ensemble it has a minor negative effect in the central US. For the NAO, higher winter precipitation can result from a positive phase in the eastern US (Ning and Bradley, 2014), but this effect is dominated by ENSO (Tang et al., 2023; Ning and Bradley, 2014) whilst in the southwestern US conditions are wetter in the negative phase including when controlling for ENSO (Tang et al., 2023). In the ensemble this southwestern effect is well-represented, whilst the eastern effect is not, this can be explained by the dominant effect of ENSO on precipitation in the region. For the AMO, a decrease on contiguous US precipitation is associated with the positive phase, though this effect is patchy over the region (Hu et al., 2011). No clear effect is apparent in the ensemble data, possible due to the index signal not being smoothed over a decadal timescale. For the PNA, the positive phase is associated with elevated rainfall in the great Plains and East coast and reduced rainfall in the northwestern US (Leathers et al., 1991). In the ensemble, the East coast and Great Plains effects are seen, but there is an additional stronger effect in the southwestern US as well as no effect in the northwest; this can be explained by correlation (Soulard et al., 2019) between the positive PNA and El Niño. For the AO, high precipitation totals are expected over the central US (Hu and Feng, 2010). This effect is well represented in the ensemble, but an additional effect is present in the southwestern US; this can be explained by correlation (Simpkins, 2021) between the AO and NAO. The EA is not associated with an effect on US meteorology, but has been linked to a modulation of the NAO (Rodrigo, 2021). The ensemble shows limited diverging effect on precipitation between its phases. The SAM is not associated with a direct impact on US meteorology. The ensemble shows a minor positive effect for the central US. Overall, the major effects on precipitation are well-represented in the ensemble, although confounding effects from correlation and modulating-effects between modes can render the signal hard to attribute.

The transient, continuous runs of the two multidecadal modes both show clear decadal or longer time-period oscillations (Supplementary Figures 5.13-5.14). The amplitude of multidecadal oscillations in the AMO is comparable to the amplitude of sub-decadal oscillations, meaning that a clear multidecadal signal emerges in the annual index values considered in this study. On the other hand, the sub-decadal timescale oscillations in the PDO are significantly greater than the multidecadal oscillations, meaning that a multidecadal effect does not emerge and sub-decadal oscillations dominate as the key effect in this study.


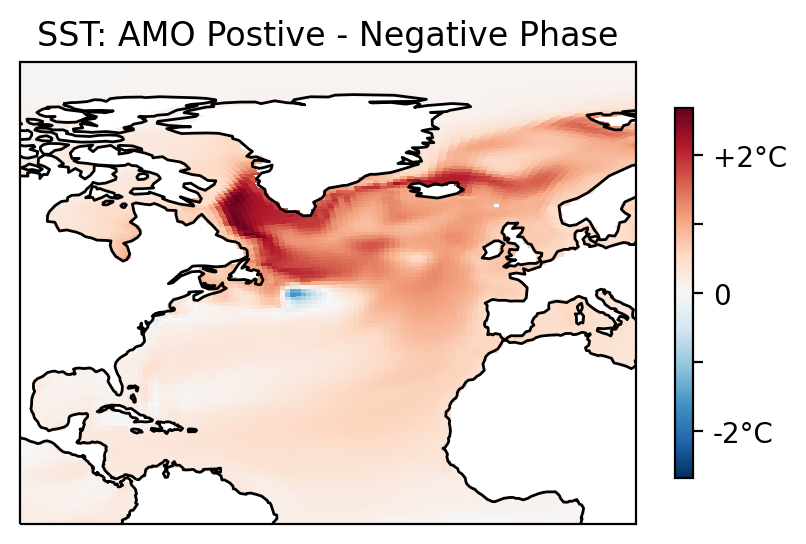


*Supplementary Figure 5.1: The sea surface temperature pattern of the Atlantic Multidecadal Oscillation (AMO) in the recent climate ensemble, showing the characteristic AMO+ warm pool in the northern Atlantic, with the correct region of highest anomaly off the Greenland coast.*


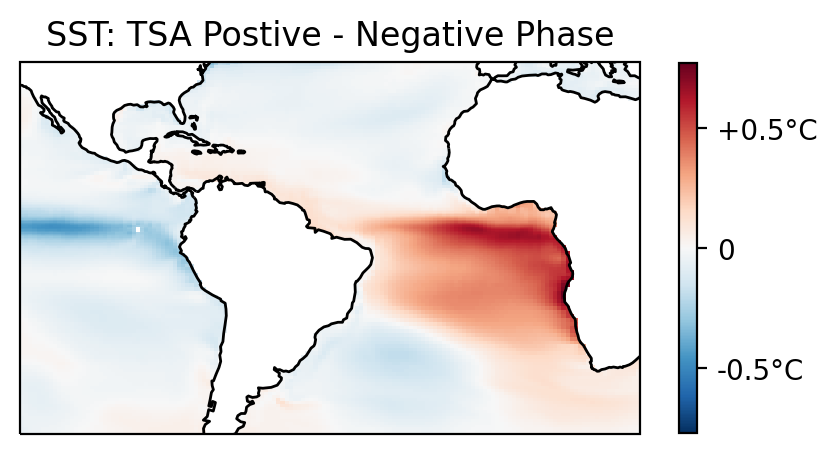


*Supplementary Figure 5.2: The sea surface temperature pattern of the Tropical South Atlantic (TSA) in the recent climate ensemble, showing the characteristic TSA+ warm sea surface temperatures below West Africa, spreading in effect to the Caribbean Sea.*


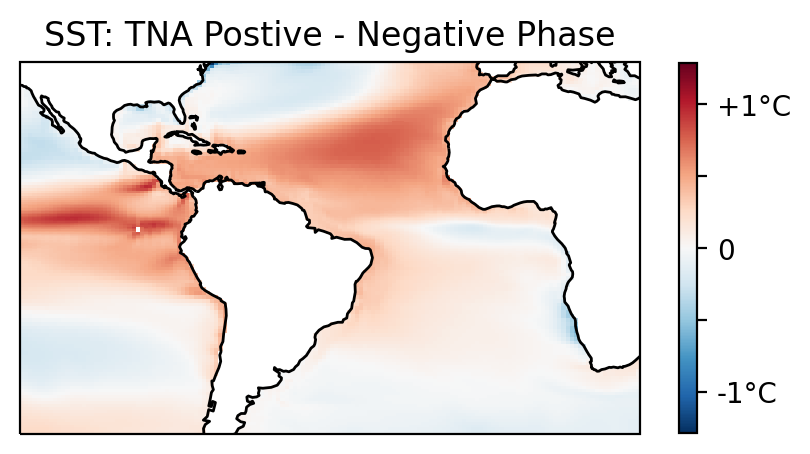


*Supplementary Figure 5.3: The sea surface temperature pattern of the Tropical North Atlantic (TNA) in the recent climate ensemble, the TNA+ warm band in the northern tropics, with cool effects towards the equator and in the extratropical west Atlantic.*


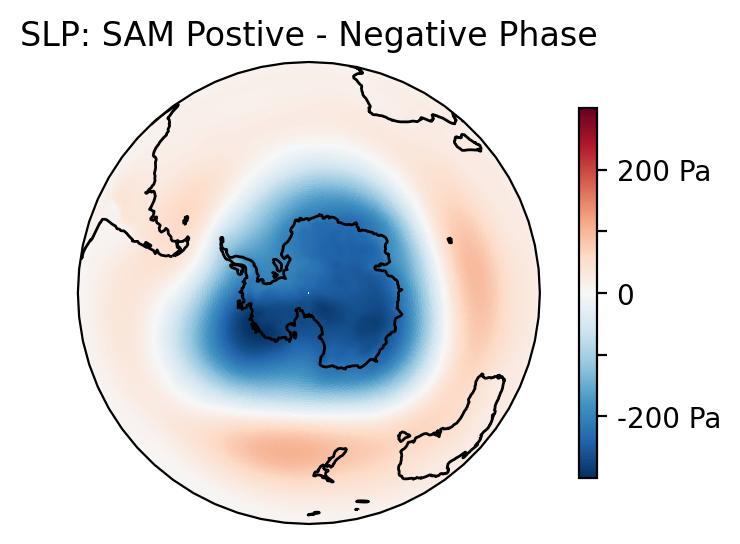


*Supplementary Figure 5.4: The sea-level pressure pattern of the Southern Annular Mode (SAM) in the recent climate ensemble, showing the expected low pressure system over Antarctica associated with the SAM+.*


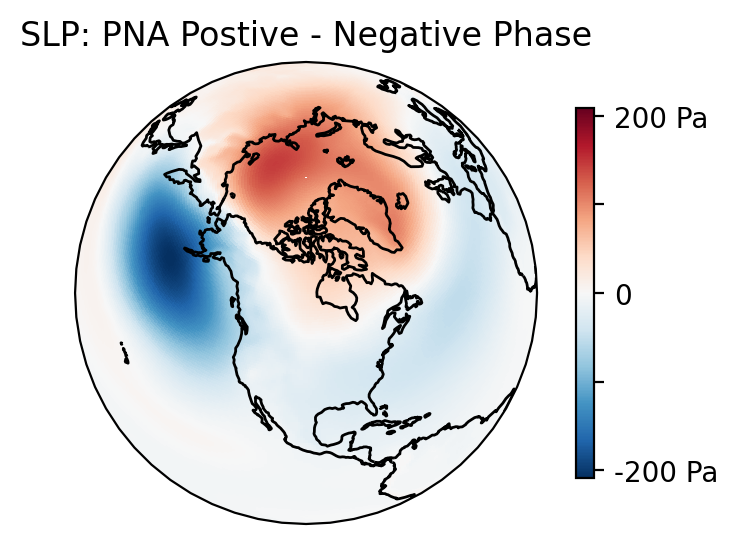


*Supplementary Figure 5.5: The sea-level pressure pattern of the Pacific/North America (PNA) in the recent climate ensemble, showing strongly the expected PNA+ low in the northeastern Pacific. Does not show clearly the expected western US high and eastern US low, though the associated arctic high in the PNA+ is present.*


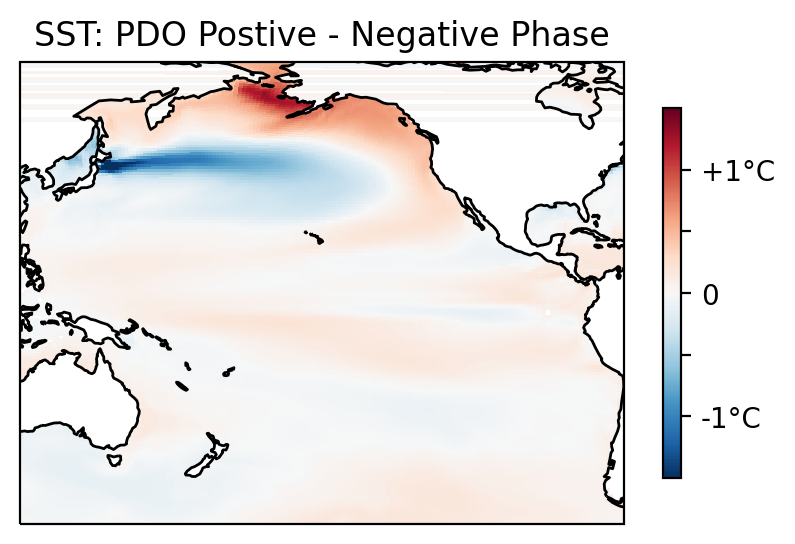


*Supplementary Figure 5.6: The sea surface temperature pattern of the Pacific Decadal Oscillation (PDO) in the recent climate ensemble, showing the expected cool sea surface temperature jet off the west coast of Japan, and the pooling of warmer water against West North America.*


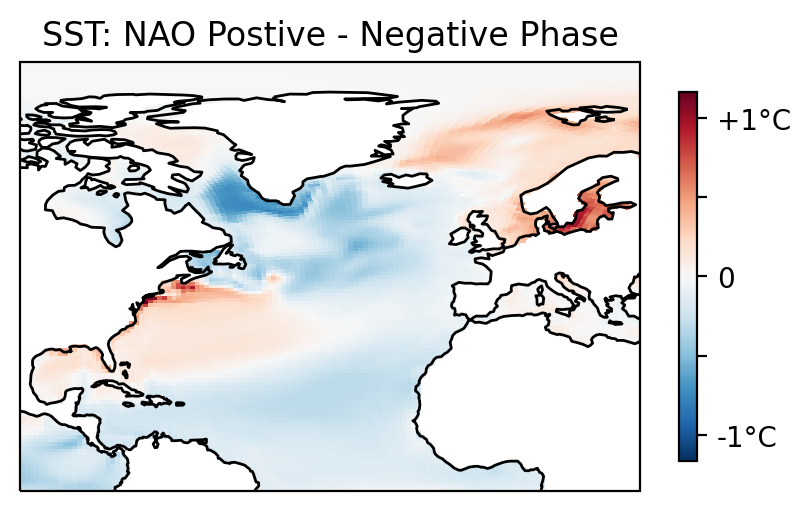


*Supplementary Figure 5.7: The sea surface temperature pattern of the North Atlantic Oscillation (NAO) in the recent climate ensemble, showing the expected sea surface temperature quadrupole with warmer sea surface temperatures of the eastern US and in Northern Europe, and cooler sea surface temperatures off Greenland and West Africa.*


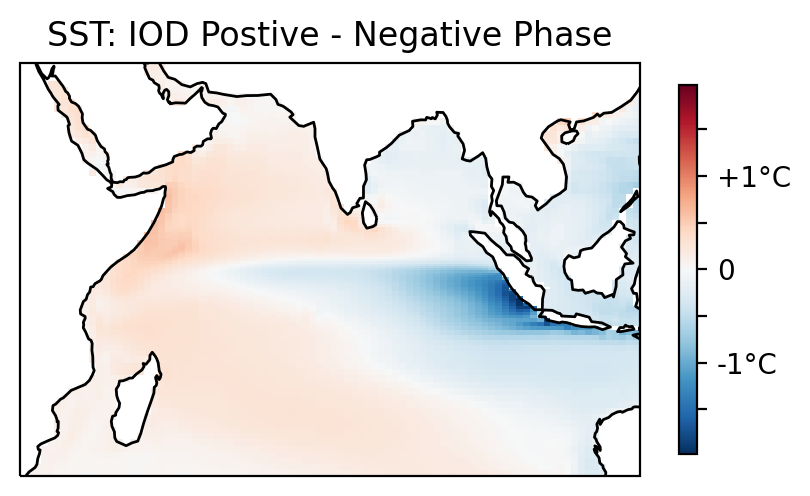


*Supplementary Figure 5.8: The sea surface temperature pattern of the Indian Ocean Dipole (IOD) in the recent climate ensemble, showing the expected gradient of cool to warm sea surface temperatures from the west to east Indian Ocean in the negative mode.*


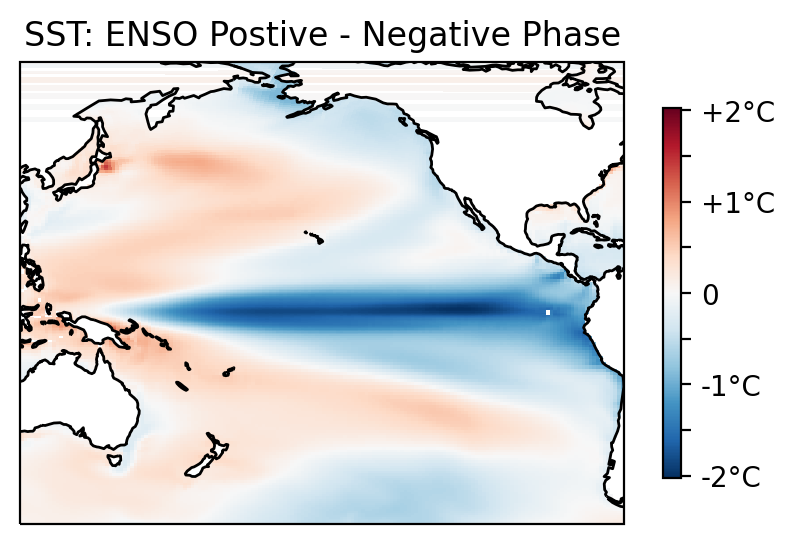


*Supplementary Figure 5.9: The sea surface temperature pattern of El Niño Southern Oscillation (ENSO) in the recent climate ensemble, this shows the expected warm jet along the equator in El Niño (SOI-) with cooler sea surface temperature pools north and south of it in the west and central Pacific.*


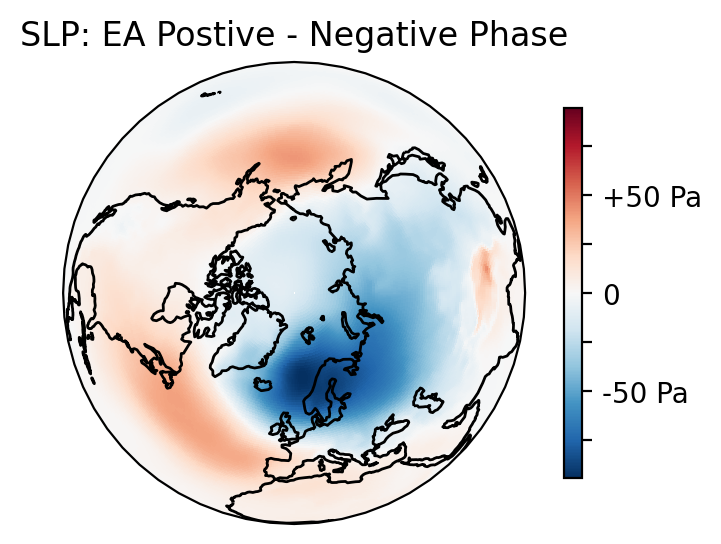


*Supplementary Figure 5.10: The sea-level pressure pattern of the East Atlantic (EA) in the recent climate ensemble, showing a northern pressure low and southern pressure high in the Atlantic associated with the EA+.*


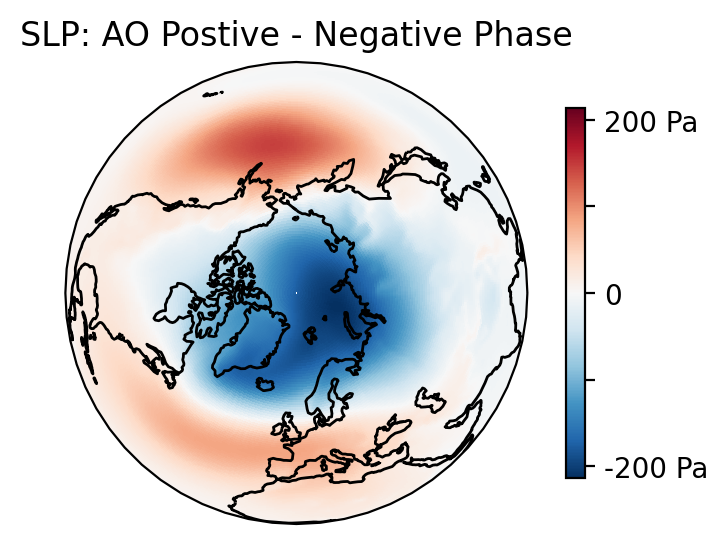


*Supplementary Figure 5.11: The sea-level pressure pattern of the Arctic Oscillation (AO) in the recent climate ensemble, showing the expected low pressure system over the Arctic associated with the AO+.*


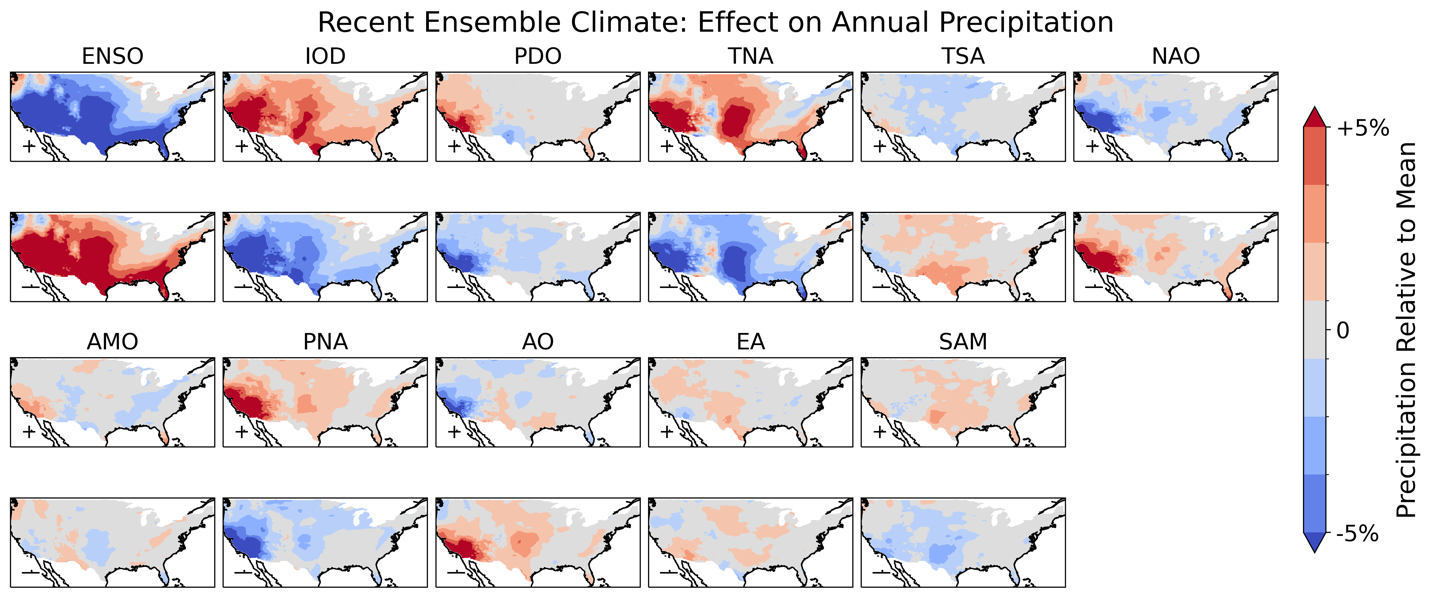


*Supplementary Figure 5.12: The effect of the phase of each climate mode (greater or lesser than half of a standard deviation from zero) on annual precipitation totals relative to the mean rate.*


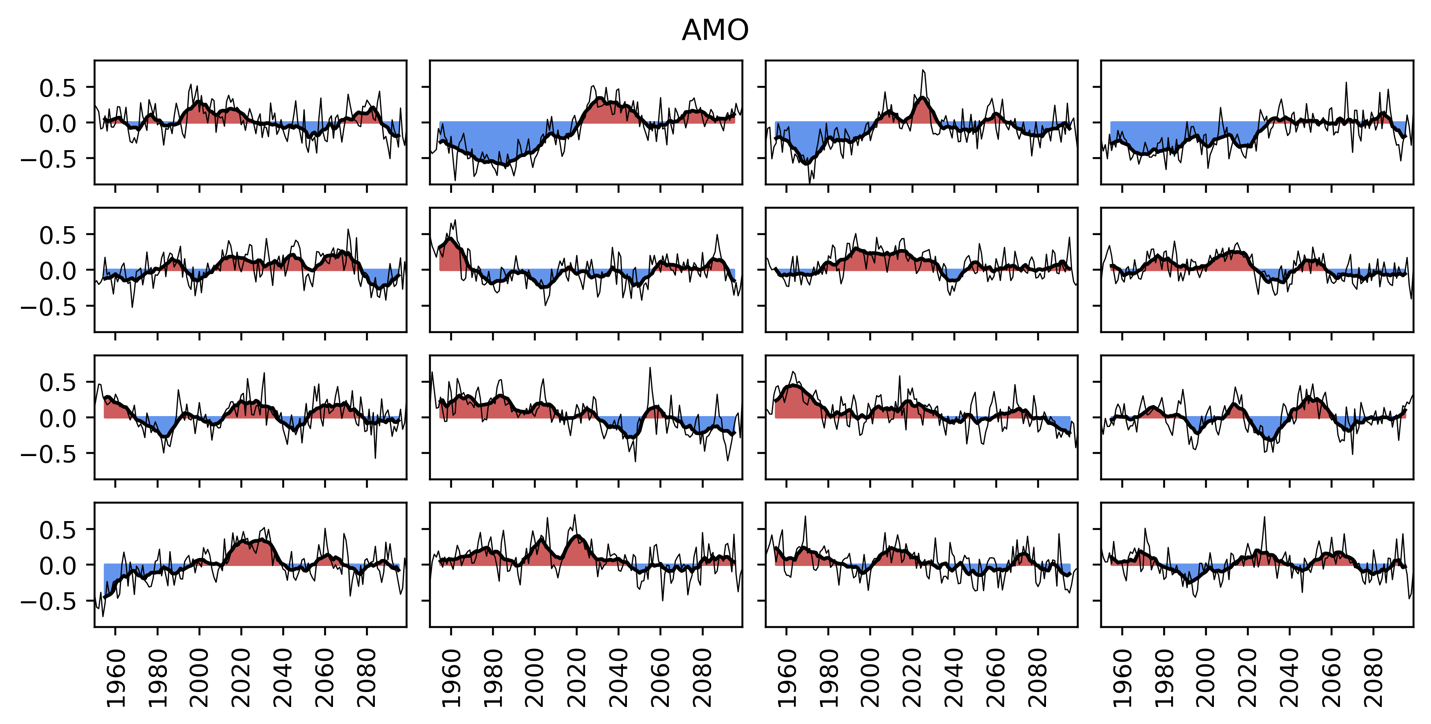


*Supplementary Figure 5.13: The 16 transient runs of the AMO index from which the 160 ensemble members for each time-slice are derived. The thin line shows the annual timeseries and the thicker, under-filled line shows the decadally smoothed, centered average.*


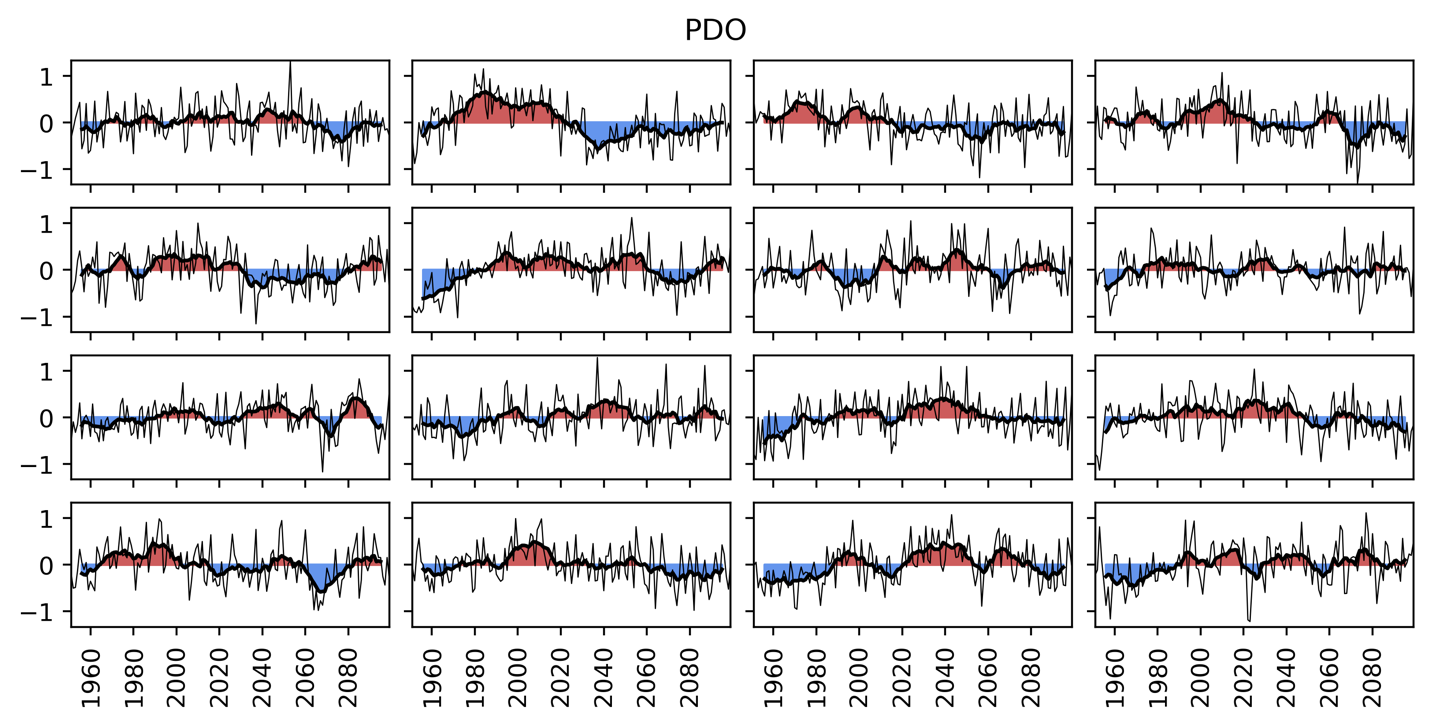


*Supplementary Figure 5.14: The 16 transient runs of the PDO index from which the 160 ensemble members for each time-slice are derived. The thin line shows the annual timeseries and the thicker, under-filled line shows the decadally smoothed, centered average.*

*
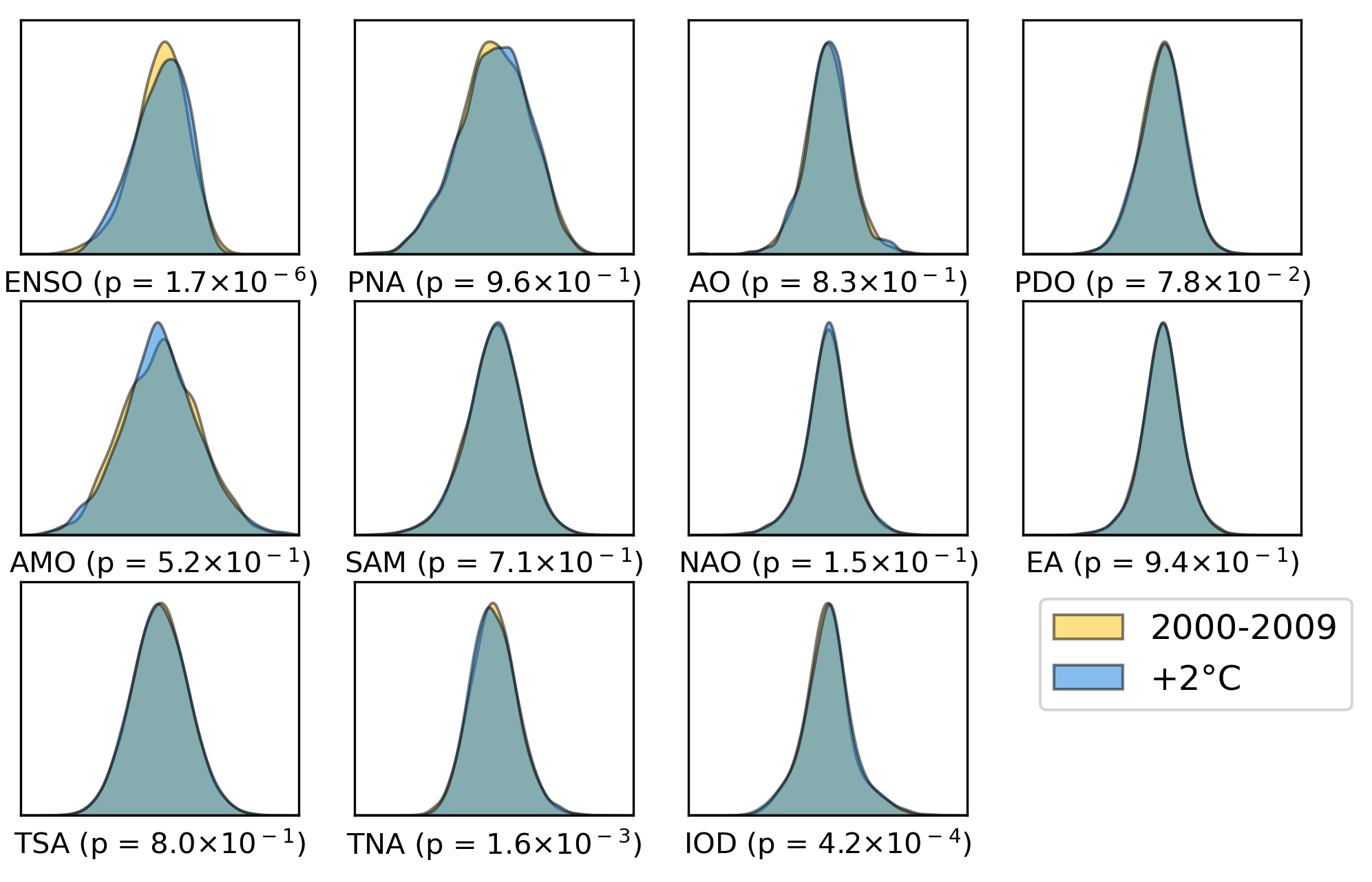
*

*Supplementary Figure 5.15: Distributions, with K-S test p-values for difference in distribution in both periods. There are small but statistically significant shifts in the ENSO, IOD and TNA distributions.*

*
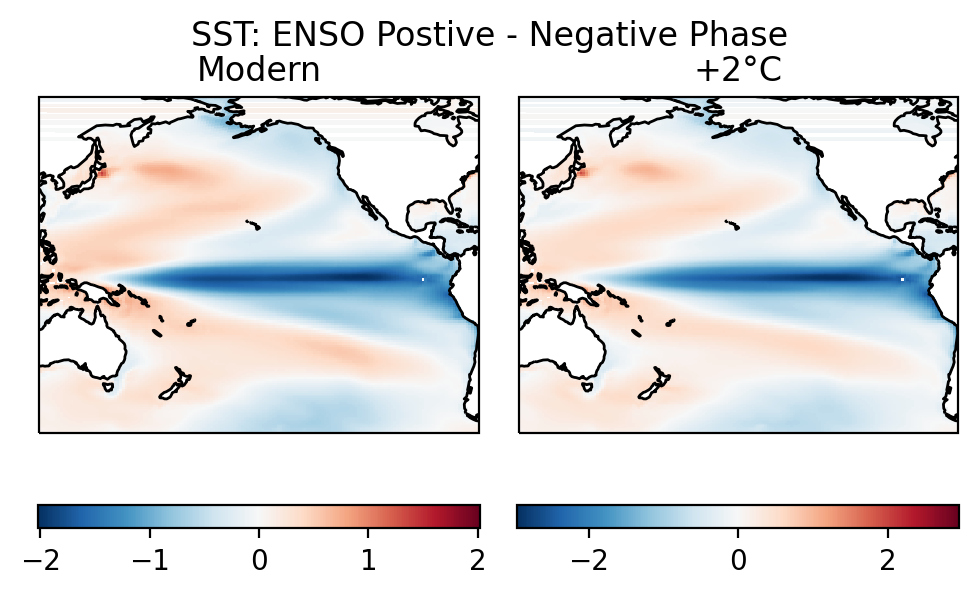
*

*Supplementary Figure 5.16: The sea surface temperature pattern of El Niño Southern Oscillation (ENSO) in the recent (left) and future (right) time slice of the climate ensemble.*

### **Supplementary Section 6: Correlations Between ENSO, IOD and TNA+1**

The El Niño Southern Oscillation (ENSO) is correlated with the Indian Ocean Dipole (IOD) and Tropical North Atlantic (TNA) in the ensemble. The below scatter plots given an overview of that relationship in the ensemble recent and +2°C climates, the TNA+1 is correlated to ENSO, whilst the IOD is more strongly anti-correlated. Both modes become more strongly correlated with future warming.

*
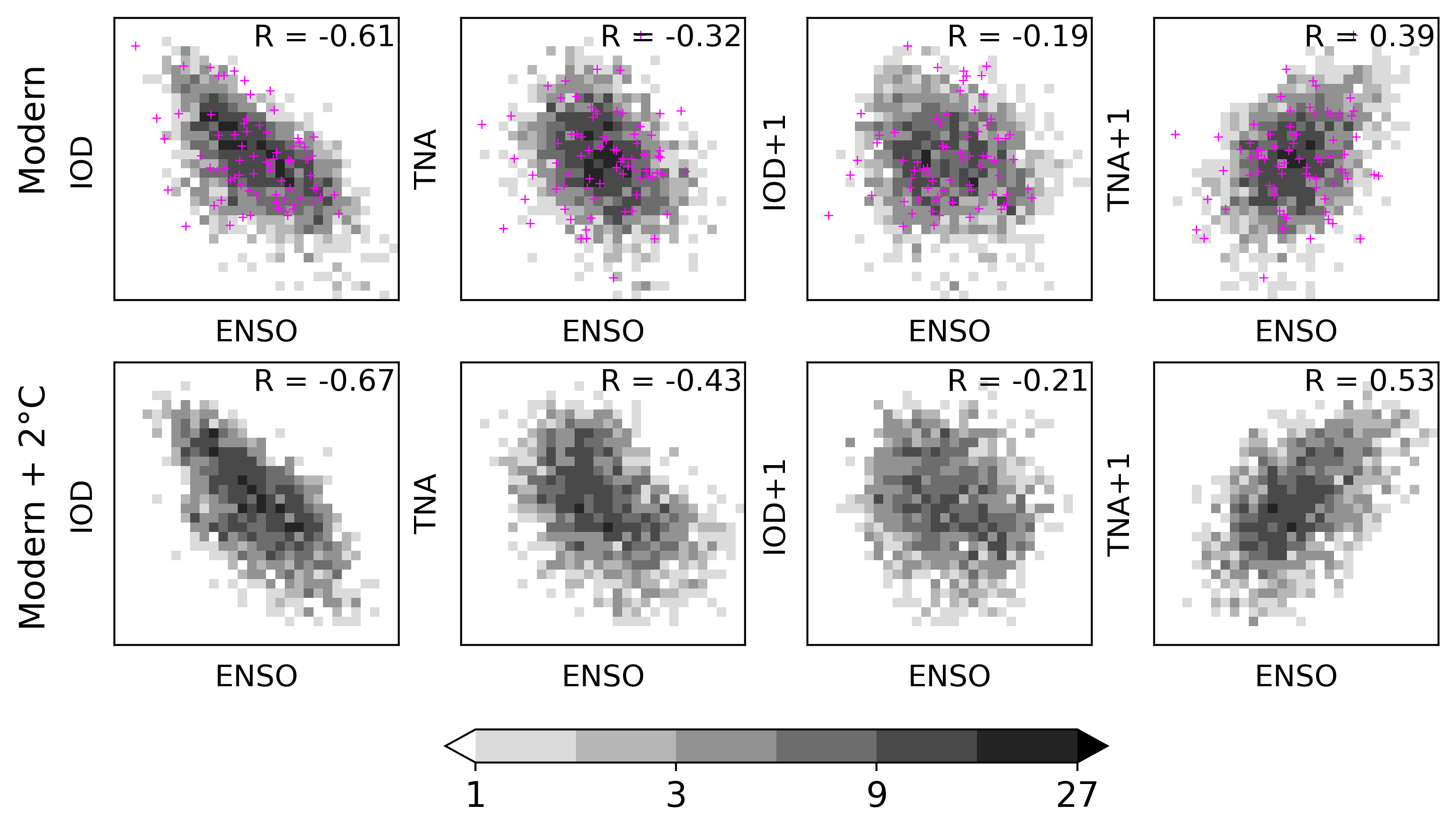
*

*Supplementary Figure 6.1: Scatterplots showing relationship between ENSO, IOD and TNA. Each datapoint is one annual value. Reanalysis data overlaid with crosses.*

**

*Supplementary Figure 6.2: Scatterplots of annual temperature and precipitation averaged over CONUS against climate mode values for the ensemble 2000-2009 climate. Each datapoint is one annual value. Reanalysis values are overlayed as crosses for the period 1990-2019.*

### **Supplementary Section 7: Areal Effect of Climate Modes in Recent Climate**

This section shows (Supplementary Figure 7.1) the area of positive, negative and insignificant effect of each mode to a much high threshold of significance – finding that La Niña, the negative Indian Ocean Dipole (IOD), and positive 1-year lagged Tropical North Atlantic all persist as strongly associated with wildfire occurrence. The areal effect of each mode is also broken down by meteorological season (Supplementary Figure 7.2), with modes having the greatest area of significant effect on relative change in wildfire occurrence during summer (June to August, JJA) except for the Pacific Decadal Oscillation (December to February, DJF) and Tropical South Atlantic oscillation (March to May, MAM). Maps showing the area and magnitude of the effect for each mode not shown in the main text (Figure 4) are presented in Supplementary Figure 7.3, the major effects are the Pacific Decadal Oscillation, Tropical North Atlantic, and 1-year lagged El Niño Southern Oscillation.


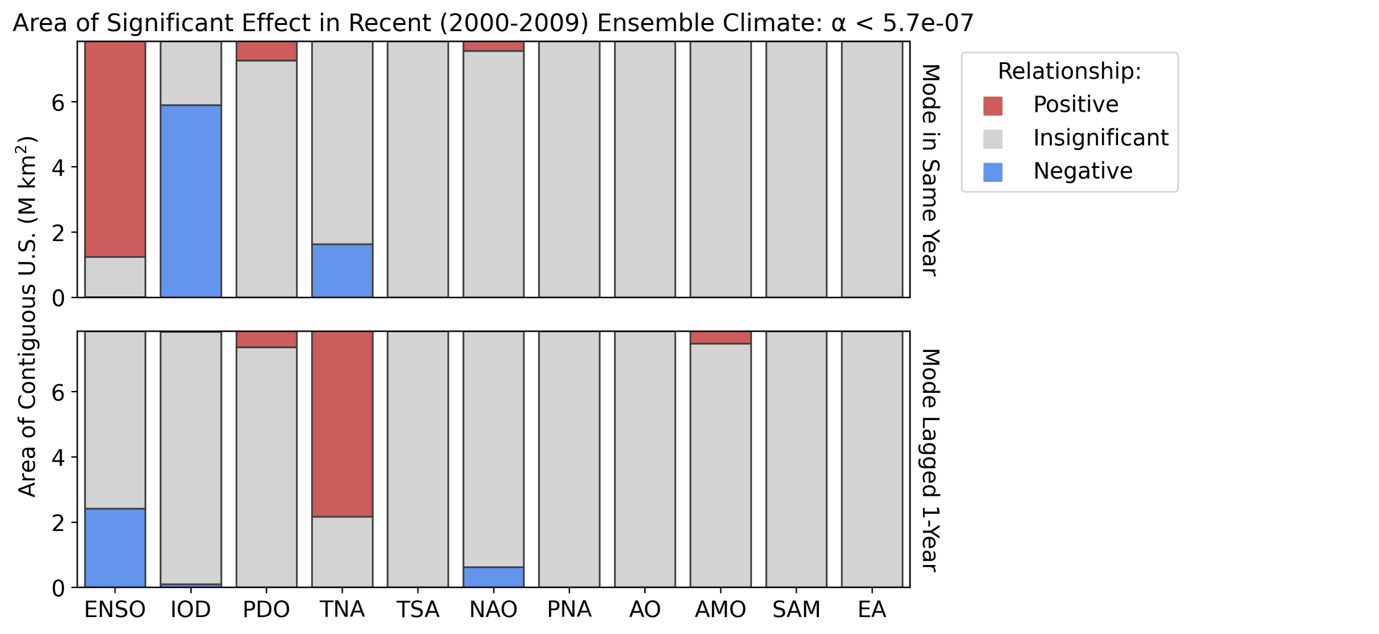


*Supplementary Figure 7.1: The area of significant effect for each climate mode over the contiguous US, to an extremely strict FDR-corrected significance threshold of 5.7 x 10^-7^ (equivalent to 5-sigma)*

*
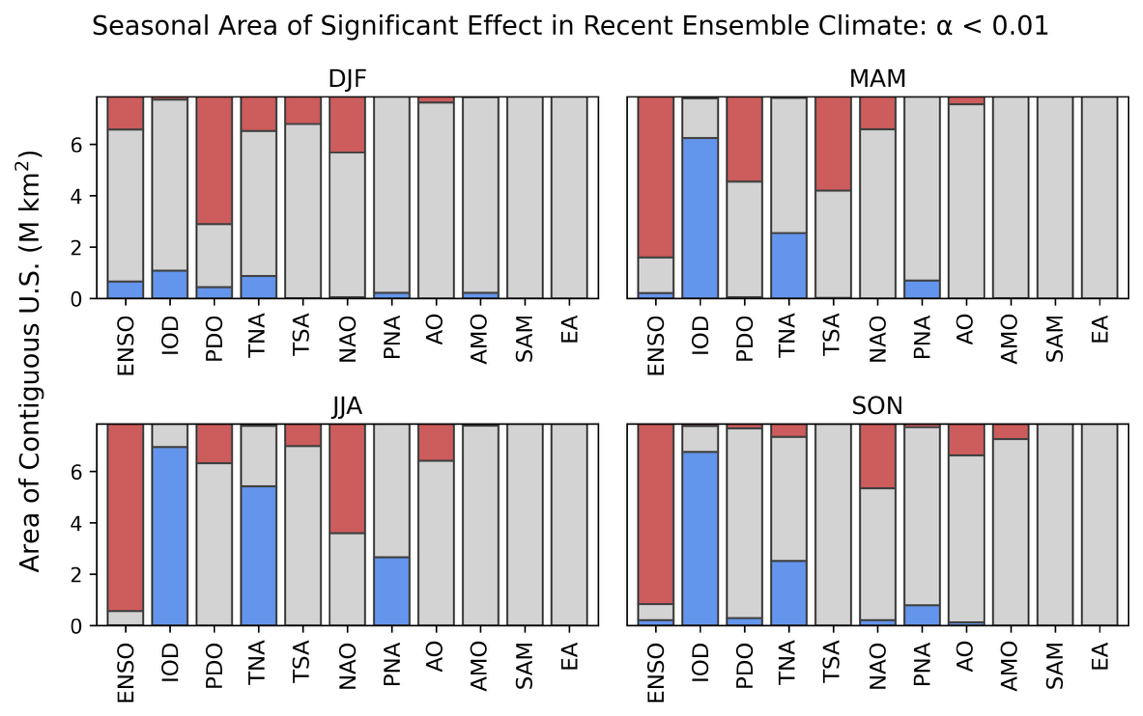
*

*Supplementary Figure 7.2: The seasonal (DJF, MAM, JJA, SON) areas of significant effect for each climate mode over the contiguous US. Significance was determined by the p-value of the linear regression between the climate mode’s index value and the seasonal number of fires in each 0.1° grid-cell, to an FDR-corrected significance threshold of 0.01. The slope of the regression determined the sign of the relationship between annual fires and the index.*


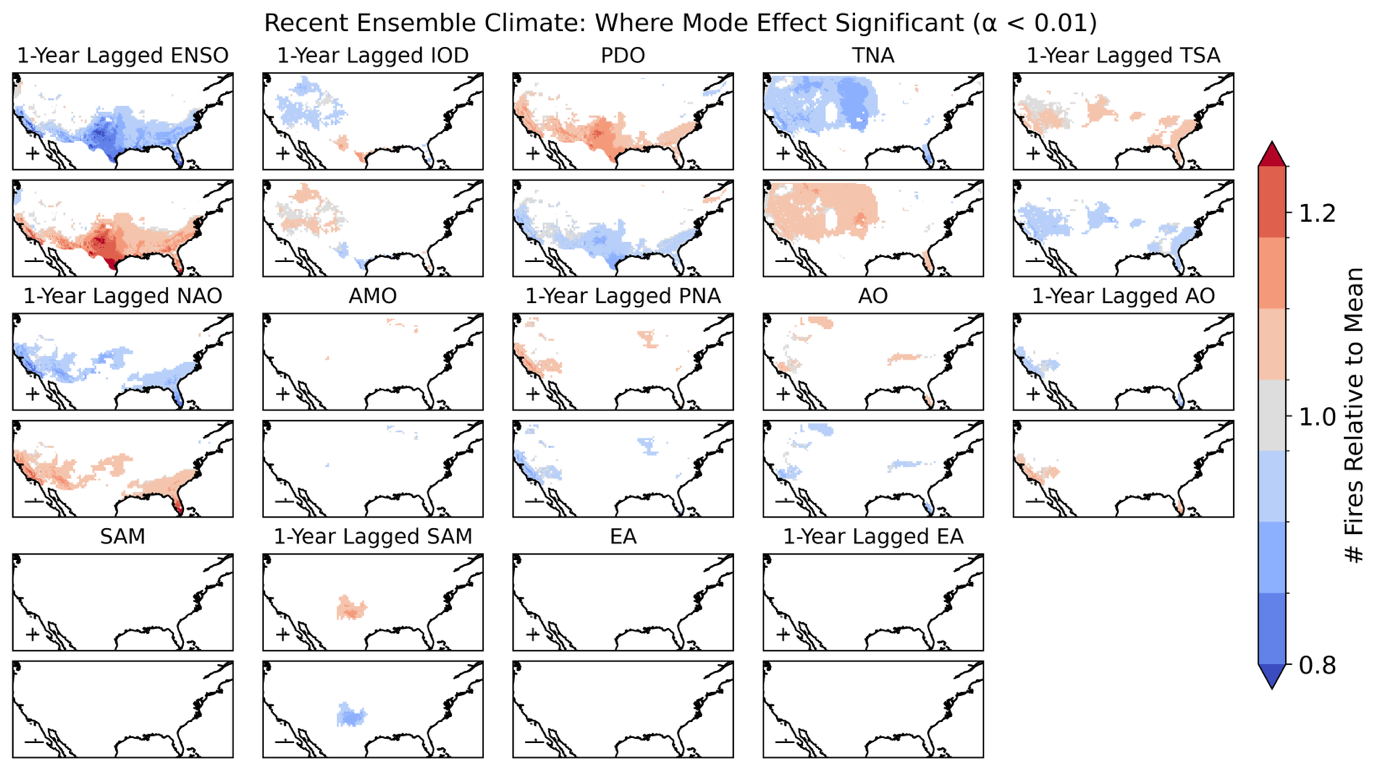


*Supplementary Figure 7.3: For all modes not shown in figure 4, showing locations for which linear regression determined a significant relationship between the annual number of fires and the climate mode’s index; the ratio between the annual number of fires in the positive and negative phases of each mode relative to the mean annual number of fires.*

### **Supplementary Section 8: Effect of Climate Modes on Wildfire Drivers**


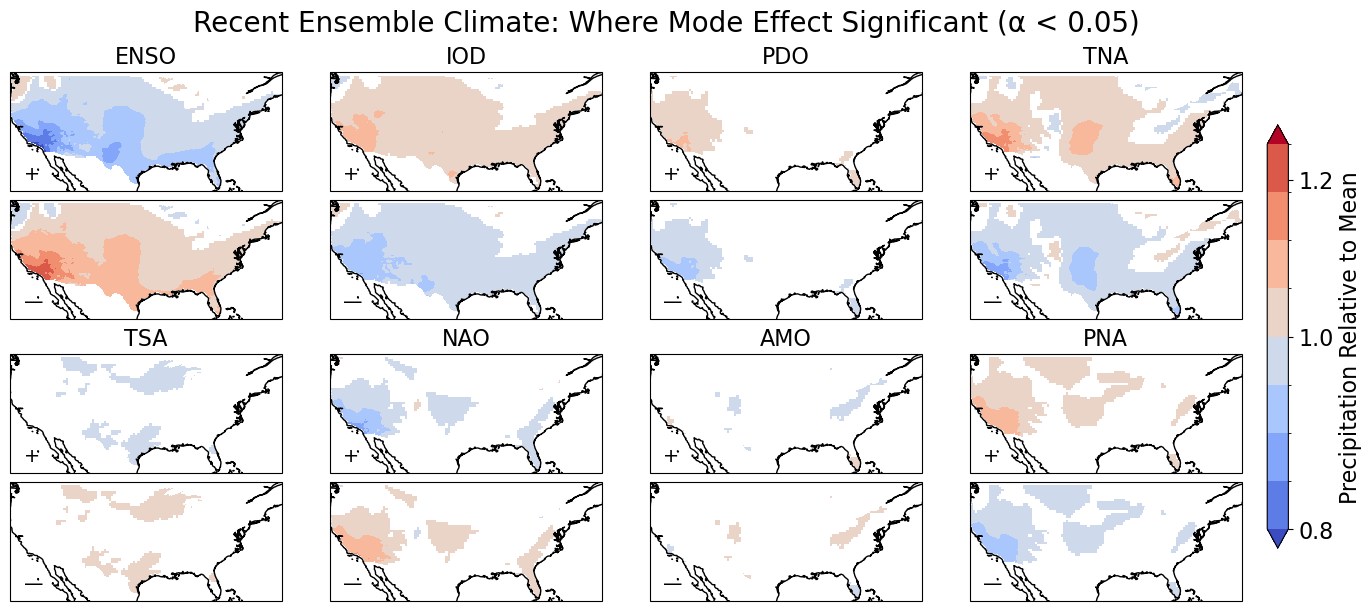


*Supplementary Figure 8.1: the relative influence of climate modes on annual precipitation in the recent climate for the large ensemble.*


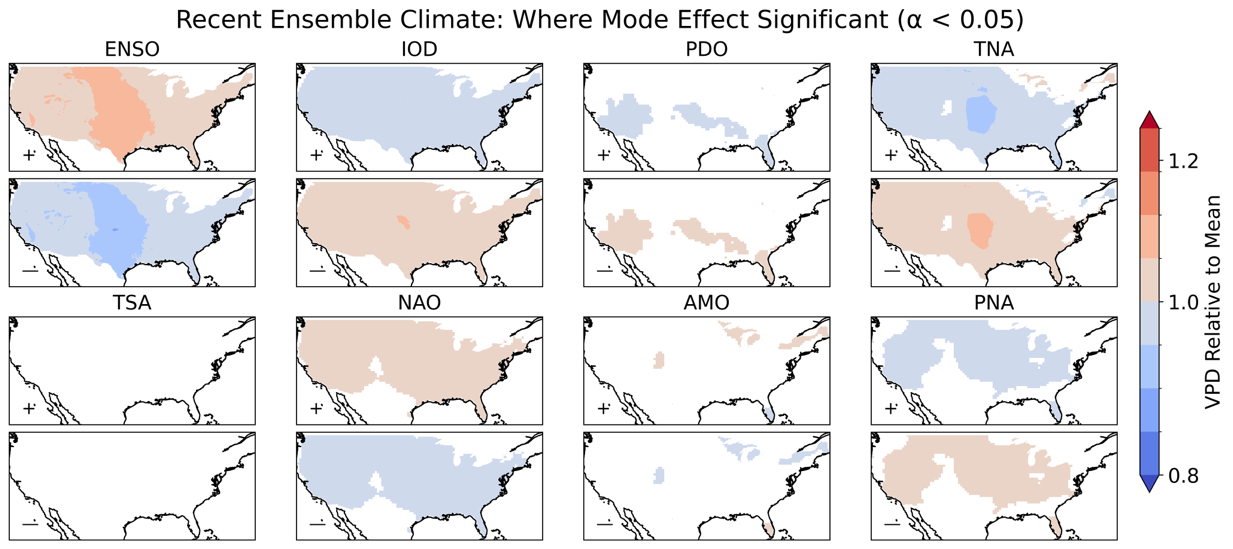


*Supplementary Figure 8.2: the relative influence of climate modes on annual VPD in the recent climate for the large ensemble.*


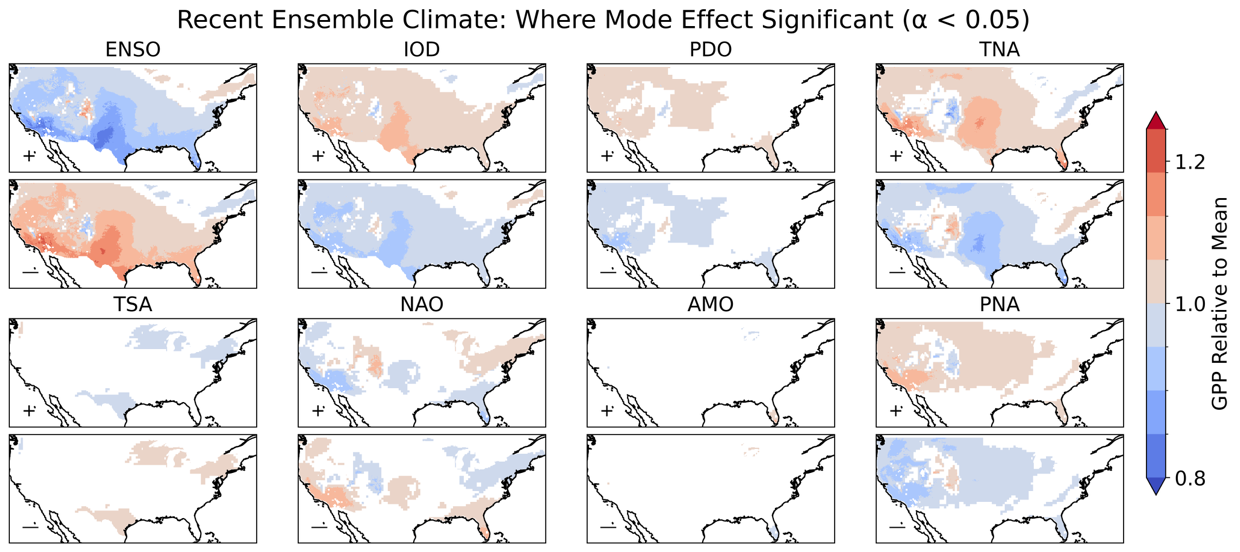


*Supplementary Figure 8.3: the relative influence of climate modes on annual GPP in the recent climate for the large ensemble.*

*
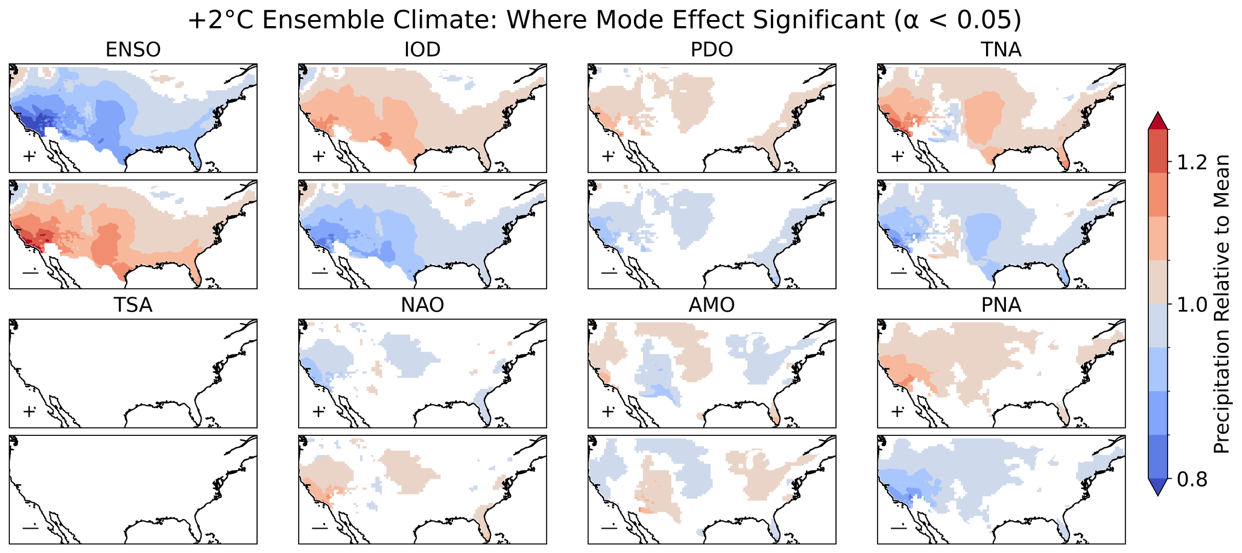
*

*Supplementary Figure 8.4: the relative influence of climate modes on annual precipitation in the +2°C climate for the large ensemble.*

*
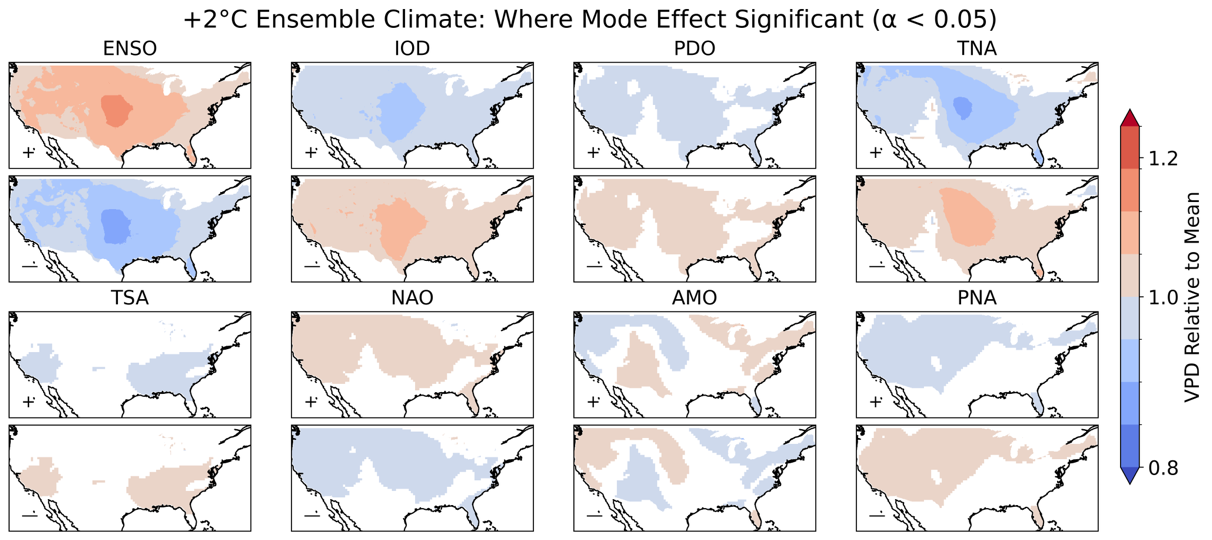
*

*Supplementary Figure 8.5: the relative influence of climate modes on annual VPD in the +2°C climate for the large ensemble.*

*
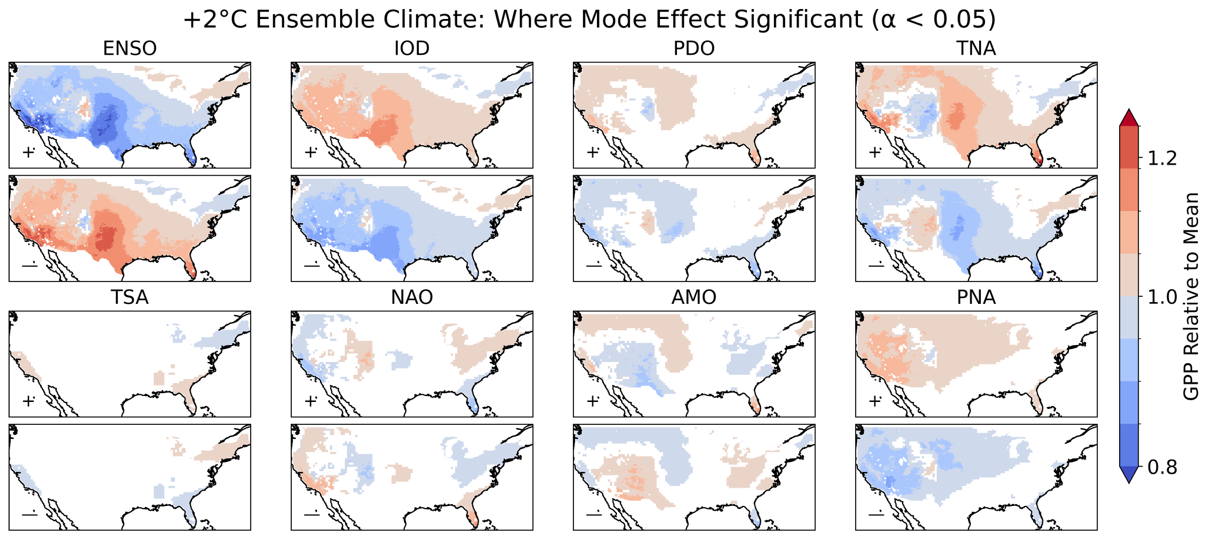
*

*Supplementary Figure 8.6: the relative influence of climate modes on annual GPP in the +2°C climate for the large ensemble.*

### **Supplementary Section 9: Regional probability distribution functions of Annual Wildfires Given Climate Mode Phase**

This section shows the difference in the positive and negative phase distributions for all wildfire administrative fire regions in the US (Geographic Area Coordination Centres) – for the AMO+1, TSA, TNA+1, PNA, PDO+1, NAO, IOD, and ENSO. In some regions, the mode’s effect is to spread the distribution further in the wildfire prone phase, such as in Southern California or the Southwest, whilst in others the mode’s effect is solely on the distribution centre, such as in Northern California or the Great Basin. The strongest effects on the distribution are that of ENSO, the TNA+1 and IOD, whilst the AMO+1, PDO+1, and PNA also have significant regional effects. Western regions show a greater response in the distribution than ecoregions, although this could be partially explained by their small extent.

*
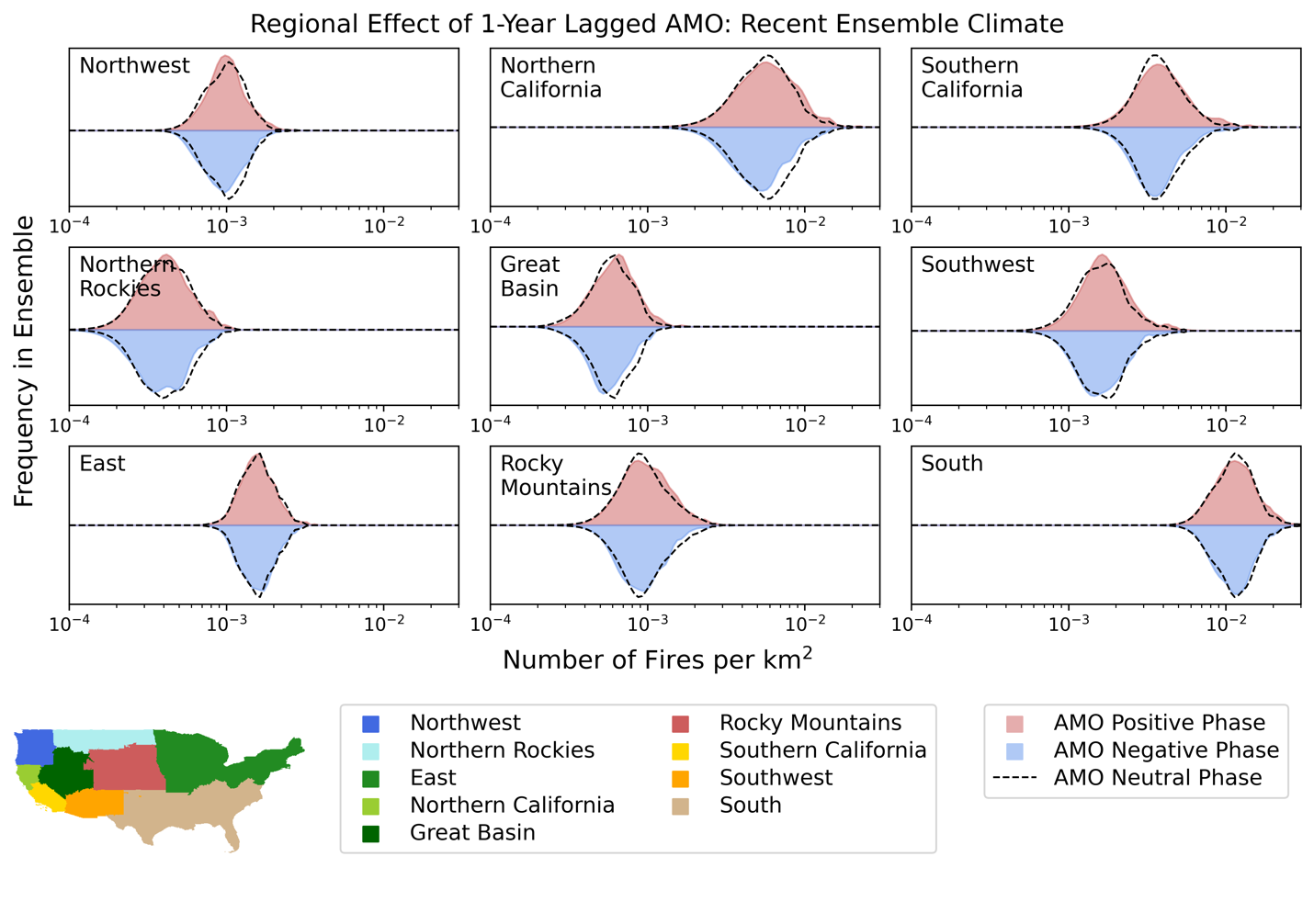
 Supplementary Figure 9.1: the effect of Atlantic Multidecadal Oscillation (AMO) on US wildfire distributions regionally.*


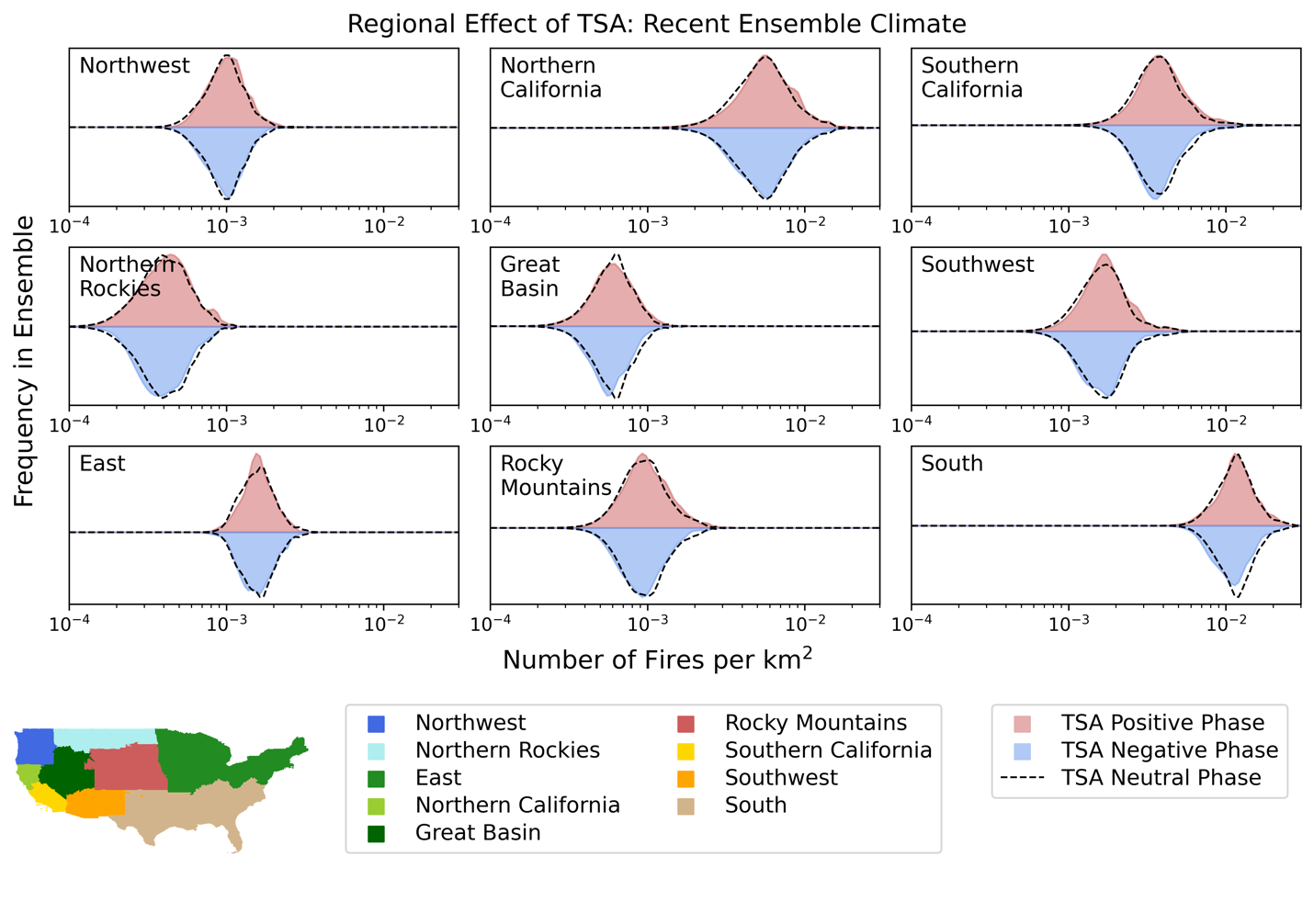


*Supplementary Figure 9.2: the effect of Tropical South Atlantic (TSA) on US wildfire distributions regionally.*


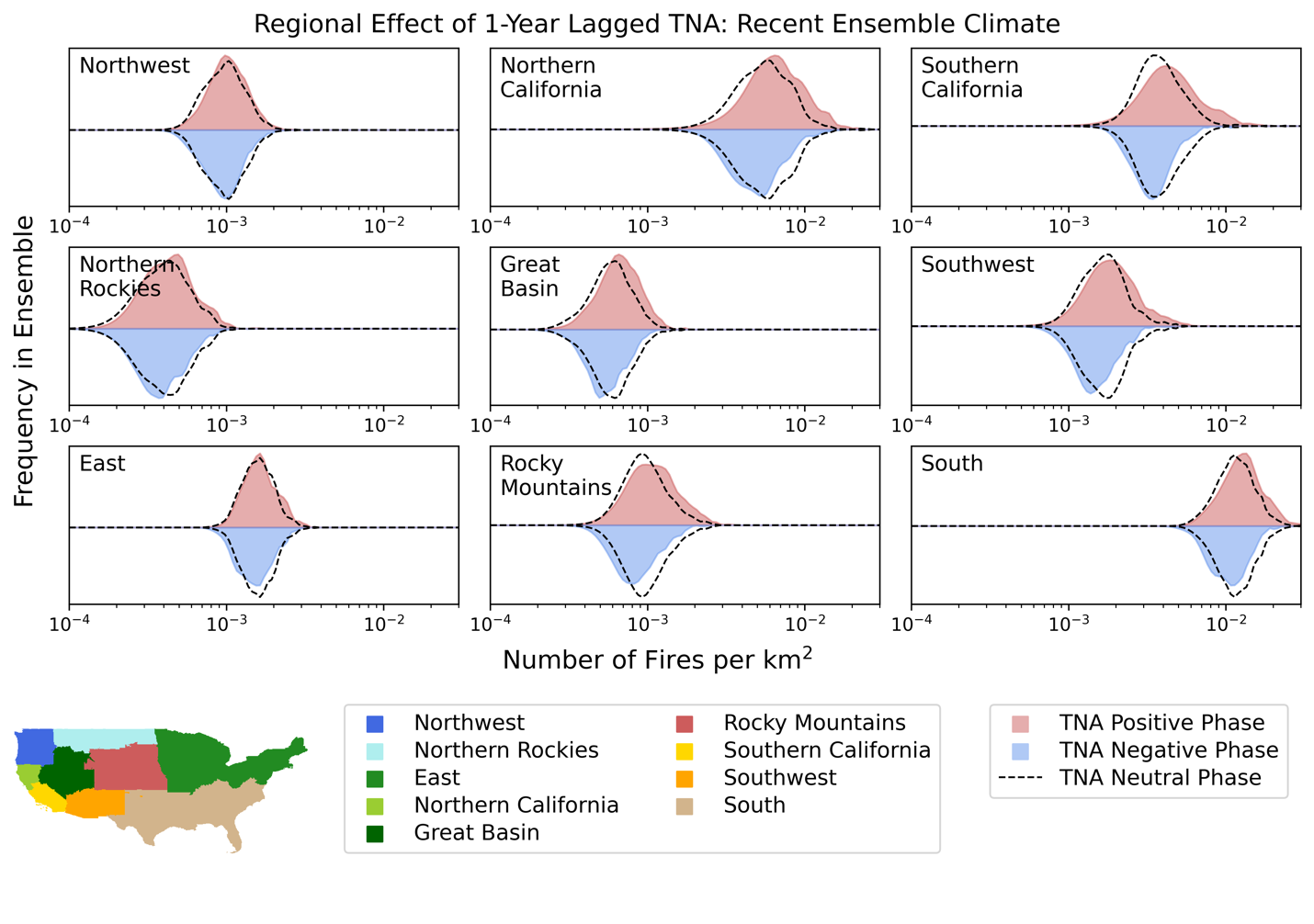


*Supplementary Figure 9.3: the effect of 1-year lagged Tropical North Atlantic (TNA+1) on US wildfire distributions regionally.*

*
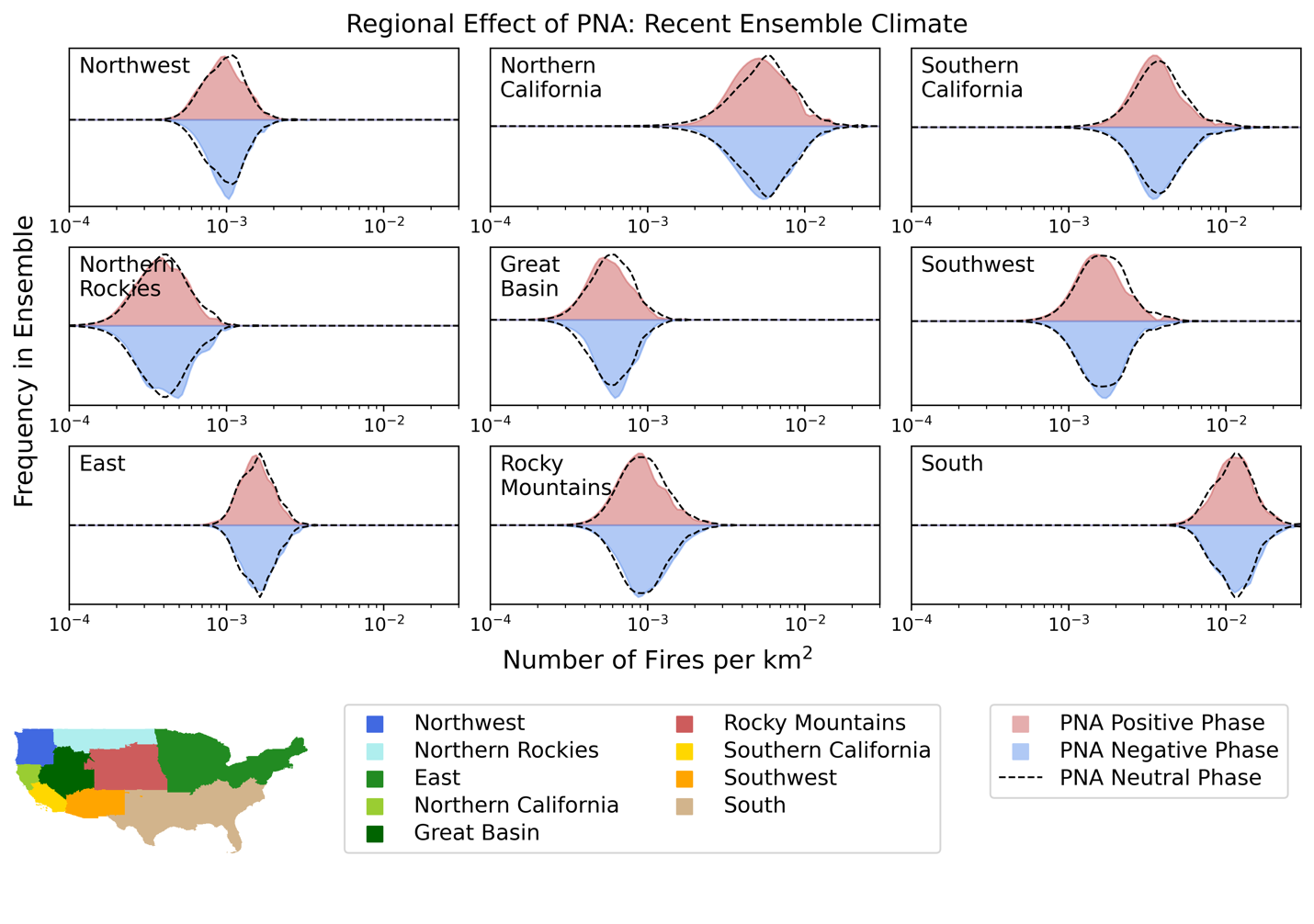
 Supplementary Figure 9.4: the effect of Pacific/North American (PNA) on US wildfire distributions regionally.*


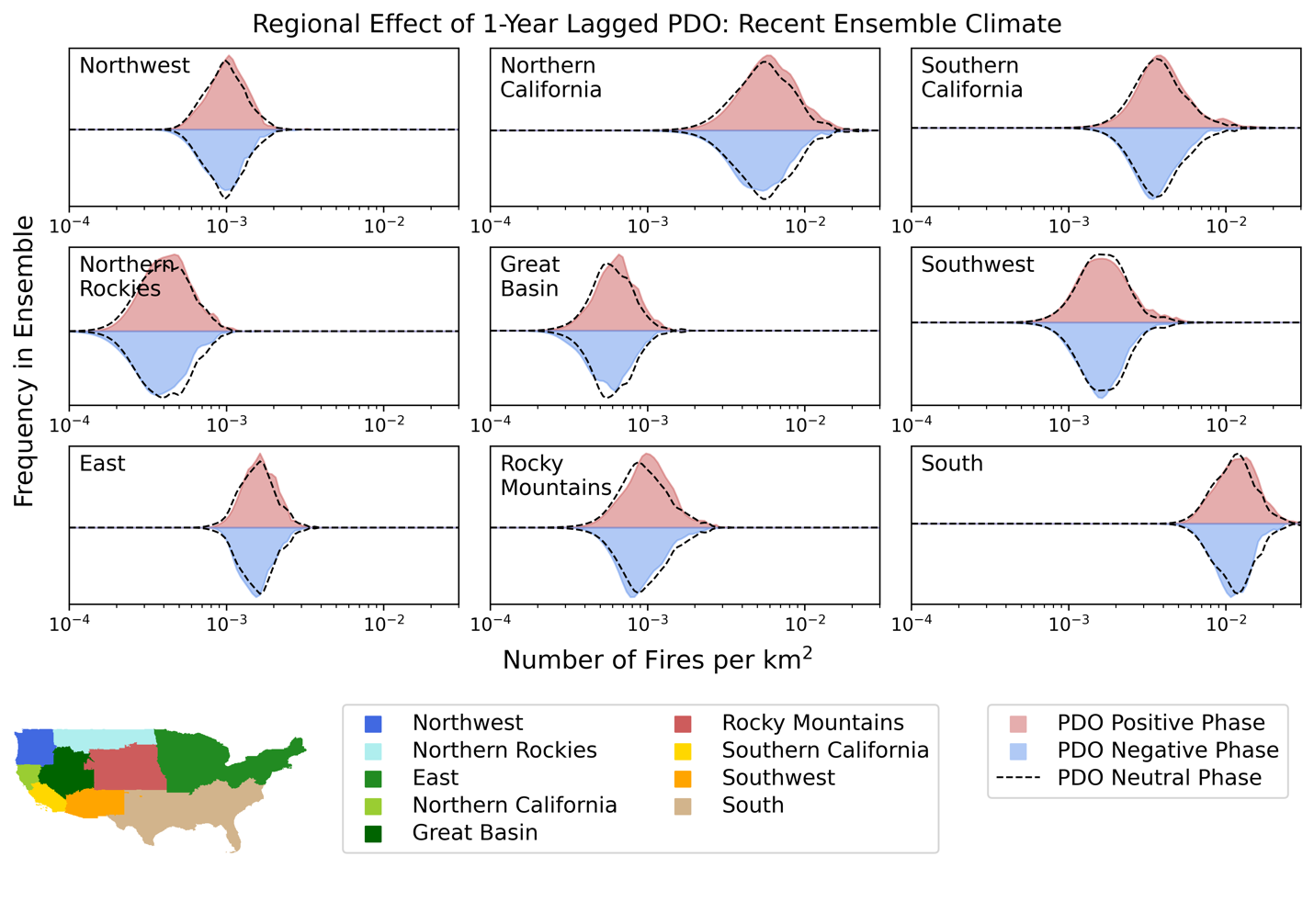


*Supplementary Figure 9.5: the effect of 1-year lagged Pacific Decadal Oscillation (PDO+1) on US wildfire distributions regionally.*

*
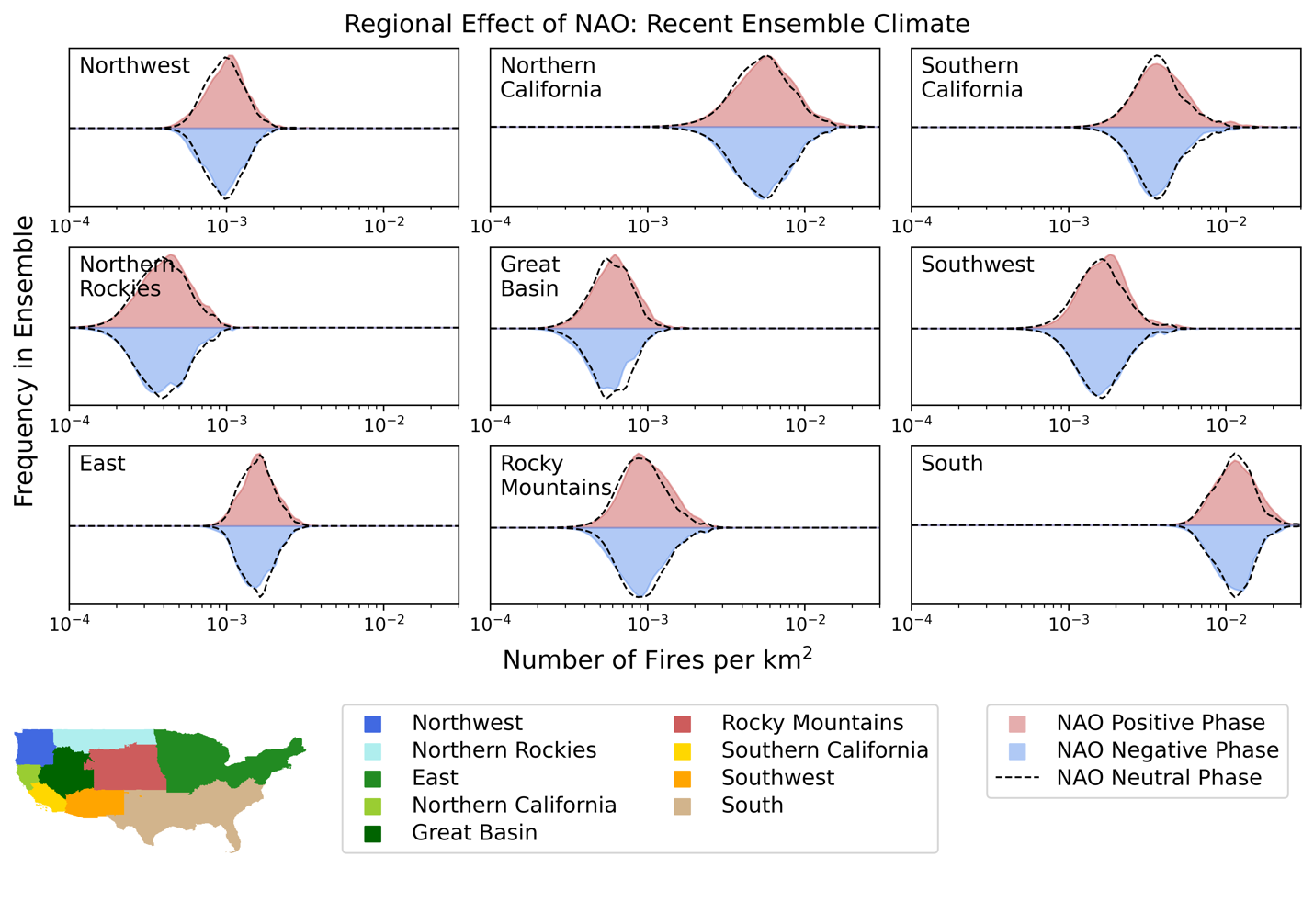
 Supplementary Figure 9.6: the effect of North Atlantic Oscillation (NAO) on US wildfire distributions regionally.*

*
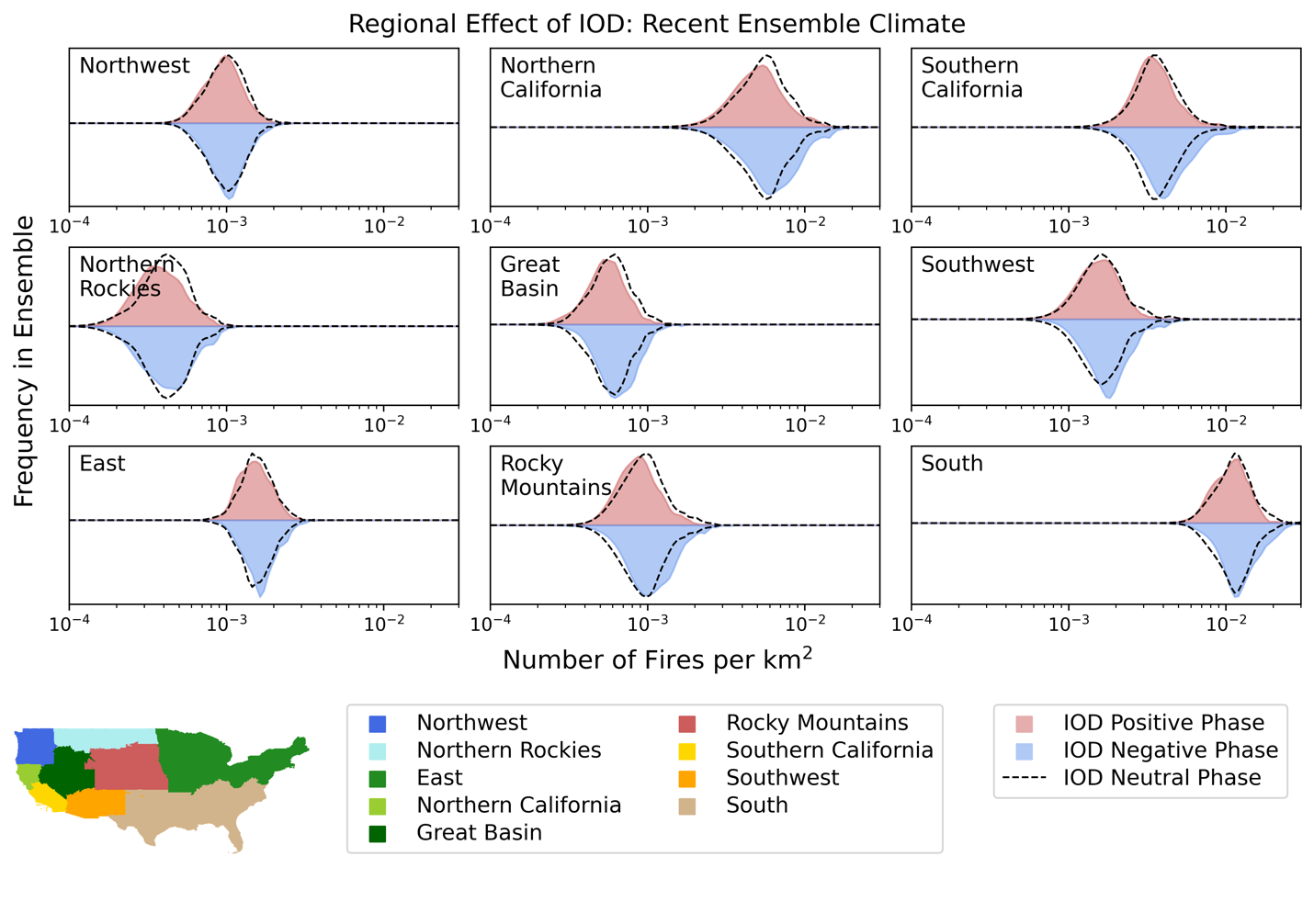
 Supplementary Figure 9.7: the effect of Indian Ocean Dipole (IOD) on US wildfire distributions regionally.*

*
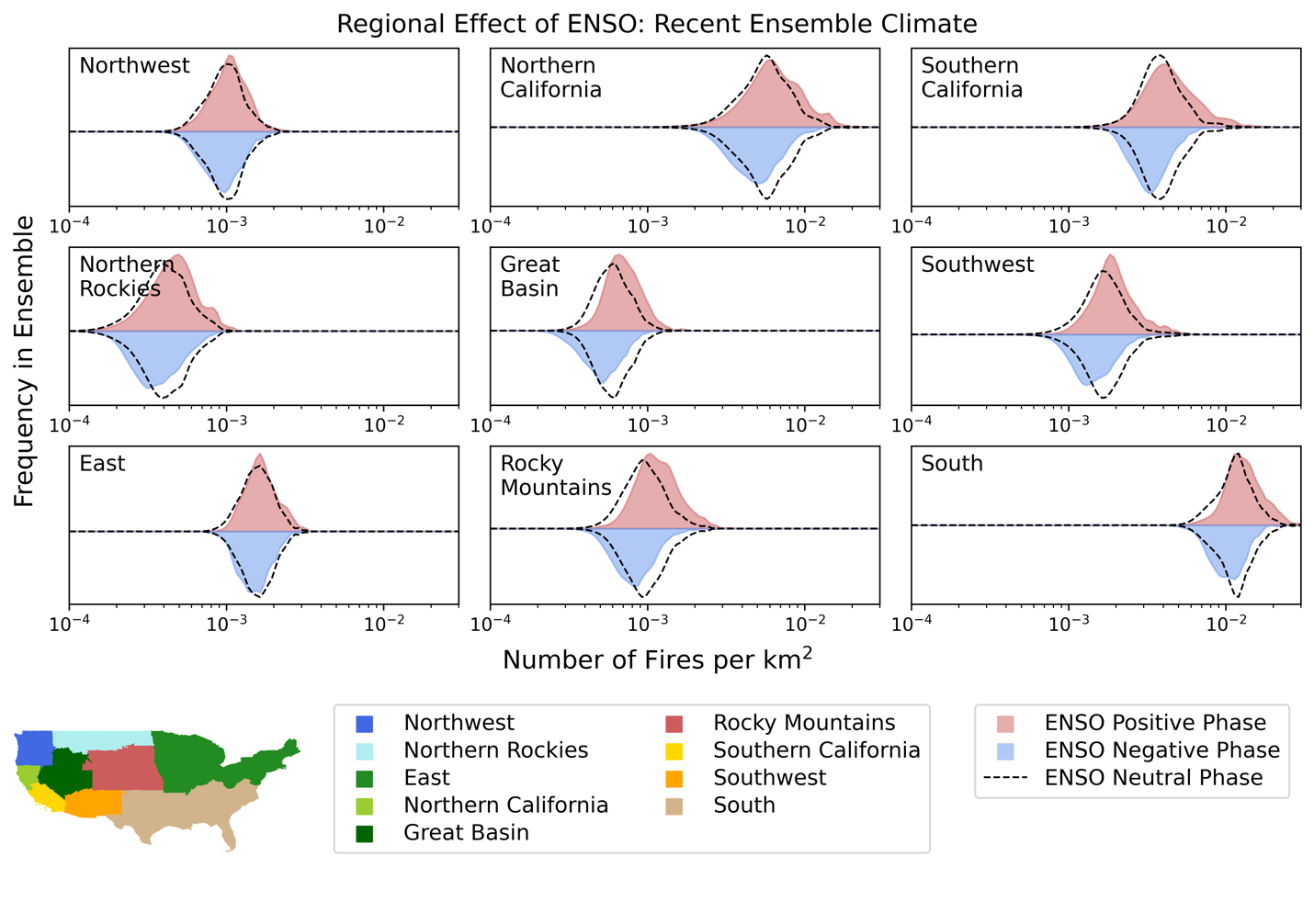
 Supplementary Figure 9.8: the effect of El Niño Southern Oscillation (ENSO) on US wildfire distributions regionally.*

### **Supplementary Section 10: Wildfire Season Length and Peak Timing Given Climate Mode Phase**

El Niño shows a strong effect on the seasonal timing, resulting in an earlier fire season peak east of the Great Plains and a later fire season peak west of the Great Plains and in the southwestern US. The reverse of this effect is apparent in La Niña years, but is not as strong in its difference from the mean. This El Niño like effect on the fire season is also apparent in the negative 1-year lagged Tropical North Atlantic (TNA+1), the positive Indian Ocean Dipole (IOD), and the negative 1-year lagged Pacific Decadal Oscillation (PDO+1) in order of decreasing strength. The negative Pacific/North American and positive North Atlantic Oscillation are also associated with a half-month earlier peak in the Californian fire season. The effect on the length of the fire season corresponds to the areas where the modes are most associated with an increase in the number of wildfires (Figure 4). This signal is strongest for the increasing effect under La Niña in the southwestern US, the Great Plains, and southern Florida, an effect also visible in the IOD and TNA+1.

*
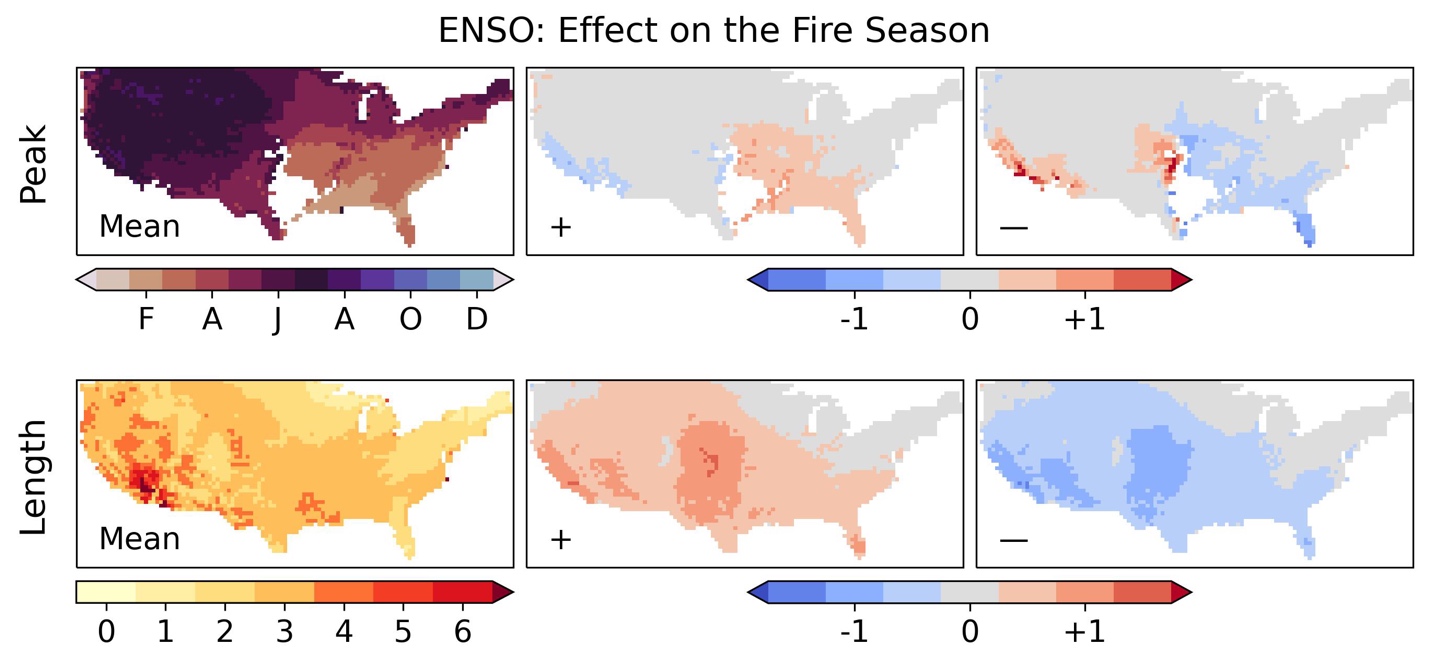
 Supplementary Figure 10.1: top row; the average seasonal phase across all locations with sufficiently high seasonal concentration (over 0.15), and the effect of the phase of El Niño Southern Oscillation (ENSO) on the timing of the seasonal peak in months. Bottom row; the length of the fire season in months (calculated as the number of months over the mean annual half-maximum) and the effect of the phase of ENSO on the season length in months.*

*
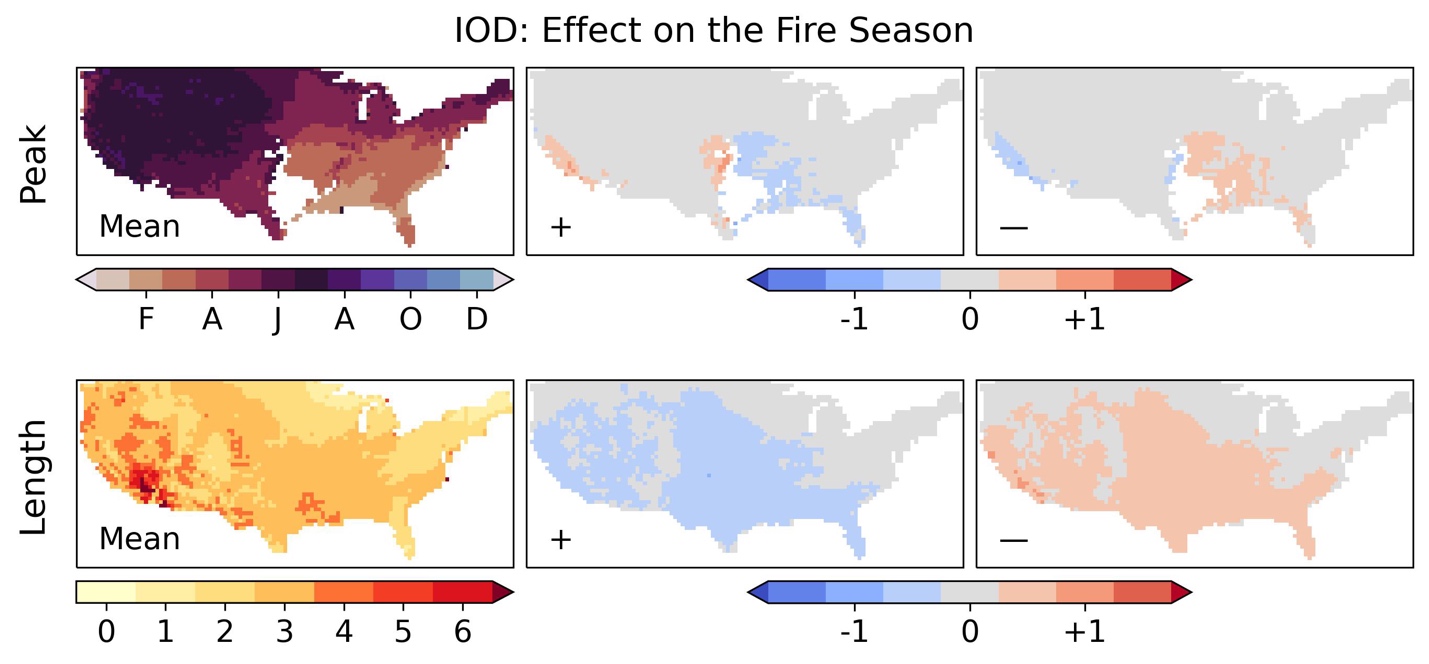
 Supplementary Figure 10.2: top row; the average seasonal phase across all locations with sufficiently high seasonal concentration (over 0.15), and the effect of the phase of the Indian Ocean Dipole (IOD) on the timing of the seasonal peak in months. Bottom row; the length of the fire season in months (calculated as the number of months over the mean annual half-maximum) and the effect of the phase of IOD on the season length in months.*

*
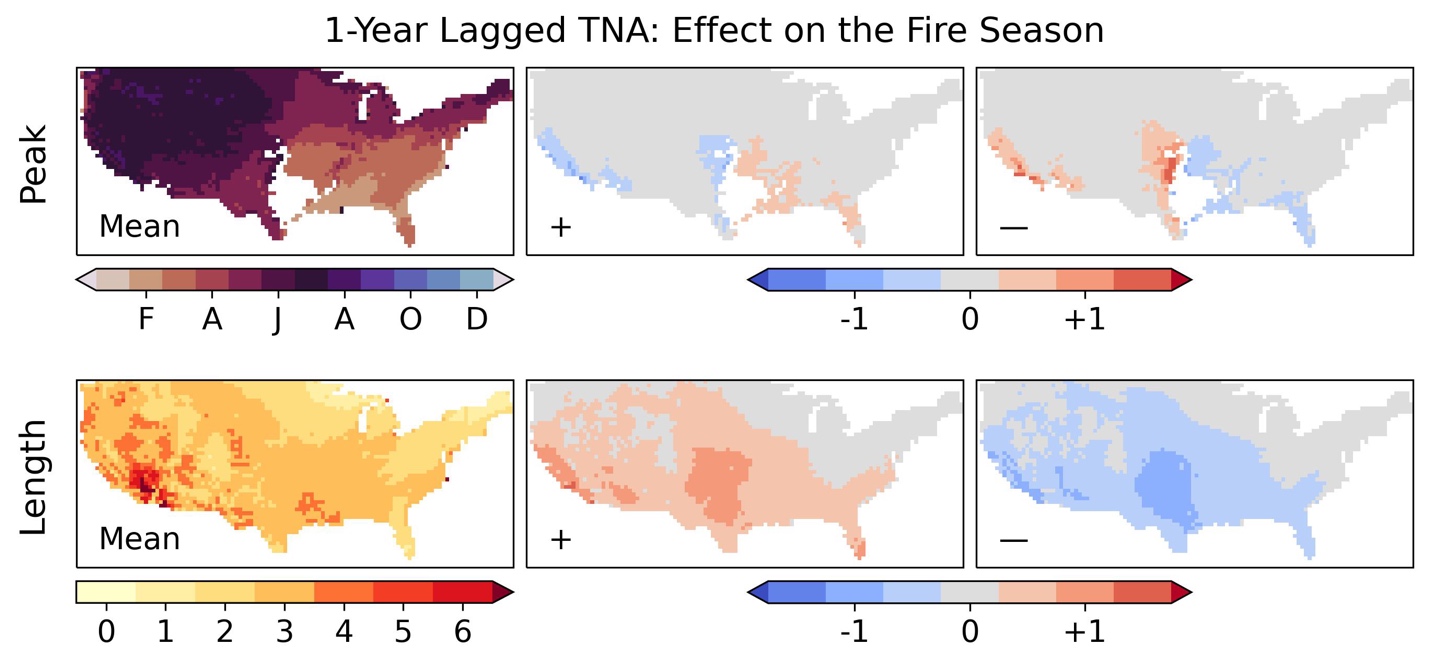
 Supplementary Figure 10.3: top row; the average seasonal phase across all locations with sufficiently high seasonal concentration (over 0.15), and the effect of the phase of the 1-year lagged Tropical North Atlantic (TNA+1) on the timing of the seasonal peak in months. Bottom row; the length of the fire season in months (calculated as the number of months over the mean annual half-maximum) and the effect of the phase of TNA+1 on the season length in months.*


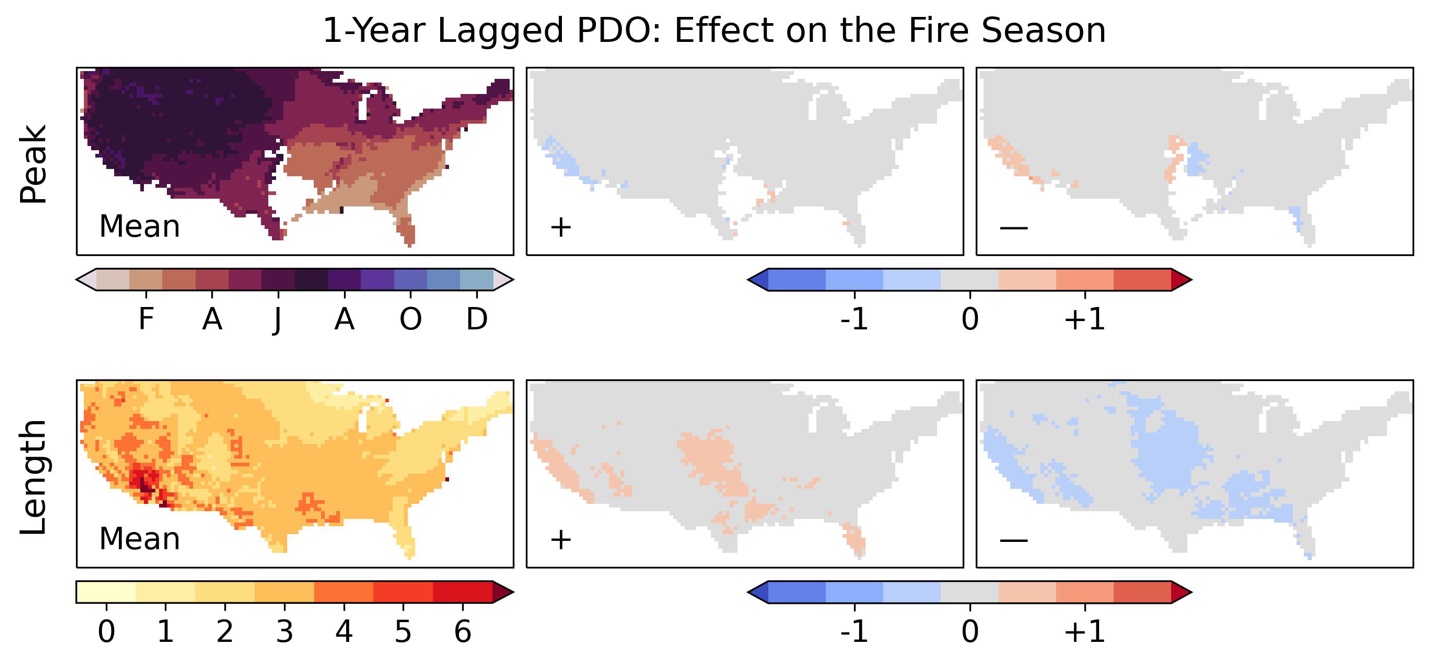


*Supplementary Figure 10.4: top row; the average seasonal phase across all locations with sufficiently high seasonal concentration (over 0.15), and the effect of the phase of the 1-year lagged Pacific Decadal Oscillation (PDO+1) on the timing of the seasonal peak in months. Bottom row; the length of the fire season in months (calculated as the number of months over the mean annual half-maximum) and the effect of the phase of PDO+1 on the season length in months.*

*
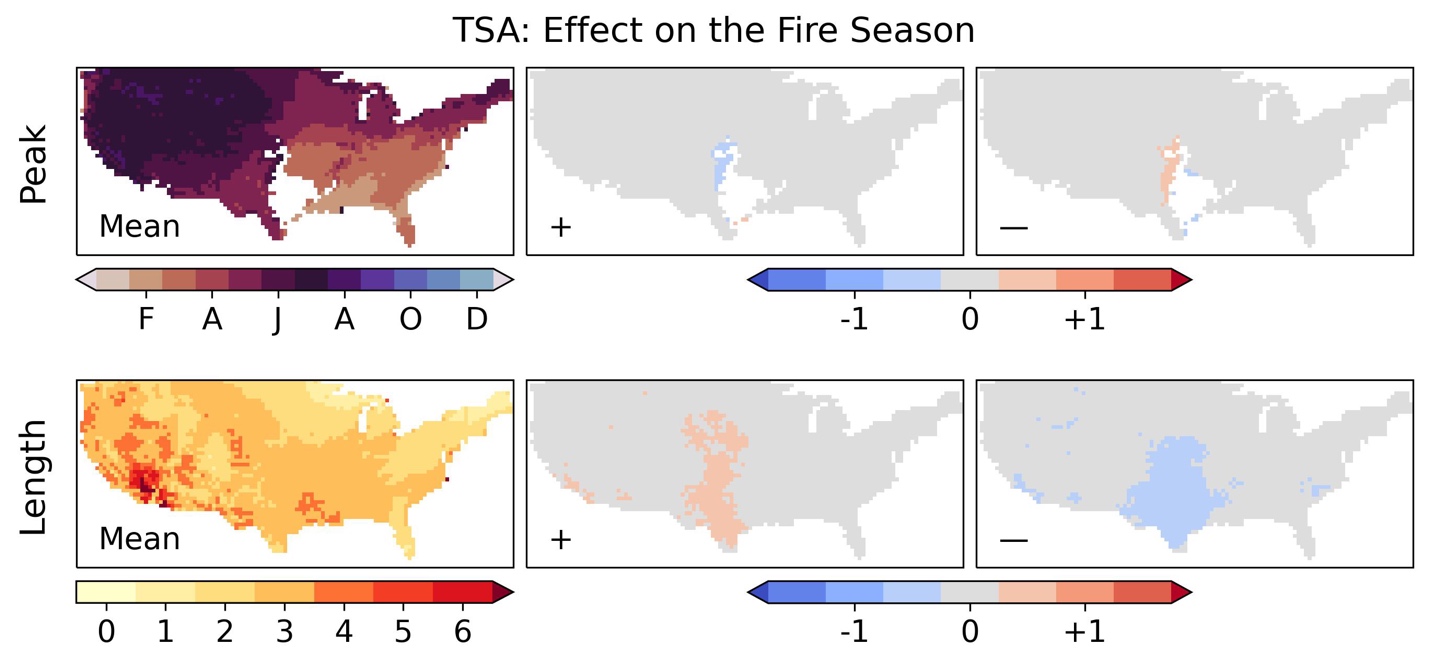
 Supplementary Figure 10.5: top row; the average seasonal phase across all locations with sufficiently high seasonal concentration (over 0.15), and the effect of the phase of the Tropical South Atlantic (TSA) on the timing of the seasonal peak in months. Bottom row; the length of the fire season in months (calculated as the number of months over the mean annual half-maximum) and the effect of the phase of TSA on the season length in months.*

*
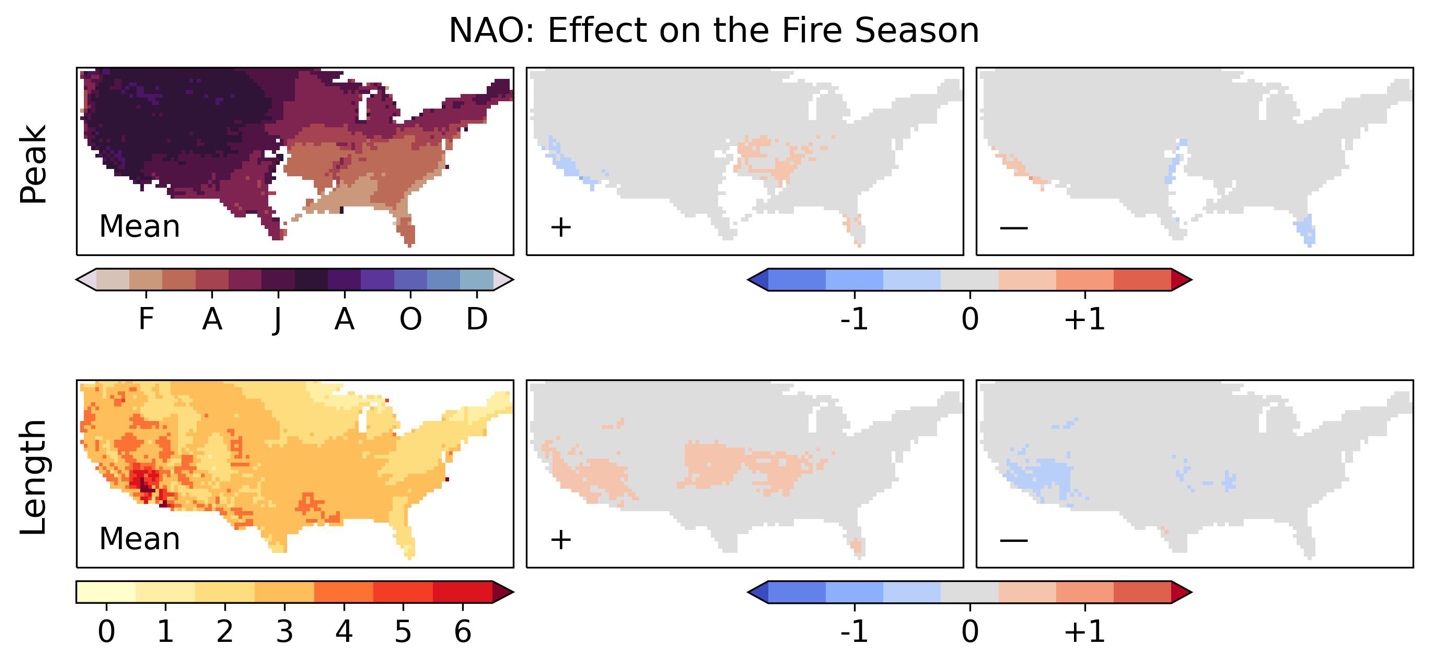
 Supplementary Figure 10.6: top row; the average seasonal phase across all locations with sufficiently high seasonal concentration (over 0.15), and the effect of the phase of the North Atlantic Oscillation (NAO) on the timing of the seasonal peak in months. Bottom row; the length of the fire season in months (calculated as the number of months over the mean annual half-maximum) and the effect of the phase of NAO on the season length in months.*

*
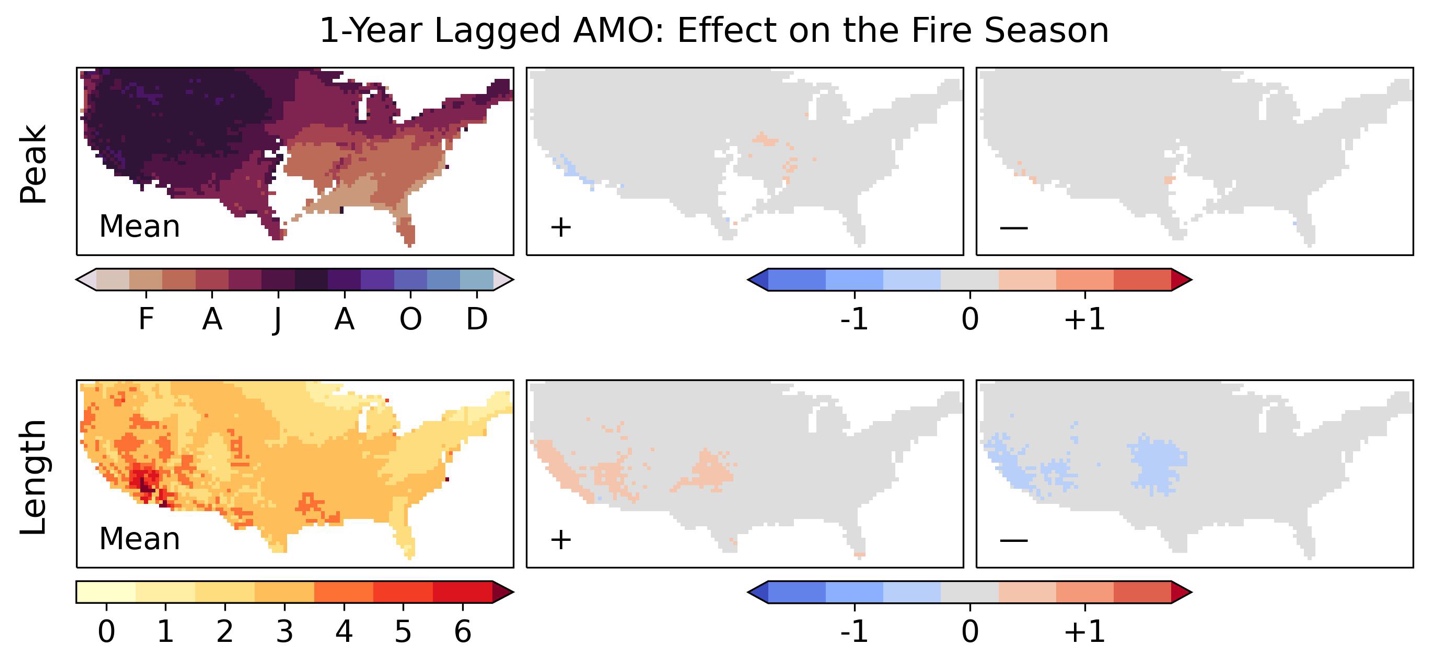
 Supplementary Figure 10.7: top row; the average seasonal phase across all locations with sufficiently high seasonal concentration (over 0.15), and the effect of the phase of the 1-year lagged Atlantic Multidecadal Oscillation (AMO+1) on the timing of the seasonal peak in months. Bottom row; the length of the fire season in months (calculated as the number of months over the mean annual half-maximum) and the effect of the phase of AMO+1 on the season length in months.*

*
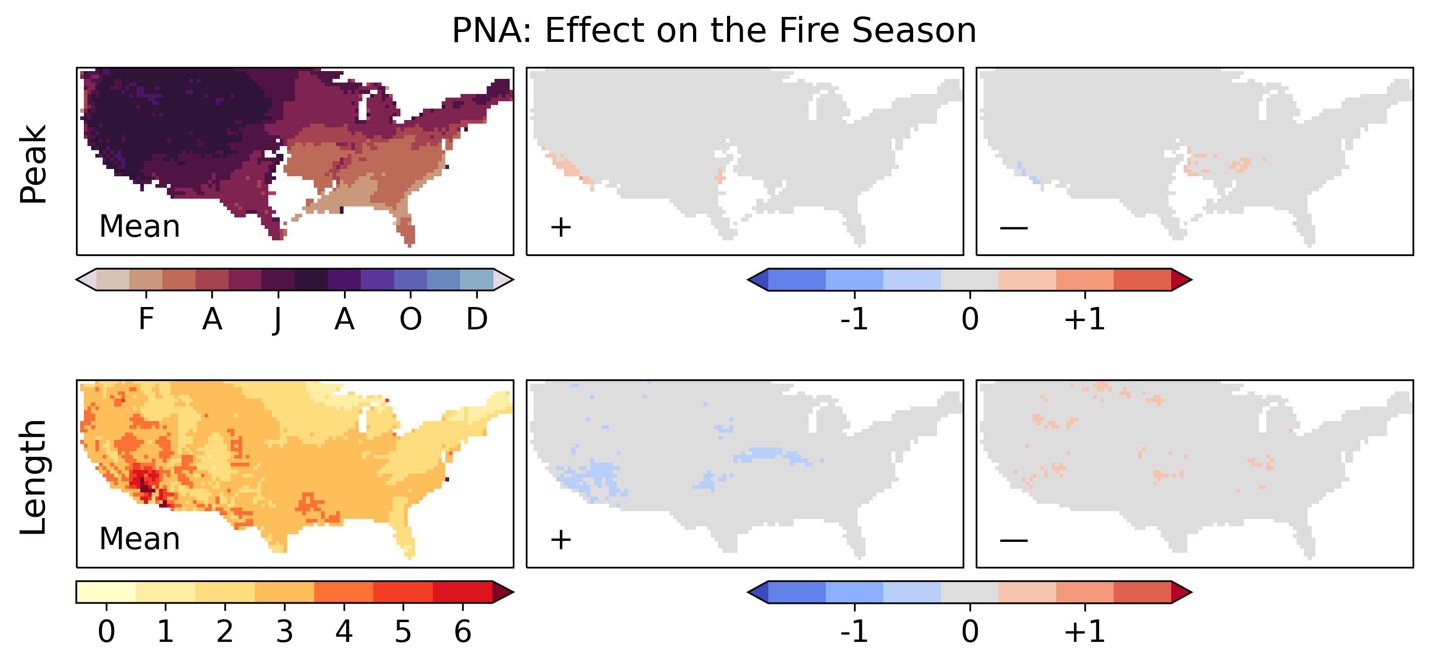
 Supplementary Figure 10.8: top row; the average seasonal phase across all locations with sufficiently high seasonal concentration (over 0.15), and the effect of the phase of the Pacific/North American (PNA) on the timing of the seasonal peak in months. Bottom row; the length of the fire season in months (calculated as the number of months over the mean annual half-maximum) and the effect of the phase of PNA on the season length in months.*

### **Supplementary Section 11: Effect of Future Climate Change on Wildfire’s Relationship with Global Climate Modes**

Supplementary Table 11.1 gives the numerical values corresponding the Figure 9 in the main text, showing the change in the areal effect of modes from the recent to +2°C climates.

Maps are then shown for the relative change in the number of wildfires for the recent and +2°C ensemble climates where the effect is significant to p<0.001; the strengthening of the effect in the regions where the relationships is significant in both climates (as shown in Figure 10) is also shown for context. In addition to the strengthening effects described in Section 3.3 of the main text, these maps show significant changes in the patterns of influence of some modes. The 1-year lagged Atlantic Multidecadal Oscillation (AMO+1) shows a very strong increase in its area and strength of effect on wildfire occurrence, covering much of the eastern US in the +2°C scenario. The Pacific/North American (PNA) and Arctic Oscillation (AO) also increase significantly in their area of effect, covering much of the central US in the +2°C climate; whilst the Tropical South Atlantic (TSA) declines to having almost no influential area.

*Supplementary Table 11.1: Percentage of contiguous US area significantly affected by each mode in the +2°C period. Three asterisks indicate a changed area over 30%, two asterisks indicate a changed area over 10%, one asterisk indicates a changed area over 5%.*

|  | | | Was Insignificant | | | Was Positive | | | Was Negative | | |
| --- | --- | --- | --- | --- | --- | --- | --- | --- | --- | --- | --- |
|  |  |  | N/A | + | - | N/A | + | - | N/A | + | - |
| Same Year | ENSO | 3.6 | | 5.0 | 0.2 | 2.0 | 88.9 | 0.0 | 0.1 | 0.0 | 0.2 |
|  | IOD | 3.6 | | 0.4 | *5.8 | 0.0 | 0.1 | 0.0 | 1.0 | 0.0 | 89.0 |
|  | PDO | 57.9 | | 2.4 | 3.1 | **17.7 | 16.4 | 1.3 | 0.7 | 0.0 | 0.5 |
|  | TNA | 19.5 | | *8.8 | **25.5 | 0.1 | 0.6 | 0.0 | *6.3 | 0.0 | 39.3 |
|  | TSA | 47.5 | | 0.0 | 0.0 | ***52.2 | 0.0 | 0.0 | 0.3 | 0.0 | 0.0 |
|  | NAO | 53.7 | | *7.8 | 4.4 | **14.7 | 19.4 | 0.0 | 0.0 | 0.0 | 0.0 |
|  | PNA | 30.0 | | 0.1 | ***43.6 | 0.0 | 0.0 | 0.0 | 2.6 | 0.0 | 23.7 |
|  | AO | 61.5 | | **28.8 | 0.0 | 1.9 | 7.8 | 0.0 | 0.0 | 0.0 | 0.0 |
|  | AMO | 79.8 | | **17.1 | 3.1 | 0.0 | 0.0 | 0.0 | 0.0 | 0.0 | 0.0 |
|  | SAM | 100.0 | | 0.0 | 0.0 | 0.0 | 0.0 | 0.0 | 0.0 | 0.0 | 0.0 |
|  | EA | 100.0 | | 0.0 | 0.0 | 0.0 | 0.0 | 0.0 | 0.0 | 0.0 | 0.0 |
| Lagged | ENSO | 22.6 | | 2.4 | **29.0 | 0.3 | 1.0 | 0.8 | *8.5 | 0.0 | 35.5 |
|  | IOD | 62.8 | | **12.4 | *7.5 | 0.1 | 3.7 | 0.0 | **12.5 | 0.4 | 0.7 |
|  | PDO | 20.9 | | *5.8 | 0.0 | *7.0 | 66.3 | 0.0 | 0.0 | 0.0 | 0.0 |
|  | TNA | 8.5 | | *5.5 | 0.0 | 1.0 | 84.7 | 0.0 | 0.3 | 0.0 | 0.0 |
|  | TSA | 66.3 | | *6.7 | 0.0 | **13.9 | 13.1 | 0.0 | 0.0 | 0.0 | 0.0 |
|  | NAO | 44.6 | | 0.0 | **22.7 | 0.2 | 0.0 | 0.0 | *5.1 | 0.0 | 27.4 |
|  | PNA | 79.5 | | **10.6 | 0.0 | 4.2 | 5.7 | 0.0 | 0.0 | 0.0 | 0.0 |
|  | AO | 97.3 | | 0.0 | 0.0 | 0.0 | 0.0 | 0.0 | 2.7 | 0.0 | 0.0 |
|  | AMO | 18.0 | | ***34.5 | 0.0 | *5.8 | 41.5 | 0.0 | 0.1 | 0.0 | 0.0 |
|  | SAM | 95.1 | | 0.0 | 0.0 | 4.9 | 0.0 | 0.0 | 0.0 | 0.0 | 0.0 |
|  | EA | 100.0 | | 0.0 | 0.0 | 0.0 | 0.0 | 0.0 | 0.0 | 0.0 | 0.0 |

*
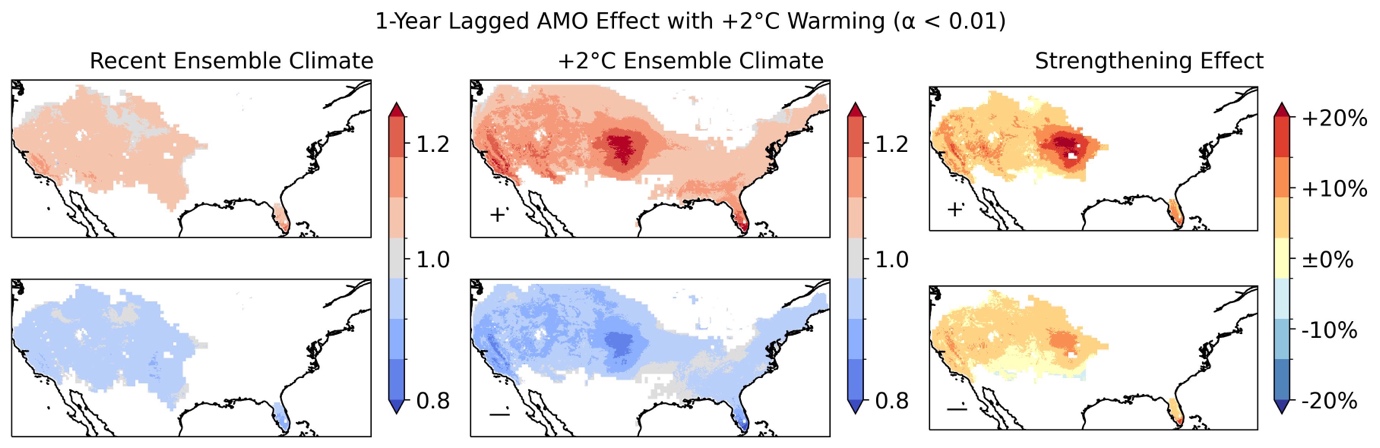
*

*Supplementary Figure 11.1: A comparison of the significant areas of effect of the 1-year lagged Atlantic Multidecadal Oscillation (AMO+1) in the recent and +2°C time periods, with the ratio of the overlapping significant areas of effect shown in the right-hand column.*

*
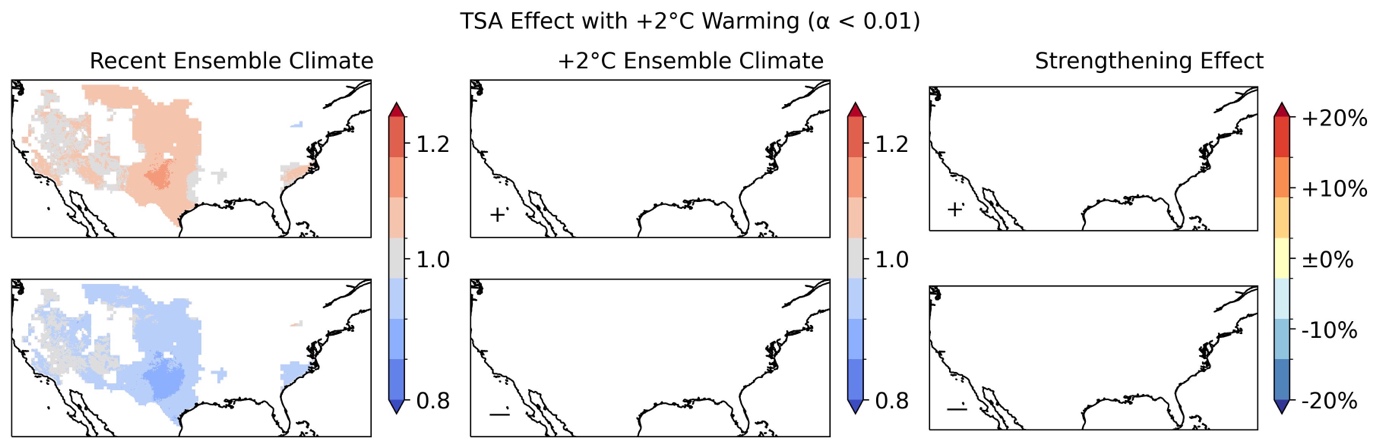
*

*Supplementary Figure 11.2: A comparison of the significant areas of effect of the Tropical South Atlantic (TSA) in the recent and +2°C time periods, with the ratio of the overlapping significant areas of effect shown in the right-hand column.*

*
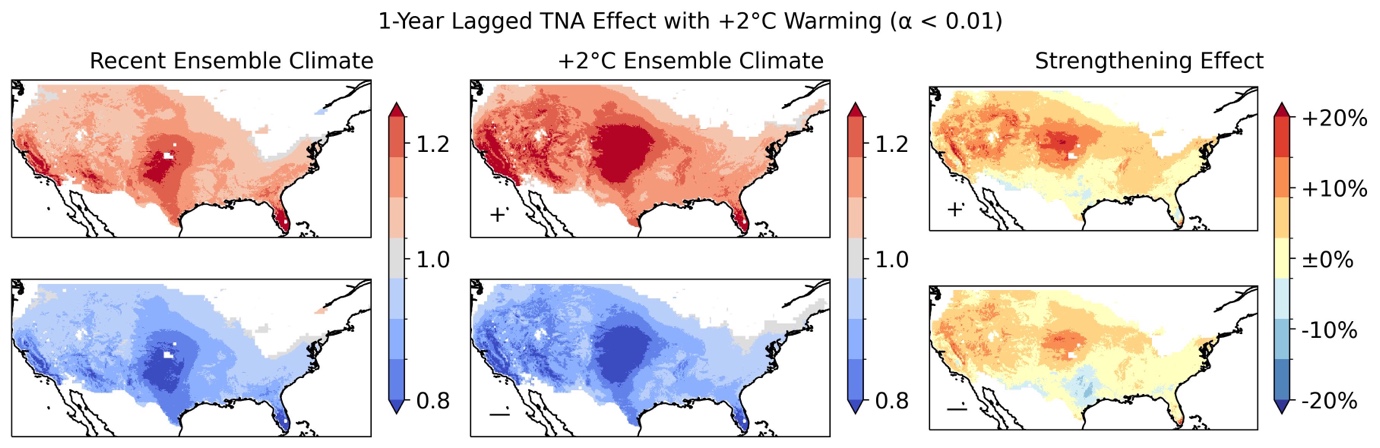
*

*Supplementary Figure 11.3: A comparison of the significant areas of effect of the 1-year lagged Tropical North Atlantic (TNA+1) in the recent and +2°C time periods, with the ratio of the overlapping significant areas of effect shown in the right-hand column.*

*
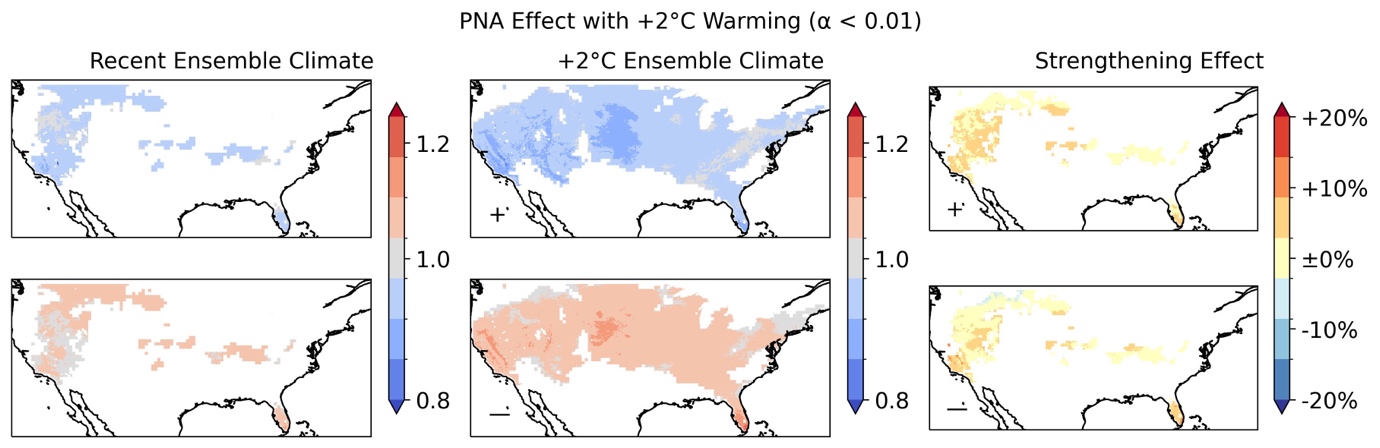
*

*Supplementary Figure 11.4: A comparison of the significant areas of effect of the Pacific/North American (PNA) in the recent and +2°C time periods, with the ratio of the overlapping significant areas of effect shown in the right-hand column.*

*
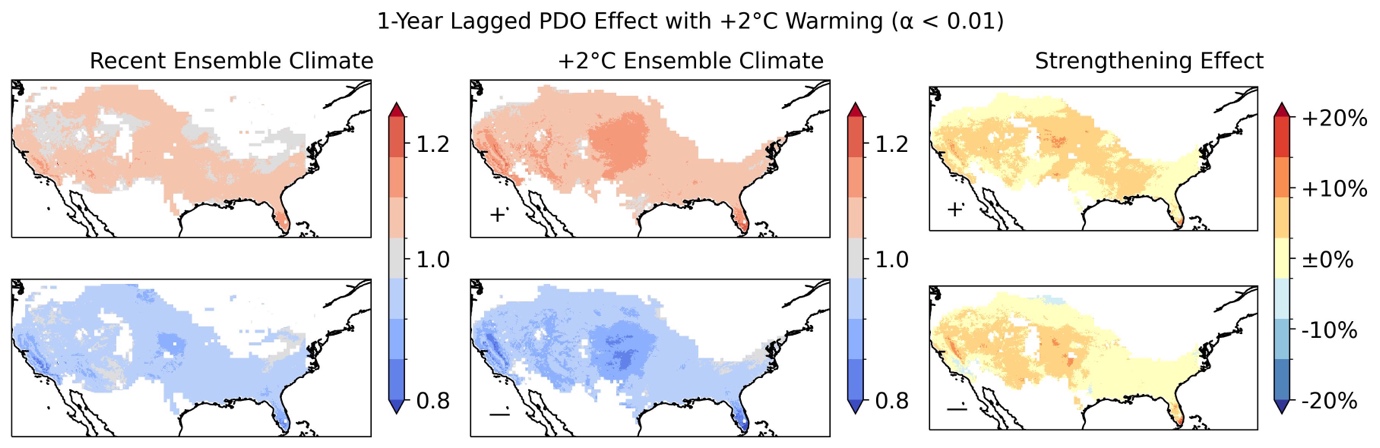
*

*Supplementary Figure 11.5: A comparison of the significant areas of effect of the 1-year lagged Pacific Decadal Oscillation (PDO+1) in the recent and +2°C time periods, with the ratio of the overlapping significant areas of effect shown in the right-hand column.*

*
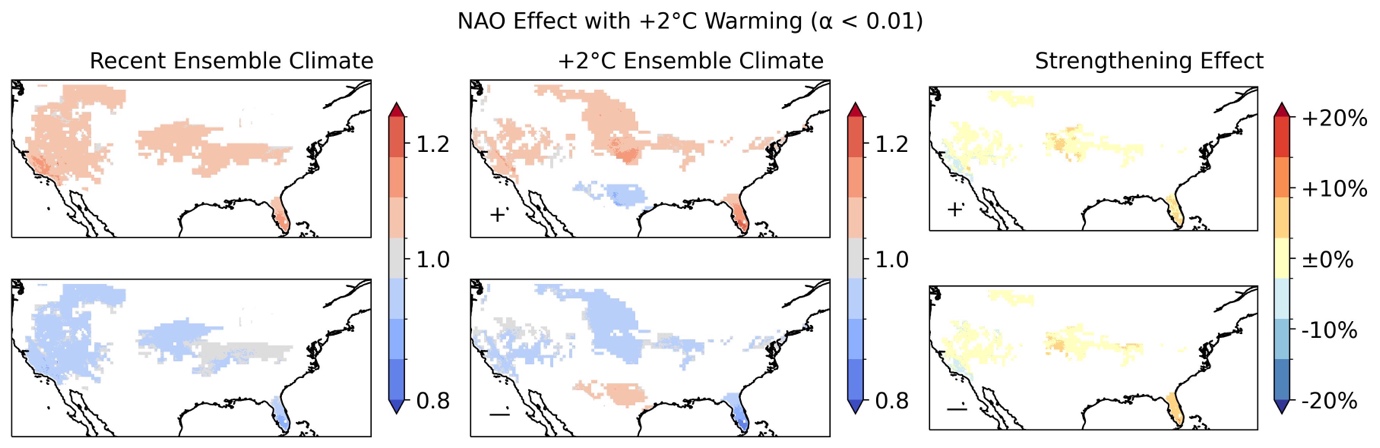
*

*Supplementary Figure 11.6: A comparison of the significant areas of effect of the North Atlantic Oscillation (NAO) in the recent and +2°C time periods, with the ratio of the overlapping significant areas of effect shown in the right-hand column.*

*
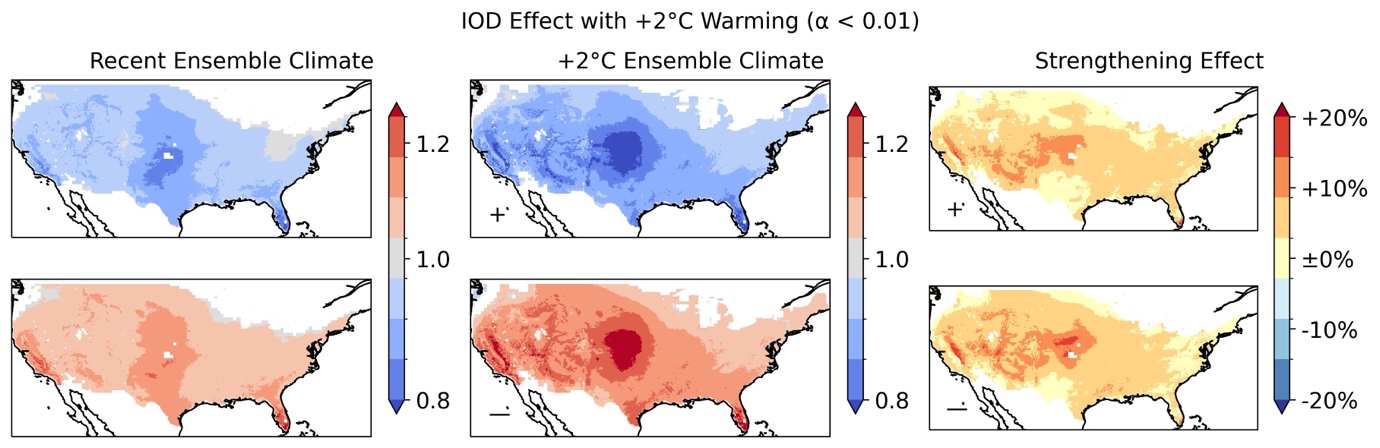
*

*Supplementary Figure 11.7: A comparison of the significant areas of effect of the Indian Ocean Dipole (IOD) in the recent and +2°C time periods, with the ratio of the overlapping significant areas of effect shown in the right-hand column.*

*
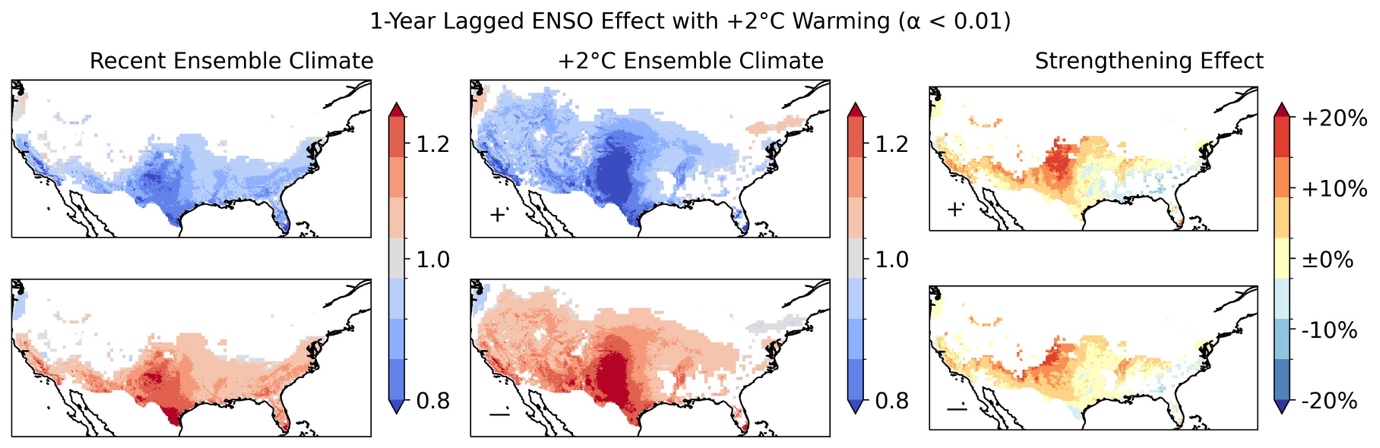
*

*Supplementary Figure 11.8: A comparison of the significant areas of effect of El Niño Southern Oscillation (ENSO) in the recent and +2°C time periods, with the ratio of the overlapping significant areas of effect shown in the right-hand column.*

*
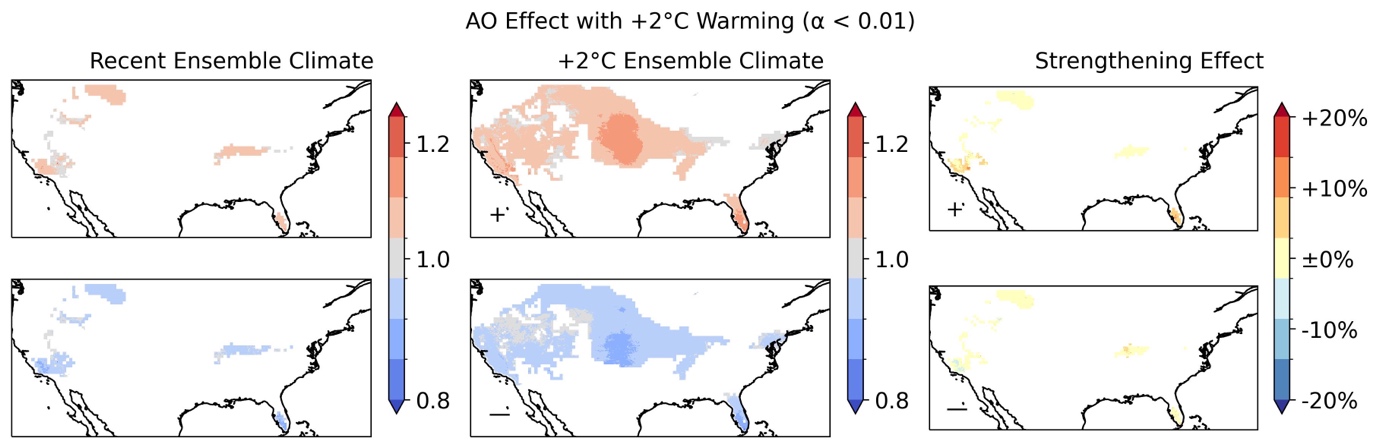
*

*Supplementary Figure 11.9: A comparison of the significant areas of effect of the Arctic Oscillation (AO) in the recent and +2°C time periods, with the ratio of the overlapping significant areas of effect shown in the right-hand column.*


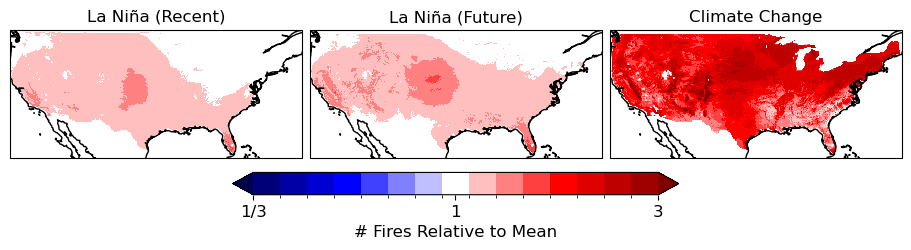


*Supplementary Figure 11.10: The magnitude of internal variability in annual fire occurrences due to a climate mode state (La Niña) relative to the mean number of fires in the recent and +2°C warmer climates in comparison to the difference between the recent and future climates. Left: the difference between the average annual number of wildfires in La Niña years and across all years in the recent (2000-2009) climate. Centre: the difference between the average annual number of wildfires in La Niña years and across all years in the +2°C warmer climate. Right: the difference between the average annual number of wildfires across all years in the recent (2000-2009) and future (+2°C warmer) climates.*

### **Supplementary References**

Ascoli D, Hacket-Pain A, LaMontagne JM, Cardil A, Conedera M, Maringer J, Motta R, Pearse IS, Vacchiano G (2020) Climate teleconnections synchronize Picea glauca masting and fire disturbance: evidence for a fire-related form of environmental pre- diction. J. Ecol. 108:1186–1198. <https://doi.org/10.1111/1365-2745.13308>

Ault T, Macalady A, Pederson G, Betancourt J, Schwartz M (2011) Northern hemisphere modes of variability and the timing of spring in western North America. J. Clim. 24:4003–4014. <https://doi.org/10.1175/2011JCLI4069.1>

Barbero R, Abatzoglou JT, Brown TJ (2015) Seasonal reversal of the influence of El Niño–Southern Oscillation on very large wildfire occurrence in the interior northwestern United States. Geophys. Res. Lett. 42:3538–3545. <https://doi.org/10.1002/2015GL063428>

Cardil A, Rodrigues M, Tapia M, Barbero R, Ramírez J, Stoof CR, Silva CA, Mohan M, de-Miguel S (2023) Climate teleconnections modulate global burned area. Nat Commun. 14:427. <https://doi.org/10.1038/s41467-023-36052-8>

Cardil A, Rodrigues M, Ramirez J, de-Miguel S, Silva CA, Mariani M, Ascoli D (2021) Coupled effects of climate teleconnections on drought, Santa Ana winds and wildfires in southern California, Sci. Tot. Env. 765:142788. <https://doi.org/10.1016/j.scitotenv.2020.142788>

Dai A (2013) The influence of the inter-decadal Pacific oscillation on US precipitation during 1923–2010. Clim. Dyn. 41(3):633-646. <https://doi.org/10.1007/s00382-012-1446-5>

Dannenberg MP, Wise EK, Janko M, Hwang T, Smith WK (2018) Atmospheric teleconnection influence on North American land surface phenology. Environ. Res. Lett. 13. <https://doi.org/10.1088/1748-9326/aaa85a>

Dixon PG, Goodrich GB, Cooke WH (2008) Using teleconnections to predict wildfires in Mississippi. Mon. Weather. Rev. 136:2804–2811. <https://doi.org/10.1175/2007MWR2297.1>

Fauria M, Johnson EA (2008) Climate and wildfires in the North American boreal forest. Philos Trans R Soc Lond B Biol Sci. 363:2317-29. <https://doi.org/10.1098/rstb.2007.2202>

Goodrick SL, Hanley DE (2009) Florida wildfire activity and atmospheric teleconnections. Int. J Wild. Fire 18:476-482. <https://doi.org/10.1071/WF07034>

Hessl AE, McKenzie DE, Schellhaas R (2004) Drought and Pacific Decadal Oscillation linked to fire occurrence in the inland Pacific Northwest, Ecol. Appl. 14(2):425–442. <https://doi.org/10.1890/03-5019>

Heyerdahl EK, Brubaker LB, Agee JK (2002) Annual and decadal climate forcing of historical fire regimes in the interior Pacific Northwest, USA. Holocene 12:597–604. <https://doi.org/10.1191/0959683602hl570rp>

Heyerdahl EK, McKenzie D, Daniels LD, Hessl AE, Littell JS, Mantua NJ (2008) Climate drivers of regionally synchronous fires in the inland northwest (1651–1900). Int. J. Wildland Fire, 17(1):40–49. <https://doi.org/10.1071/WF07024>

Hu Q, Feng S (2010) Influence of the Arctic oscillation on central United States summer rainfall. J. Geophy. Res. Atmos, 115(D1). <https://doi.org/10.1029/2009JD011805>

Hu Q, Feng S, Oglesby RJ (2011) Variations in North American summer precipitation driven by the Atlantic multidecadal oscillation. J. Clim. 24(21):5555-5570. <https://doi.org/10.1175/2011JCLI4060.1>

Hu ZZ, Kumar A, Jha B, Chen M, Wang W (2023) The tropical Indian Ocean matters for US winter precipitation variability and predictability. Env. Res. Lett. 18(7):074033. <https://doi.org/10.1088/1748-9326/ace06e>

Johnston JD, Bailey JD, Dunn CJ (2017) Historical fire-climate relationships in contrasting interior Pacific Northwest forest types. Fire Ecol. 13:18–36. <https://doi.org/10.4996/fireecology.130257453>

Justino F, Bromwich DH, Schumacher V. (2022) Arctic Oscillation and Pacific-North American pattern dominated-modulation of fire danger and wildfire occurrence. NPJ Clim. Atmos. Sci. 5:52. <https://doi.org/10.1038/s41612-022-00274-2>

Kipfmueller KF, Larson ER, St. George S (2012) Does proxy uncertainty affect the relations inferred between the Pacific Decadal Oscillation and wildfire activity in the western United States?, Geophys. Res. Lett. 39:04703, <https://doi.org/10.1029/2011GL050645>

Kitzberger T, Brown PM, Heyerdahl EK, Swetnam TW, Veblen TT (2007) Contingent Pacific-Atlantic Ocean influence on multicentury wildfire synchrony over western North America. PNAS 104:543-548, <https://doi.org/10.1073/pnas.0606078104>

Kitzberger T, Swetnam TW, Veblen TT (2001) Inter-hemispheric synchrony of forest fires and the El Niño-Southern Oscillation, Glob. Ecol. Biogeogr. 10(3):315–326, <https://doi.org/10.1046/j.1466-822X.2001.00234.x>

Kumar A, Wang H, Wang W, Xue Y, Hu ZZ (2013). Does knowing the oceanic PDO phase help predict the atmospheric anomalies in subsequent months?. J. Clim. 26(4):1268-1285. <https://doi.org/10.1175/JCLI-D-12-00057.1>

Kushnir Y, Seager R, Ting M, Naik N, Nakamura J (2010) Mechanisms of tropical Atlantic SST influence on North American precipitation variability. J. Clim. 23(21):5610-5628. <https://doi.org/10.1175/2010JCLI3172.1>

Le Goff H, Flannigan MD, Bergeron Y, Girardin MP (2007) Historical fire regime shifts related to climate teleconnections in the Waswanipi area, central Quebec, Canada. Int. J. Wild. Fire 16:607-618. <https://doi.org/10.1071/WF06151>

Leathers DJ, Yarnal B, Palecki MA (1991) The Pacific/North American teleconnection pattern and United States climate. Part I: Regional temperature and precipitation associations. J. Clim. 4(5):517-528. [https://doi.org/10.1175/1520-0442(1991)004<0517:TPATPA>2.0.CO;2](https://doi.org/10.1175/1520-0442(1991)004%3c0517:TPATPA%3e2.0.CO;2)

Mason SJ, Goddard L (2001) Probabilistic precipitation anomalies associated with ENSO. Bull. A. Met. Soc. 82(4):619-638. [https://doi.org/10.1175/1520-0477(2001)082<0619:PPAAWE>2.3.CO;2](https://doi.org/10.1175/1520-0477(2001)082%3c0619:PPAAWE%3e2.3.CO;2)

Margolis EQ, Swetnam TW (2013) Historical fire–climate relationships of upper elevation fire regimes in the south-western United States. Int. J. of Wild. Fire 22:588–598. <https://doi.org/10.1071/WF12064>

Mason SA, Hamlington PE, Hamlington BD, Jolly MW, Hoffman CM (2017) Effects of climate oscillations on wildland fire potential in the continental United States. Geophys. Res. Lett. 44:7002–7010. <https://doi.org/10.1002/2017gl074111>

Moody TJ, Fites-Kaufman J, Stephens SL (2006) Fire history and climate influences from forests in the Northern Sierra Nevada, USA. Fire Ecol 2:115–141. <https://doi.org/10.4996/fireecology.0201115>

Ning L, Bradley RS (2014) Winter precipitation variability and corresponding teleconnections over the northeastern United States. J. Geo. Res. Atmos. 119(13):7931-7945. <https://doi.org/10.1002/2014JD021591>

Norman SP, Taylor AH (2003). Tropical and north Pacific teleconnections influence fire regimes in pine-dominated forests of north-eastern California, USA. J Biogeog. 30:1081–1092. <https://doi.org/10.1046/j.1365-2699.2003.00889.x>

Rodrigo FS (2021) Exploring combined influences of seasonal East Atlantic (EA) and North Atlantic Oscillation (NAO) on the temperature-precipitation relationship in the Iberian Peninsula. Geosci. 11(5):211. <https://doi.org/10.3390/geosciences11050211>

Ropelewski CF, Halpert MS (1987) Global and regional scale precipitation patterns associated with the El Niño/Southern Oscillation. Mon. Weath. Rev. 115(8):1606-1626. https://doi.org/10.1175/1520-0493(1987)115<1606:GARSPP>2.0.CO;2

Schoennagel T, Veblen TT, Romme WH, Sibold JS, Cook ER (2005) ENSO and PDO variability affect drought-induced fire occurrence in Rocky Mountain subalpine forests. Ecol. Appl. 15:2000-2014. <https://doi.org/10.1890/04-1579>

Sibold JS, Veblen TT (2006) Relationships of subalpine forest fires in the Colorado Front Range with interannual and multidecadal-scale climatic variation, J. Biogeogr., 33(5):833–842. <https://doi.org/10.1111/j.1365-2699.2006.01456.x>

Simard AJ, Haines DA, Main WA (1985) Relations between El Nino/Southern Oscillation anomalies and wildland fire activity in the United States. Agr.For. Met. 36:93-104, <https://doi.org/10.1016/0168-1923(85)90001-2>

Simpkins G (2021). Breaking down the NAO–AO connection. Nat. Rev. Earth Env. 2(2):88-88. <https://doi.org/10.1038/s43017-021-00139-x>

Soulard N, Lin H, Yu B (2019) The changing relationship between ENSO and its extratropical response patterns. Sci. Rep. 9(1):6507. <https://doi.org/10.1038/s41598-019-42922-3>

Swetnam TW, Betancourt JL (1998) Mesoscale disturbance and ecological response to decadal climatic variability in the American Southwest. J. Clim. 11:3128–3147. [https://doi.org/10.1175/1520-0442(1998)011<3128:MDAERT>2.0.CO;2](https://doi.org/10.1175/1520-0442(1998)011%3c3128:MDAERT%3e2.0.CO;2)

Swetnam TW, Betancourt JL (1990) Fire-southern oscillation relations in the southwestern United States. Science 249:1017–1020, <https://doi.org/10.1126/science.249.4972.1017>

Tang X, Li J, Zhang Y, Li Y, Zhao S (2023) Synergistic effect of El Niño and negative phase of North Atlantic Oscillation on winter precipitation in the southeastern United States. J. Clim. 36(6):1767-1791. <https://doi.org/10.1175/JCLI-D-22-0293.1>

Trouet V, Taylor AH, Wahl ER, Skinner CN, Stephens SL (2010) Fire-climate interactions in the American West since 1400 CE. Geophys. Res. Lett. 37:L04702 <https://doi.org/10.1029/2009GL041695>

Westerling AL, Swetnam TW (2003) Interannual to decadal drought and wildfire in the western United States. Trans. AGU 84(49):545-555. <https://doi.org/10.1029/2003EO490001>
